# Human Paleodemography and Paleoecology of the North Pacific Rim from the Mid to Late Holocene

**DOI:** 10.1017/qua.2022.35

**Published:** 2022-08-05

**Authors:** Ben Fitzhugh, William A. Brown, Nicole Misarti, Katsunori Takase, Andrew H. Tremayne

**Affiliations:** 1 University of Washington, Department of Anthropology, 314 Denny Hall, Box 353100, Seattle, WA. 98195-3100;; 2 University of Washington, Department of Statistics, Box 354322 Seattle, WA 98195-4322;; 3 University of Alaska Fairbanks, Water and Environmental Research Center (WERC), PO Box 755910, Fairbanks, AK 99775-5910;; 4 Hokkaido University, Graduate School of Humanities and Human Sciences, Kita-10, Nishi-7, Sapporo, Hokkaido, Japan, 060-0810; 5 1064 Sandusky Rd. Albany Ohio, USA. 45710

## Abstract

Using 14 proxy human population time series from around the North Pacific (Alaska, Hokkaido and the Kuril Islands), we evaluate the possibility that the North Pacific climate and marine ecosystem includes a millennial-scale regime shift cycle affecting subsistence and migration. We develop both visual and statistical methods for addressing questions about relative population growth and movement in the past. We introduce and explore the use of a Time Iterative Moran I (TIMI) spatial autocorrelation method to compare time series trends quantitatively – a method that could prove useful in other paleoecological analyses. Results reveal considerable population dynamism around the North Pacific in the last 5000 years and strengthen a previously reported inverse correlation between Northeast and Northwest Pacific proxy population indices. Visual and TIMI analyses suggest multiple, overlapping explanations for the variability, including the potential that oscillating ecological regime shifts affect the North Pacific basin. These results provide an opening for coordinated research to unpack the interrelated social, cultural and environmental dynamics around the subarctic and arctic North Pacific at different spatial and temporal scales by international teams of archaeologists, historians, paleoecologists, paleoceanographers, paleoclimatologists, modelers and data management specialists.

## INTRODUCTION

In a recent paper (Fitzhugh et al., 2020), three of us reported an unexpected pattern of late Holocene human population histories in island regions across the subarctic Pacific. Using temporal frequency distributions (TFDs) of archaeological radiocarbon dates to model human population trends around the Northeast Pacific (Kodiak Archipelago, Sanak Island, and the Aleutian Islands) and the Northwest Pacific (represented by the Kuril Islands), we showed that neighboring paleodemographic models (the three NE Pacific data sets) varied synchronously over the past 2000 years in ways that were in direct opposition to those of Kuril Island series. We proposed two models to account for those patterns: a century- to millennial-scale oscillation in marine ecosystem productivity with bottom-up effects on the food security of marine-dependent hunter-gathering communities; and, alternatively (but not mutually exclusively), phased expansion of capitalist markets, commodities trade, and the incursion of virgin soil epidemics into North Pacific Indigenous communities starting in Asia and shifting to Alaska.

The purpose of this paper is to elaborate on the possible ecological mechanisms underlying the ecological model(s) and to evaluate the hypothesis that the reported proxy population trends are represented across larger areas and linked to marine economies (Fitzhugh et al., 2020). Here we ask: “Did human populations fluctuate synchronously around the North Pacific Rim at centennial to millennial scales? If they did, are these fluctuations consistent with what might be expected from ecologically ‘bottom-up’ cycles in food availability, especially in the integrated ocean system?”

Given the limitations of the four original data sets used to frame previous interpretations (outlined below), in this paper we expand the analyses to include nine additional population proxy data sets (n = 14 time series), built from archaeological radiocarbon data from around Alaska, Hokkaido, and the Kuril Islands. If the addition of these new proxy models replicate/reinforce the major findings of synchronicity (correlation and inverse correlations) in the initial study, then we can claim greater confidence in the interpretation that human demographic patterns were linked over surprisingly large spatial scales. And if those patterns replicate the cyclic patterns detected in the initial analysis, we can more reasonably consider potential environmental drivers, such as the existence of a high-amplitude, long-interval ecological oscillation structuring the Northeast and Northwest Pacific ecosystems over millennia. We reason that such a marine ecological driver should affect coastal and maritime dependent communities differently than interior/terrestrial ones (with possible shifts of population towards and away from the coast in tandem with marine cycles). And if so, expanded research is necessary to understand the ecological mechanisms to better anticipate, plan, and manage for potential large-amplitude regime shifts in future marine ecosystems that resident communities and commercial fisheries depend on for subsistence, security, and economic well-being.

## BACKGROUND

### Maritime Living around the North Pacific Rim: a brief history

**Figure 1.**
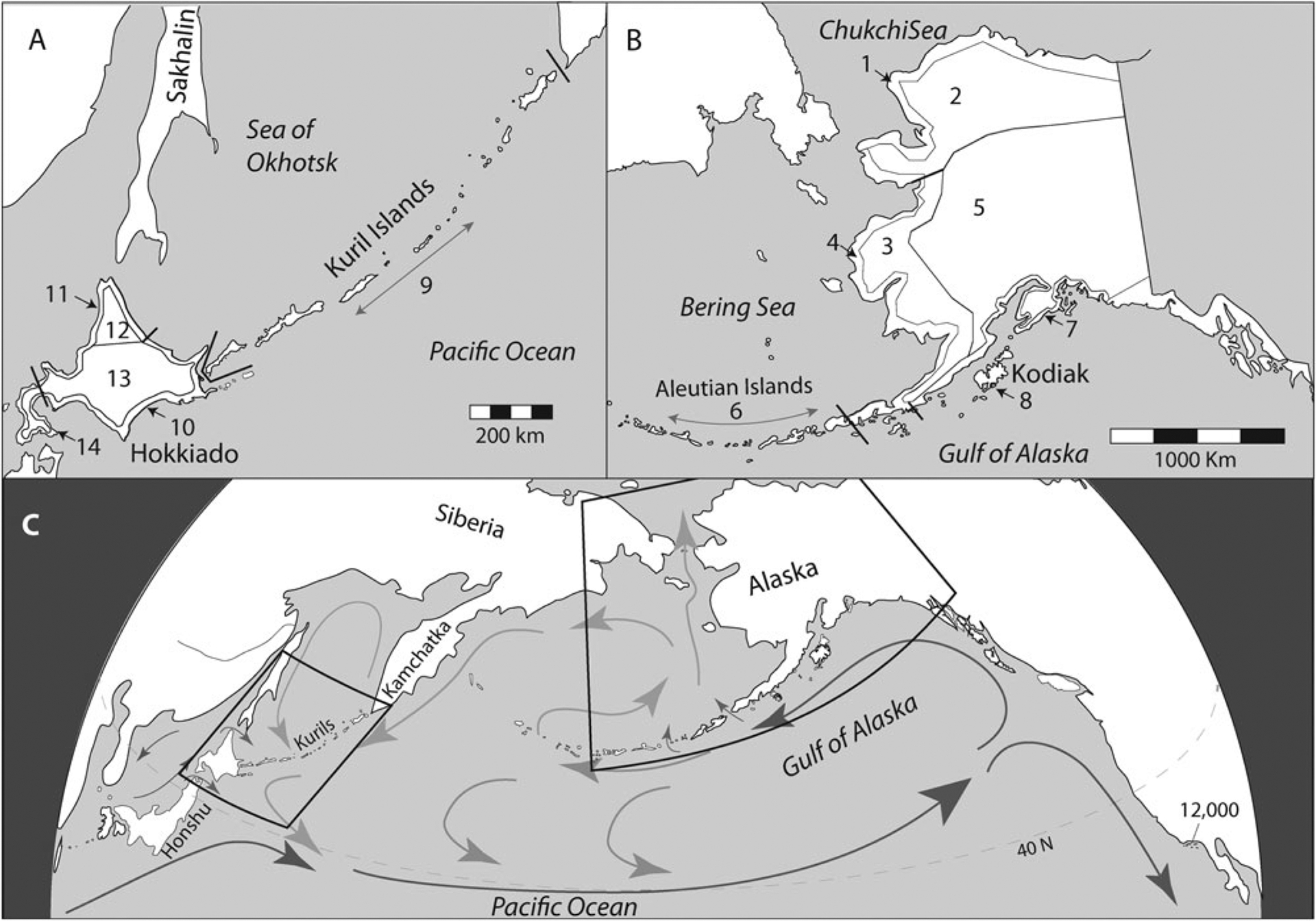
Map of N. Pacific showing region included in the current analysis. Previous comparisons (Fitzhugh et al., 2020) focused only on regions 6 (Aleutians), 8 (Kodiak), and 9 (Kurils). The current analysis expands to neighboring regions of Alaska (1–8) and Hokkaido+Kurils (9–14).

The North Pacific Rim (Fig. 1) is home to Indigenous and mixed settler communities with deep histories of interdependence with marine ecosystems. In the past, Indigenous communities from Hokkaido to the Gulf of Alaska subsisted on many of the same marine resources, captured in broadly similar ways using darts, spears, harpoons, hook and line, and nets (Fitzhugh and Crowell, 1988; Yamaura, 1998; McCartney and Veltre, 1999; Fitzhugh and Dubreuil, 1999; Steffian et al., 2006). The smaller North Pacific islands mostly lack terrestrial resources, whereas on the larger islands and mainland coasts people supplemented marine and aquatic foods with terrestrial harvests of deer, caribou, moose, and small mammals captured with arrows and darts, spears, snares, and traps.

**Figure 2.**
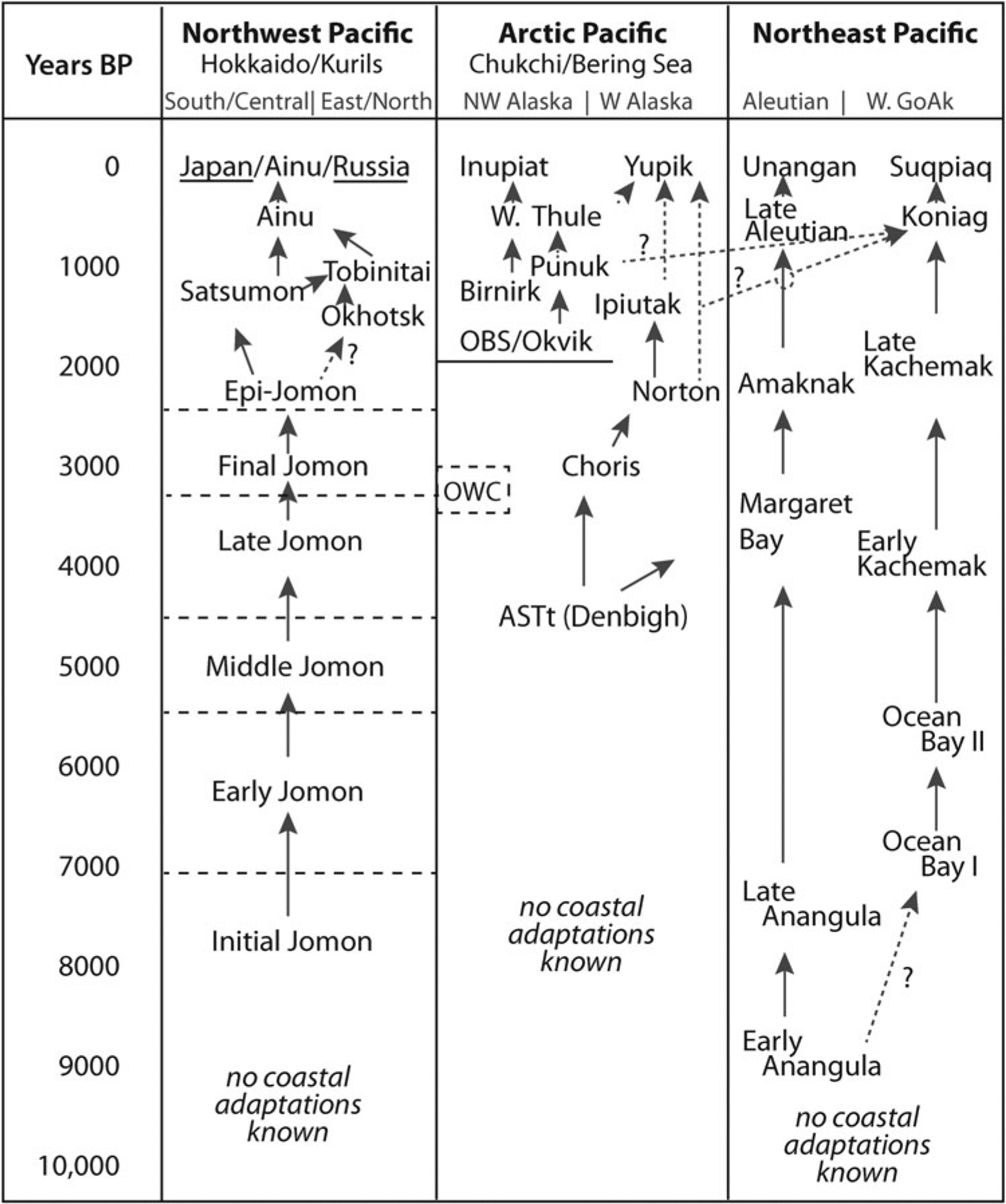
Representative culture history sequences of North Pacific archaeological cultures with maritime components found in the regions included in this analysis.

Archaeologically, the evidence for specialized maritime technologies and practices appears in the Holocene, though indirect evidence suggests possible maritime living during the last glacial maximum as well (Fitzhugh, 2022a). Coastal settlement is evident by the early Holocene in the subarctic Northeast Pacific (southern Alaska), later in the subarctic Northwest Pacific (Sea of Okhotsk), and finally in the Arctic (Bering and Chukchi Sea) regions (Fitzhugh, 2016, 2022b) (Fig. 2).

For millennia, communities in the Gulf of Alaska and Aleutians designed hand-crafted skin boats to navigate stormy and hypothermia-inducing waters. They harvested sea mammals as substantial as gray and humpback whales and fished for cod, halibut, sculpin, and greenling in nearshore environments and on the open shelf, tens of kilometers offshore. They caught salmon and other anadromous fish by the thousands with spears, weirs, nets, and traps, and they harvested shellfish, octopus, seaweeds, and other vital resources in the intertidal zones (Knecht and Davis, 2001; Fitzhugh, 2003, 2016; Corbett et al., 2010; West et al., 2012; Steffian et al., 2016).

Unangax and Sugpiaq communities have continued to harvest traditional foods through the period of Russian and American colonialism. They have also become vitally integrated into the expanding commercial fisheries markets for the export of salmon, cod, pollock, halibut, crab, and an increasing range of other products. As they have for over a century, Native and non-Native fishing crews continue to operate multi-generational fishing businesses out of villages and towns around the Gulf of Alaska and Aleutians (Reedy-Maschner, 2004; Carothers, 2012; Reedy, 2020; Carothers et al., 2021). The marine ecosystem provides food security through subsistence harvests to island and coastal villages, while commercial operations ensure that families and communities have access to cash-based essential resources and services.

On the other side of the North Pacific, the subarctic waters of northeast Hokkaido, Sakhalin, mainland coasts of the Okhotsk Sea, the Kuril Islands and Kamchatkan peninsula are occupied today by a mix of settlers (mostly Japanese, Russians, and Koreans) and Indigenous Ainu, Orok, Nivkhi, Even, Koryak, and Itel’men descent groups (Fitzhugh and Crowell, eds., 1988). In comparison to subarctic Alaskan coasts, the Asian Pacific Northeast shores experience stronger seasonal climate swings, cooled in the winter by bitter winds from Siberia and the circulation of chilled waters and pack ice along the eastern coast of the Bering Sea and from the northern Sea of Okhotsk to the shores of Hokkaido. Whale, walrus, seal and sea lion, salmon, cod and other marine fish, shellfish, and macroalgae were consumed along with terrestrial game and plants, where available. Historically, the Ainu used their intimate expertise of wild harvesting to supply products such as sea mammal furs, hawk and eagle feathers, and fish for trade to central Japan (Hudson, 1999). Okhotsk people also became active intermediaries in the trade of commodities between the wealthy states of China and Manchuria on the one hand and Japan on the other (Hudson, 1999; Walker, 2001; Tezuka, 2009). Indigenous and non-Indigenous communities from Hokkaido and the Russian Far East participate in extensive commercial fish, shellfish, and seaweed harvesting today, much of it bound for the expansive Japanese market as well as global consumption.

In contrast to the subarctic zones of Alaska and the southern Okhotsk Sea, the seasonally frozen northern Okhotsk, and around the Bering and Chukchi Seas, maritime economies were developed no earlier than 4000–5000 years ago (Tremayne and Rasic, 2016), becoming specialized on marine mammal hunting and fishing after 3500 yr (Dumond and Bland, 1995; Fitzhugh, 2016, 2022b; Gusev, 2022). On these coasts, people have since hunted ice-adapted seals, walrus, and whales from land-fast and pack ice, cracks in the ice (leads), and open water. They harvested (and continue to harvest) Arctic char and salmon from local rivers, terrestrial mammals (especially reindeer/caribou), and migratory birds as part of seasonal subsistence cycles. These animals were major staples for those living on the Bering and Chukchi Sea coasts, in the Brooks Range and up the rivers like the Anadyr, Kobuk, Noatak, Yukon, and Kuskokwim. Throughout these regions, mixtures of terrestrial and aquatic foods continue to be important for subsistence and food security of Iñupiat and Yupiit. In the post-contact era, commercial fisheries are much more recent and limited compared to the regions around the Gulf of Alaska and Aleutians.

As we see, residents of the North Pacific Rim adapted to and developed varying degrees of dependence on marine food resources through the Holocene. Those living in subarctic island environments were most fully dependent on the marine environment for their livelihoods and have the deepest histories of Indigenous fisheries, but even those communities living in the interior zones were linked to marine ecosystems through their reliance on anadromous fish like salmon and char. Where and when terrestrial game such as caribou, moose, deer, and migrating birds were plentiful, economies were less tethered to the productivity of the oceans. We expect populations closer to the coasts and especially on the islands of the subarctic Kodiak, Aleutians, and Kurils to have been most intimately influenced by changing marine ecological conditions.

### Synoptic Feedbacks in the Climatology, Oceanography, and Marine Ecology of the North Pacific Subarctic Gyre and Marginals Seas Today

In the realm of natural resource policy, memories tend to be short and reference intervals for federal and state resource management even shorter, often based on trends observed over only a few years or decades. This leads to the handicap known as the “shifting baseline syndrome” (Jackson and Alexander, 2011; McClenachan et al., 2012; Klein and Thurstan, 2016; Eckert et al., 2018). Historical analyses have revealed cycles of ecological variability otherwise missed in the annual to decade timeframes. Longer interval data, from multi-decadal to centennial to millennial could better inform managers on the ranges of variability that need to be considered in developing scenarios of the future that we know will not be similar to the past century.

At the shorter of these time scales, it is already well established that coupled North Pacific climate and ocean dynamics affect marine food webs and commercial harvests at multi-decadal scales. For example, the Pacific Decadal Oscillation (PDO) index tracks spatial anomalies in sea surface temperatures (SST) across the North Pacific (Schneider and Cornuelle, 2005; Newman et al., 2016). With annual records going back to the early 20th century, the PDO index reveals a multi-decadal oscillation in dominant and spatially discriminated warm and cold regimes. Importantly for our purposes, the PDO pattern was discovered through an effort to explain inversely correlated trends in salmon catch records from south to north along the North American West Coast (Francis et al., 1998; Mantua and Hare, 2002). When temperatures were lower than the century average for most years of a decade (or more) along the West Coast of North America, salmon catches increased for fishers in Washington, Oregon, and California but declined in the Gulf of Alaska. Catch trends reversed in warmer years.

The PDO is just one index used to understand complex climate-ocean-ecology variation in the North Pacific (Furtado et al., 2012), and recent synthesis studies have concluded that PDO/PDO-like variability arises from a combination of drivers, situated both within the North Pacific and in the tropics (Newman et al., 2016). Locally, North Pacific multi-decadal SST variability is closely connected to the dynamical strength and trajectory of the winter Aleutian Low (AL) pressure system. The AL is in turn influenced by the differential of continental vs. oceanic cooling in winter and atmospheric anomalies elsewhere in the northern hemisphere circulation system, including the El Niño Southern Oscillation (ENSO) and the East Asian Jet Stream (EAJS) (Chavez et al., 2011; Newman et al., 2016; Nagashima et al., 2021). PDO-like variability manifests as spatially patterned anomalies of warm and cold surface water across the North Pacific that reverse sign (flipping warm for cold and cold for warm) at some approximate interval. Accordingly, decades that are anomalously warm off the coast of western North America are anomalously cold around Japan, and vice versa (Chavez et al., 2011). These differences are correlated with the productivity of commercial fisheries (e.g., salmon, anchovies, sardines) on both sides of the North Pacific basin. Chavez and colleagues (2003) see these patterns resulting from climate forcing effects on bottom-up processes tied to currents and upwelling, nutrient supply, primary production, and trophic amplification.

Because detailed, formalized scientific data collection started in this region only in the early 20th century, understanding of these patterns is limited in time and focused on seasonal, annual, and decadal variability and in the context of intensive commercial harvesting (Pennoyer, 1979; Hilborn, 2012). A century of data on North Pacific ecological fluctuation is insufficient to clarify the potential range of variability that the integrated North Pacific marine ecosystem is capable of, especially under rapidly changing climate conditions. A variety of analyses have explored the time depth of North Pacific climate variability using proxy records such as tree rings (Biondi et al., 2001; MacDonald and Case, 2005; D’Arrigo et al., 2005; D’Arrigo and Wilson, 2006; Yasuda, 2009; Barron and Anderson, 2011). They indicate that longer interval climate fluctuations have altered the intensity of decadal-scale (PDO-like) variability over century to millennial time scales, with implications for the spatial evolution of climate patterns in the past (e.g., Barron and Anderson, 2010; Nagashima et al., 2021).

Despite evidence linking climate and ecology in the late 20th century, the factors controlling ecosystem change in the North Pacific remain poorly understood (Beamish and Bouillon, 1993; Hare and Francis, 1995; Hare et al., 1999; Overland and Stabeno, 2004). In particular, the complexity of the relationship between long- and short-term ecosystem dynamics are highlighted by recent paleoecological research showing that sockeye salmon (*Oncorhynchus nerka*) populations in the Gulf of Alaska *increased* over century to millennial scales in times of enhanced storminess and anomalously cool sea-surface temperatures (SSTs; as during the Little Ice Age) and *decreased* in warmer time periods (such as the Medieval Climate Anomaly) (Misarti et al., 2009; Finney et al., 2010). This is in direct contrast to 20^th^ century PDO–salmon relationships. Such studies make clear that the historical record of ecosystem response to climate variability is too short, and that the paucity of long-term data impedes our ability to forecast future ecosystem changes or to discriminate between the compounded effects of natural variability and anthropogenic influences. Fortunately, the record of human interaction with marine ecosystems goes back thousands of years, giving us access to a deeper archive of information about both ecosystems in the past and human interaction with those systems (Misarti et al. 2009).

### Synoptic Changes based on Holocene Proxies

**Figure 3.**
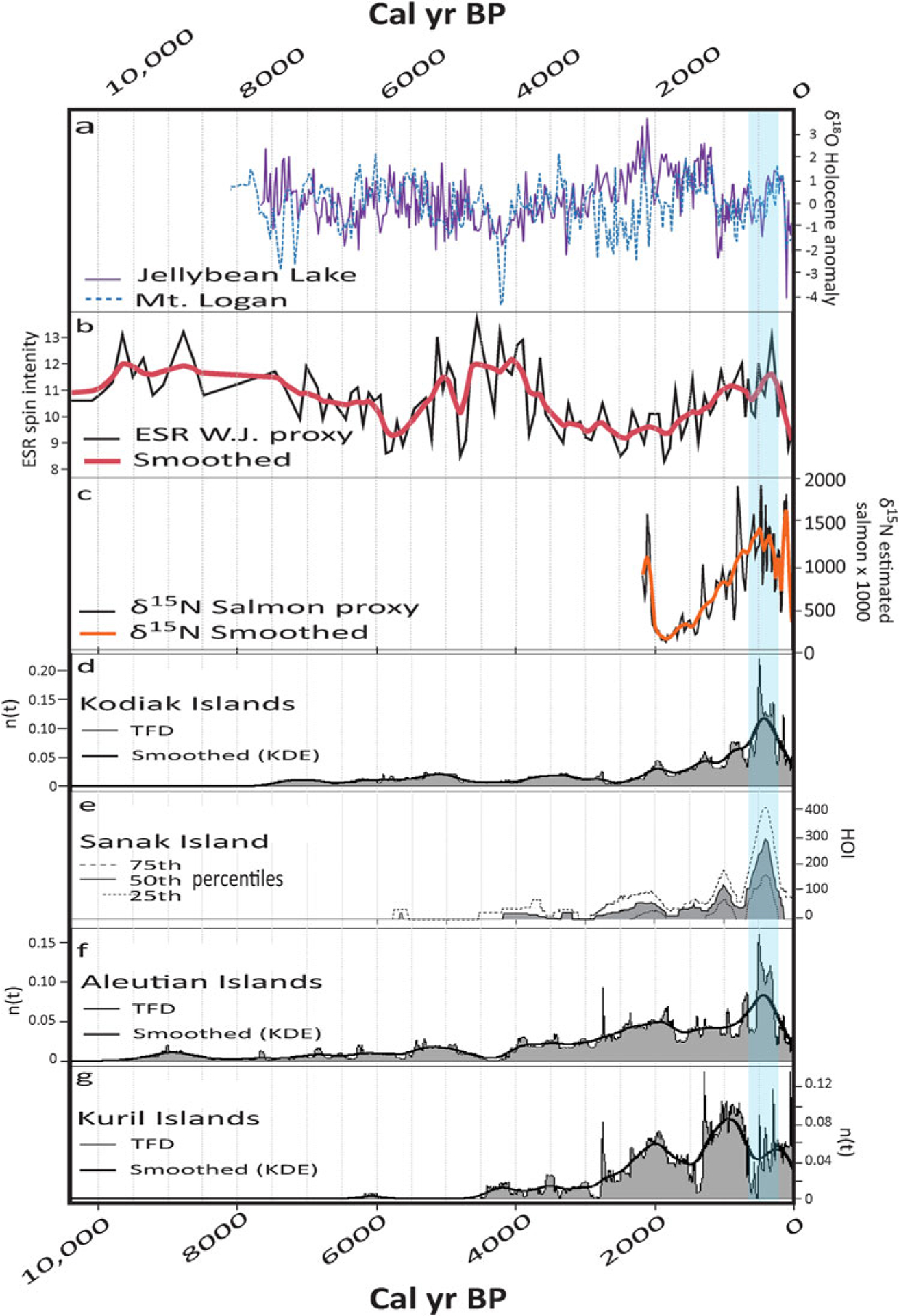
(a) Oxygen isotope record from NE Pacific (ice and lake cores): precipitation and Aleutian Low proxies (JBL from Anderson et al., 2005; MTL from Fisher et al. 2008). (b) ESR dates from a Sea of Japan marine core: reflecting shifts of the East Asian jet stream and related to AL strength and direction (Nagashima et al. 2013). (c) δ^15^N from Karluk Lake sediment core: proxy for sockeye salmon spawning population (Finney et al. 2002). (d-g) Archaeological radiocarbon temporal frequency distributions (TFDs), not taphonomically corrected (compare to Fig. 4), but cleaned to reduce sampling bias (Fitzhugh et al., 2020; Maschner et al., 2009a). See Table 1 for sample sizes and text for discussion.

Figure 3 shows long-term trends in proxies of climate, ecology and human populations based on marine, glacial, and lake cores, as well as archaeological radiocarbon accumulations. The atmospheric proxies depict century- to millennial-scale modalities in the North Pacific circulation from both sides of the North Pacific basin (Fig. 3a and 3b). These changes appear to correspond with late Holocene proxy Pacific salmon abundance trends from Kodiak Island (Fig. 3c; Finney et al., 2002) and human populations estimates (Fig. 3d; Mann et al., 1998; Fitzhugh et al., 2016, 2020), which also appear to have fluctuated at century and millennial scales around the North Pacific Rim. Together, these data are consistent with the possibility that human populations were sensitive to fluctuations in climate and food availability. They also suggest that millennial-scale variability in marine resources has significantly higher amplitudes than 20th century decadal shifts would imply (Hare et al. 1999; Finney et al. 2002; see also Maschner et al., 2009b).

If the trends in archaeological proxy populations are substantially influenced by bottom-up constraints on food availability, the spatial asymmetry between the Alaskan and Kuril data series then implies inverse spatial structuring of the ecological conditions between the east vs. west sides of the North Pacific (Fig. 3; Fitzhugh et al., 2020). At least for the Gulf of Alaska and Aleutians, these patterns also imply that the mechanisms forcing the marine ecosystems can be synchronous across large regions. Based on contemporary understandings of North Pacific climate variability, the intensity and position of the Aleutian Low (Rodionov et al., 2007)—or some similarly manifested climate pattern—underly this oscillating pattern. In other words, local and regional near-shore ecosystems may respond to the spatially structured forcing of coupled atmospheric-oceanographic processes that affect the entire North Pacific Rim systematically, but not uniformly, at intervals of centuries to millennia. In Fitzhugh et al. (2020), we called this the Long Interval Oscillations in the North Pacific (the “LIONPac model”).

### Evaluating the Possibility of Century to Millennial Scale Oscillations in Marine Ecodynamics and Paleodemography

The LIONPac model proposes the existence of multi-century to millennial-scale fluctuations in human populations caused by historically undocumented regime shifts in marine ecosystem. While unprecedented, researchers have only recently started to develop the techniques and accumulate the data to assess variability over such scales. Ultimately, evaluating this proposal will require coordinated interdisciplinary research to compile temporally resolved paleoecological proxy records (paleoclimate, paleoceanography, paleoecology, zooarchaeology, isotopic biochemistry, paleogenetics and archaeology) from around the North Pacific. Those data are not yet available at necessary spatial grain and temporal resolution, though we include preliminary comparisons of available evidence in what follows. A more immediately achievable approach is to validate the archaeological paleodemographic models over larger geographic regions to identify the scale(s) of coherence in the trends between regions.

Collaborations, initiated through the Paleoecology of Subarctic and Arctic Seas (PESAS) working group that inspired this special issue, have started assembling relevant evidence (see West et al., 2020 and Nagashima et al., 2021, in this issue). This coordinated research program promises to open new understanding of the paleoecology of the North Pacific and to extend the dynamic baselines available to contemporary ecologists, managers, and planners. Much more work like this is needed before we can evaluate the ecological predictions of the LIONPac model or replace it with any alternative.

The data series that inspired the initial formulation of the model were limited to just three areas from the Northeast Pacific and one from the Northwest Pacific (Fig. 3d). While the Northeast patterns were suggestive, it is easy to imagine how the correlations could be incidental, and the lack of any adjacent data for the Kurils made it impossible to assess the robustness of the Northwest pattern. Here we present a significantly larger number of regional time series, assumed to represent relative population variation, and we evaluate a set of hypotheses derived from the LIONPac model. We argue that spatial coherence in proxy population time series can be used to strengthen interpretations about large-scale demographic processes and their potential causes (e.g., Crema et al., 2016; Tremayne and Winterhalder, 2017; Tremayne and Brown, 2017).

Here we outline the hypotheses that frame our examination of the population models, and in the next section we examine the assumptions underlying the use of radiocarbon-based archaeological TFDs (temporal frequency distributions) as proxy population models. Where TFDs can be taken primarily as representations of population change through time, we hypothesize the following:

**Hypothesis 1 (Adjacency).** *Irrespective of any larger scale oscillations, populations living in adjacent (contiguous) regions should have more correlated population trends than populations living far apart*. This is because they are more likely to share homologous demographic, cultural, and ecological contexts.

C1. Large-scale environmental or cultural changes that effect the productivity of ecosystems, economies, human health and wellbeing across adjacent regions should lead to positively correlated human populations trends through feedbacks on fertility, mortality, in-migration, and out-migration. These changes could include widespread ecological regime shifts, adoption of common technological innovations, or the spread of epidemic diseases or occurrence of catastrophic events (e.g., earthquakes, volcanic eruptions, tsunamis). Net in-migration from distant regions may not be linked to local causes, but the larger the scale of movement the more likely that adjacent regions will share the resulting growth trend. Such migration (in or out) might be related to shifts in the spatial distribution of productive ecosystems, the development/adoption of technologies that open new ecological niches to resident or immigrant communities, or access to or victimization by new methods of warfare.

C2. More local-scale environmental and cultural changes will more often result in inversely correlated population trends, for example when people leave one region in favor of an adjacent one. The scale of analysis matters in interpreting these patterns.

C3. Population movements that do not cross the boundaries of geographical units of analysis will not alter net population trends. Until recently, communities living primarily by hunting and collecting wild foods moved logistically if not residentially, and with some frequency—with longer moves less common than shorter ones. Different scales of spatial analyses then should capture different dynamics. Smaller spatial units should capture more frequent and less exceptional patterns of population movement, and population fluctuations in adjacent areas are more likely to be related. Larger spatial units, by contrast, should predominantly reflect endemic population variability, with transborder migration impacting trends only under the more extreme cases of large-scale migration events.

Unfortunately, the larger the scale of analysis, the more internal variability will be averaged in net population trends (see Timpson et al., 2014). This averaging reduces analytical sensitivity in spatial and temporal dimensions, while preserving dominant trends, but also sometimes accentuating unrelated but synchronous internal dynamics that may not be individually significant (i.e., causally unrelated but correlated patterns in different parts of the analytical region).

Expectations: C1 should be observable in positively correlated trends (up or down) in demographic (TFD) evidence. C2 should be detected in inversely correlated TFD trends in adjacent series. C3 simply acknowledges that there is a spatial scale dependency to population histories that will determine the sensitivity of the analysis and potential for interpreting observed patterns. Neutral growth in a portion of a time series does not imply that demographically meaningful events did not affect populations within the regions, only that they are not detectable at the resolution available.

**Hypothesis 2.** *Regardless of adjacency, if populations are density dependent or otherwise tuned to the unique ecological conditions in which they live, then population trends should correlate positively in regions sharing similar ecological/environmental characteristics/histories more than across ecologically differentiated regions*. This hypothesis supports the assumption that marine ecosystems across the North Pacific study area respond to synchronous environmental/climatic forces, and that different kinds of ecosystems (e.g., ocean vs. terrestrial) respond differently to the same forces. This assumption is generally reasonable where communities of hunting and gathering people lived on marine or terrestrial animals rooted in the same food webs and influenced by the same bottom-up ecological factors.

Regions where people made their living primarily from the same ecosystem resources should have more similar population trends than regions of different ecotypes. For the purposes of these analyses, we define ecotype into marine (coastal) and terrestrial with the terrestrial divided into forested (boreal) and tundra zones. Boreal zones are found in the interiors of both Hokkaido and Alaska, while inland tundra zones are only found in regions of Alaska. Because the tundra zones also happen to be relatively close to the coasts, we expect them to potentially track coastal demographic features, so they constitute a class of ‘semi-coastal’ units when testing for coastal vs. interior relationships.

Expectations: Proxy population trends should track more closely between regions sharing the same ecological classification (coastal, tundra, or interior) than between them.

**Hypothesis 3.** *If century- to millennial-scale spatially structured oscillations in ecological productivity influenced the NE Pacific (Alaska) and NW Pacific (Hokkaido and Kurils) macro-regions in opposite ways, and if human population growth dynamics in the past were density dependent (i.e., food limited), then regional populations within each macro-region should be positively correlated and inversely correlated with the regional trends on the opposite side of the Pacific*.

H3a. If the forces driving these patterns are predominantly *marine* (e.g., related to regime shifts in anomalously cold and warm water temperatures linked to the spatial shifts in the Aleutian Low system, as proposed by the LIONPac model), then coastal population trends on the same side of the Pacific should exhibit strong positive correlation, terrestrial and coastal population trends within macro-regions should be uncorrelated, and coastal population trends should be inversely correlated on either side of the North Pacific.

H3b. If the forces driving these patterns are predominantly *terrestrial* (e.g., temperature-linked impacts on caribou and deer survival), then non-coastal populations on the same side of the Pacific should exhibit strong positive correlations, terrestrial and coastal populations within macro-regions should be uncorrelated, and terrestrial population trends should be inversely correlated with those across the ocean. Based on synthetic paleoecological reconstructions for Alaska’s interior regions showing highly asynchronous ecological variability over small distances (Kaufman et al., 2016), we do not expect this hypothesis to be supported.

H3c. If the forces driving macro-regional ecological productivity cross marine and terrestrial ecozones (e.g., due to the integrating ecological effects and subsistence importance of anadromous fish or the greater integration of coastal and interior human populations), then population macro-regional patterns should be positively correlated within macro-regions and inversely correlated between them, irrespective of coastal vs. terrestrial classification.

**Hypothesis 4.** *It is also possible that population dynamics only became linked across larger regions by the arrival of colonial economies, exploitative treatment of Indigenous people, and introduction of epidemic diseases. If that were the case, we would expect regional trends to remain uncorrelated (H1-H3 falsified prior to colonial contact) until the onset of significant colonial economic and political interaction and increasingly correlated (perhaps with some lag from earlier to later contacted areas) around the 7*^*th*^*-9*^*th*^ *century CE, while the Alaska series should become more correlated only in the 19th century CE*.

Entanglement in expanding “world system” economies (Chase-Dunn and Grimes, 2002; Hudson, 2004) could have had positive and negative effects on population dynamics. Pursuit of resources for exchange and of trade partners with whom to trade could have stimulated migrations, social competition, and wealth (for successful actors) that might support population expansion. At the same time, aggressive competition could have led to excess mortality, balancing out gains in endemic population expansion, and expanding trade networks would have also supported transmission of pathogens with potentially significant impacts on previously more insular populations (Fitzhugh et al., 2011). Epidemic diseases are known to recur periodically in vulnerable populations until endemicity is established, which occurs more rapidly in dense and highly connected (e.g., urbanized) populations (Suzuki, 2011). Smallpox was one of the first major diseases of colonial expansion into North Pacific communities. The disease hit central Japan by the 8^th^ century CE (Behbehani, 1983) and could have reached Hokkaido through trade interactions soon after. Hokkaido and Kuril populations could have been impacted by multiple epidemics over the centuries of the low-level contact that prevailed from the late first millennium to mid second millennium CE (Hudson, 1999, 206–232). Russian-American conquest brought diseases first to the Gulf of Alaska region in the early 19^th^ century and later up the coast into central and northwest Alaska in the mid to late 19^th^ century (Fortuine, 1989). Demographic impacts should then have been seen first on the south coast, then north coast, and lastly in the terrestrial interior.

In the Alaskan case, significant population growth is not anticipated (the numbers of Russian-American period colonizers were always very small relative to the Native populations), and the primary signal should be decline, based on historical documentation of the process (e.g., Lührmann, 2008). Recent research has significantly undermined prior claims that virgin soil epidemics spread faster than the arrival of the colonial disease-bearers themselves (see Liebmann et al., 2016; Feinman and Drake, 2021). If correct, then it is unlikely that synchronized population collapse in Alaska prior to Russian contact could be attributed to indirect transmission of diseases brought by European colonial incursions into eastern and southern North America and Mexico in the 16^th^ and 17^th^ centuries.

## TFD GENERATION: METHODS AND CONSTRAINTS

Estimating human population history is both an essential element to understanding long-term human-environmental dynamics and one of the more difficult tasks in archaeology (Kelly, 2018). Throughout the late 20^th^ and early 21^st^ centuries, one way that archaeologists have approached this problem is through the analysis of temporal frequency distributions (TFDs), statistical constructs that describe or model temporal changes in counts of dated artifacts, burials, houses, rooms, sites, or site areas within bounded study regions. The central tenet of this approach to paleodemography is the proposition that changing rates in the creation and deposition of cultural material tracks changes in population abundance. TFDs in this case reflect trends in regional changes in net population abundance.

Even so, the shapes of TFDs are inevitably influenced by other factors alongside changing population abundance, not only by the interests of the researchers who selected the original samples for dating (*sampling bias*), but also by underlying issues of archaeological visibility and preservation (what remains to be sampled; *taphonomic bias*), as well as changes in the per capita rate of creation/deposition of cultural materials resulting from changing settlement organization and land use patterns (*creation bias*). In combination, these non-demographic influences on TFDs amplify, mute, or overwhelm demographic signals represented in TFD trends. In addition, all else being equal, the distributional structures of TFDs based on small samples will provide poor approximations of their underlying probability distributions due to random sampling error. Consequently, while unique TFD trajectories should contain demographic information, they provide no key for disentangling their demographic relevance from the other processes they reflect. TFDs are best consulted as proxy census records when cultural processes, visibility and preservation issues, and research histories can either be ruled out or mitigated. More pragmatically, TFDs serve as *hypothetical paleodemographic models*, and their interpretations should include efforts to identify non-demographic influences.

Over the last two decades, archaeologists have invested considerable time, resources, and effort in aggregating large radiocarbon databases, allowing us to better address the problem of random sampling error (Rick, 1987; Williams, 2012; Brown, 2015, 2017; Fitzhugh et al., 2016; Crema et al., 2016, 2017; Robinson et al., 2019; Kelly et al., 2021; Price et al., 2021; Shennan and Sear, 2021; Crema, 2022). These efforts to aggregate and archive data in digital repositories and targeted attempts to compile archaeological radiocarbon databases at up-to-continental scales (e.g., Canadian Archaeological Radiocarbon Database /CARD: https://www.canadianarchaeology.ca; Kelly et al., 2021; Palmisano et al., 2022) have made it possible to compile TFDs of dates for many regions of interest. The present study benefits from the availability of such radiocarbon databases, drawing on large samples of radiocarbon data from Alaska, Hokkaido, and the Kuril Archipelago.

However, constructing TFDs for meaningfully bounded study areas inevitably necessitates the partitioning of these larger radiocarbon data sets into smaller subsamples. In some cases, such division entails prohibitive reductions in sample size, compelling us to favor minimal analytical units larger than meaningful demographic divisions, especially in Alaska. Inevitably, any degree of aggregation will average the signals of localized TFDs within the boundaries of the study area. While there is no upper limit on the size of the analytical unit, we seek a balance between aggregates large enough to satisfyingly mitigate random sampling error and aggregates that do not wholly wash out or entangle disparate demographically meaningful TFDs. In this case, we balanced the sample density per unit area (denser in Hokkaido) and the desire to distinguish coastal and interior sampling areas from each other. This led to larger Alaskan analytical units and smaller Hokkaido ones. Table 1 reports the numbers of dates included in each regional set of our study, as well as estimates of effective sample size after penalizing redundant dates from the same locations in a manner discussed in Supplemental Materials.

Increasing sample size is not the only means of reducing random sampling error. To further mitigate this problem, we also apply kernel density estimation, a widely used nonparametric statistical technique intended to smooth away idiosyncratic but random peak and valley structures in sample distributions while preserving distributional structures original to the underlying probability distribution. More specifically, we introduce and apply a novel protocol—composite redundancy filtering through presence-absence buffering (CRFPAB)—which contributes a new member to the broad family of composite kernel density estimation discussed under different names by Baxter and Cool (2016), Bronk Ramsey (2017), Brown (2017), and McLaughlin (2019). Technical details of this protocol are provided in Supplemental Materials, but in brief it attempts to simultaneously mitigate random sampling error, chronometric uncertainty, and one form of sampling bias: chronometric overrepresentation of site occupation episodes resulting from intensive intra-site sampling.

To mitigate the problem of taphonomic bias, we apply a deterministic correction factor to our regional TFDs, defined as the inverse function of the taphonomic survival function introduced by Surovell and colleagues (2009). This is a departure from previous TFD analyses by several of the coauthors (Fitzhugh et al., 2016, 2020; Gjesfjeld et al., 2019). This ‘correction’ is approximate, necessarily inflating features at the older end of the time series where samples sizes are typically smaller, and its accuracy will vary between geographical regions with different underlying rates of deposition, erosion, and degradation (see Supplemental Materials for technical details of this correction, as well as graphical comparisons of corrected and uncorrected series; cf. Williams, 2012, fig. 7 and text). Despite these caveats, the taphonomic adjustment enables readers to view the TFD curves without undue attention to aggregate positive trends, drawing focus instead to the deviations from those trends, which is where our primary interests lie.

Ruling out non-demographic cultural influences on the accumulation of cultural material requires consideration of the archaeological evidence associated with the TFD data sources themselves. We will address cultural considerations when reviewing the regional series below.

While large aggregations of radiocarbon data may greatly reduce the problem of random sampling error, in many cases—especially when aggregated data come from numerous prior research projects with varying degrees of documentation—it is not possible to rule out all of the various forms of bias, especially sampling bias. In these cases, paleodemographic researchers often make the working assumption that, with large enough data sets drawn from numerous independent sources, the idiosyncrasies of the original research projects will be statistically neutralized (Rick, 1987). This assumption makes many archaeologists nervous and has led to a critical countercurrent (Contreras and Meadows, 2014; Attenbrow and Hiscock, 2015; Hiscock and Attenbrow, 2016; Carleton and Groucutt, 2020).

In this study, our approach to mitigating this skepticism is comparative. While we accept that TFDs are noisy and affected by multiple non-demographic influences that cannot always be ruled out or mitigated through the application of targeted analytical procedures, we look for interregional structural coherence between the temporal features of multiple regional TFDs. In principle, statistically significant trend correlations between neighboring series could be taken as a strong sign of an underlying process worth further investigation. Comparison of multiple TFD time series therefore provides a method to identify coherence between regions, while retaining the spatial discrimination that would be lost by pooling the samples into larger regional analytical units than the ones we have defined. The greater the coherence in data structure between series (i.e., the more correlated the trends), the more likely those trends are demographically meaningful at scales larger than individual series and not driven by idiosyncratic research histories or local preservation factors (see Contreras and Meadows, 2014; Brown, 2017).

The comparative approach applied in this study takes two forms. First, we conduct visual comparison of TFD time series constructed for 14 study areas across the Hokkaido-Kurils and Alaskan data sets. This approach allows us first to see the structure of the individual regional trends and to compare these to other lines of archaeological evidence, and then to consider the inter-regional relationships between these trends. Approaching the series in this way also serves a ‘ground-truthing’ role, allowing readers to judge for themselves the integrity of the interpretations proposed. Nevertheless, the sheer number of trends and their inherent noisiness calls out for some method or mechanized procedure to evaluate the relatedness of multiple trends.

We thus introduce a novel statistical method involving the time iterative analysis of interregional correlations between TFD structures. This method uses the Moran’s *I* statistic (Moran, 1950) to summarize spatial and ecological autocorrelations between the growth rate time series derived from 13 regional TFDs (excluding the unique Tsugaru Strait data series). Technical details for this time iterative Moran’s *I* (TIMI) analysis are provided in Supplemental Materials. We believe the application of this method allows us to identify inter-regional correlations that can be attributed to demographic changes used to evaluate the hypotheses above.

The interregional comparison of TFDs is a relatively straightforward procedure insofar as each TFD shares a common X axis—an absolute calendar timeline—in contrast to the age model interpolations, axis adjustments, and curve fitting that are often required when comparing proxy paleoenvironmental records. This does not mean that radiocarbon TFD series are necessarily accurate representations of the archaeological events they are taken to represent. TFD time series based on radiocarbon dates are constrained by the same issues affecting radiocarbon dates in other contexts.

Where terrestrial charcoal and other wood are used exclusively for building the TFDs (as in this analysis), the most likely issue to affect the trends the inclusion significant numbers of “old wood” dates in TFD data sets. Like most other radiocarbon paleodemographic analyses, here we assume that old wood issues do not systematically bias our results. That working assumption deserves scrutiny, however, especially for data sets from places like the Arctic where deadwood can preserve for centuries after death or in places where long-lived trees could have been used as fuel and raw materials (McGhee and Tuck, 1976; Anderson and Feathers, 2019; Ledger and Forbes, 2019). Collectively, large samples of dates should reflect the ages of the pooled life-histories of the underlying source forests, not specifically the antiquity of tree deaths, however closely associated those tree deaths were to the human use of the wood (i.e., the target date). We must assume for now that, on average, prehistoric fire-builders disproportionately used fuels from recently dead trees and shrubs because 1) prehistoric use of long-dead wood should be rare relative to use of recently dead wood in any assemblage, 2) geometrically, the volume—and therefore chance of selection for dating—of outer rings/later-growth wood (closer to the date of tree death) should be greater than the volume and chance of sampling earlier growth from the dead tree, 3) processing large logs or trees for domestic fires (the assumed source of most wood sampled here) would have been inconvenient compared to the use of smaller branches with age profiles closer to the death of the tree (or branch) and 4) common tree taxa around the North Pacific Rim live an average of only 100–200 years (Fitzhugh et al., 2016). Accordingly, the preponderance of dates in any large data set should derive from wood that predominantly dates close to the target event (human activity). The resulting one-sided skew expected from the inclusion of some amount of ‘old-wood’ dates, randomly distributed through large data sets, should have the main effect of dampening amplitude variability in trends by pulling some of the TFD mass in the older direction, but the inclusion of such dates should not seriously affect the location of trend inflections.

## RESULTS OF TFD ANALYSES

### Visual interpretation of Proxy Population Curves

**Figure 4.**
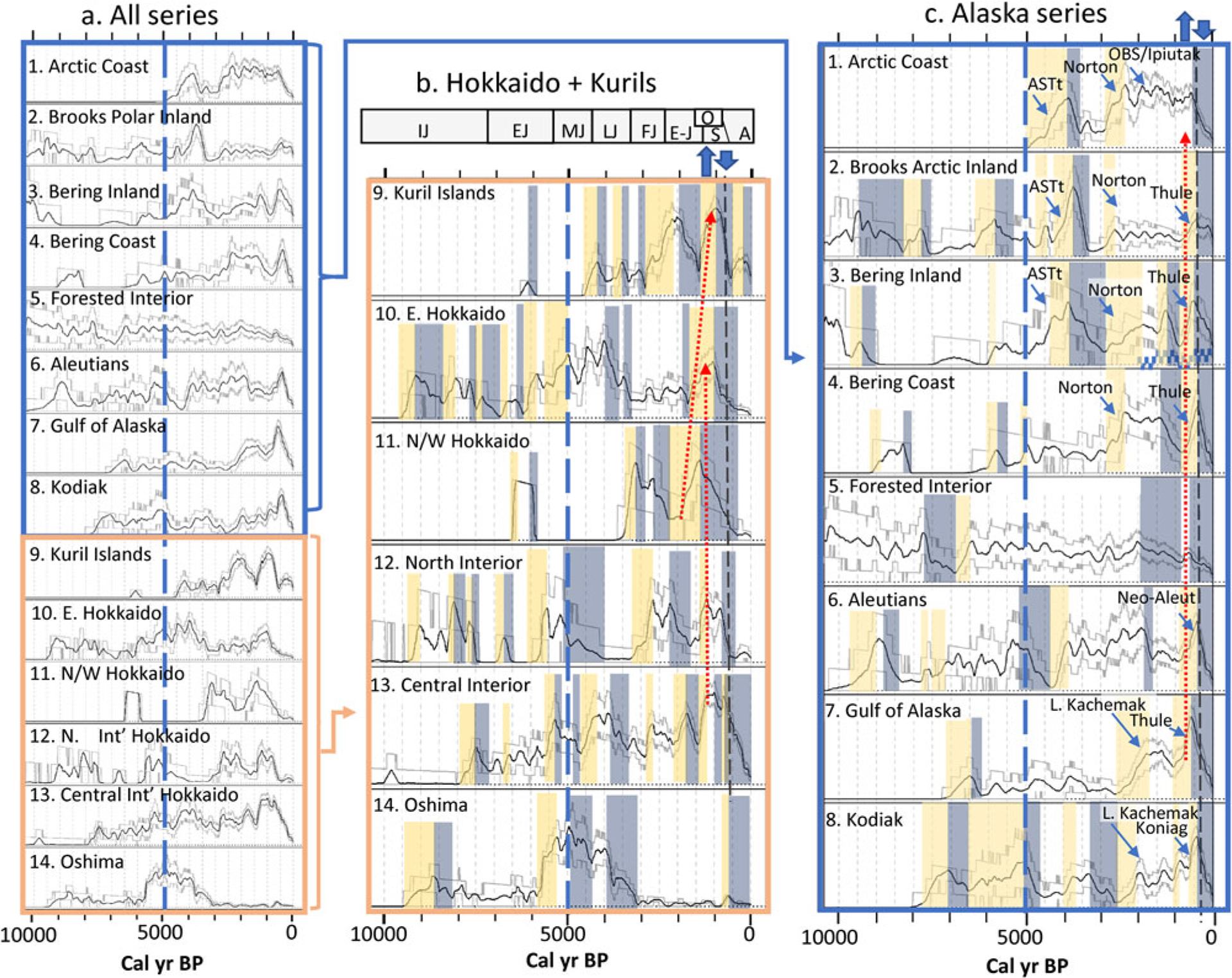
Stacked, taphonomically ‘corrected’ TFD graphs of archaeological radiocarbon dates from regions around Alaska, Hokkaido and the Kuril Islands presented as proxy paleodemography models (see text for explanation and interpretation). Dark central trend lines are smoothed central tendency representations of the underlying data using the CRFPAB method (see Supplement). 95% confidence interval envelopes are shown as lighter grey upper and lower bounds. The left panel (4a) includes all regional data series; the central (4b) and right (4c) panels present greater detail of the Hokkaido+Kuril and Alaska series, respectively. Yellow and grey bars are manually placed highlights to show areas of notable trend increase and decrease, respectively. These highlights are placed wherever either the ending min-max envelope has moved out of the range of the starting min-max envelope (making a neutral growth interpretation unlikely) or where the central trend line at least doubles or halves during the excursion. Blue dashed lines mark 5000 yr, which is the approximate cut-off for visual analysis and absolute cut off for the TIMI spatial autocorrelation (earlier trends are insufficiently robust for comparison). The purple dashed arrow in panel 4b illustrates the time transgressive shift from North Hokkaido to the Kurils thought to track expansion of the Okhotsk Culture. The red dotted arrows on Figure 4b and 4c demarcate the largely synchronous increase in populations, starting ca. 1300 yr in Hokkaido and after ca. 750 yr in all Alaska. The narrow black dashed lines in the central (4b) and right (4c) panels depict the correlated population collapses indicated for Hokkaido/Kurils ca. 800–500 yr and in Alaska ca. 400 yr and continuing through colonial contact. Blue block arrows on the top right corners of 4b and 4c point to major late Holocene TFD growth and collapse trends across multiple time series for the Hokkaido/Kuril and Alaska data sets, respectively. These trends are negatively correlated in time between the two macro-regions (see text for discussion). The gray strip across the top of 4b delineates Hokkaido culture historical phases: IJ = Initial Jomon, EJ = Early Jomon, MJ = Middle Jomon, LJ = Late Jomon, FJ = Final Jomon, E-J = Epi-Jomon, O = Okhotsk, S = Satsumon, A- Ainu. Culture historical designations of relevance are shown on the curves. See Figure 2 for a more complete set of culture historical schemes.

Figure 4 shows the archaeological radiocarbon TFDs for the last 10,000 years for 14 regional units across the North Pacific, including five from Hokkaido and one from the Kuril Islands (Fig. 4b) and eight from Alaska (Fig 4c) (see Figure 1 for geographical boundaries). The regions are defined as spatially contiguous zones, divided into coastal, near-coastal tundra (for Alaska), and interior boreal forest eco-regions to distinguish between regions supporting maritime vs. terrestrially dominated subsistence opportunities. Other than the Kuril Islands, we do not attempt to present data from the Russian Far East or Northeast Siberia because research histories and the resulting radiocarbon datasets for these regions are too small and fragmentary to produce meaningful TFDs at suitable spatial scales (Kuzmin, 2010).

For visual inspection, the 14 series in Figure 4 are arrayed in approximate geographical proximity and stacked as a whole set (Fig. 4a), as a Northwest Pacific stack (Hokkaido + Kuril Islands; Figure 4b), and as a Northeast Pacific stack (“Alaska Series,” from the Arctic coast to Kodiak; Figure 4c). Each trend plot includes a 95% confidence envelope, generated by Monte Carlo simulations, that better represents the changing effect of sample density through time in each series (see Supplemental Materials).

Visual inspection of Figure 4 allows comparison of trends between adjacent series. Notably, each series starts at a different time depth due to the uneven antiquity of archaeological evidence in each region (here, all series are truncated at 10 ka), and while some differences in the early part of the time series may be archaeologically meaningful, they render many TFD comparisons problematic in the early Holocene (and for some comparisons, prior to 2500 yr). As a result, we begin the visual analyses in the middle Holocene when archaeological data becomes available from the Bering and Arctic coasts of Alaska (and Siberia) (Fitzhugh, 2016; Tremayne and Brown, 2017).

Before considering any subset of curves, we note that all curves can be decomposed into at least two scales of periodicity, which we will call long-period (>1000 yr) and short-period (<1000 yr) variability). Long-period variability is generally characterized by larger fluxes and enduring modes or regimes, while the transitions between them can be abrupt, often within 100–200 years. Short period variability is manifest as fine-scale fluctuations that reverse within a few hundred years. These short-period “sawtooth” fluxes have relatively low amplitudes and, for the most part, vary without transgressing the 95% confidence interval windows. This short-period variability cannot be distinguished from a null model of neutral growth and so is potentially meaningless in depicting population change. Even shorter transitions may have occurred that are obscured by the kernel density smoothing function used. Notable short- and long-period positive and negative trends are depicted in Figures 4b and 4c with colored shading (see Fig. 4 caption for explanation).

### Northwest Pacific Region (Hokkaido and Kuril Islands)

#### Culture Historical Framing.

The Island of Hokkaido and the Kuril Islands that extend to the northeast from its eastern end share basic archaeological characteristics with the larger Japanese Archipelago, with some significant differences. In brief, the adoption of pottery-making around 16,000 yr marks a transition from relatively mobile Paleolithic lifeways to more localized, increasingly sedentary and intensive hunting-fishing-gathering cultures of the Jomon Period (Ikawa-Smith, 2022; Jordan et al., 2022). Broken into several subphases, the Jomon period continued for more than 10,000 years, marked by increasingly sedentary lifestyles and more intensive terrestrial and marine hunting, fishing and gathering economies (Habu, 2004; Matsumoto et al., 2017). In the first millennium BCE, the Jomon hunting and gathering lifestyle was transformed by rice agriculture in the Yayoi tradition in western and central Japan but persisted in what comes to be called the Epi-Jomon in Hokkaido and the Kurils. Epi-Jomon people continued to hunt, fish and gatherer wild terrestrial and marine foods, with increasingly intensive fishing strategies (Takase, 2014, 2020a).

Between the second and fourth century CE, Epi-Jomon communities near the northern tip of Hokkaido and on the small off-shore islands to its west started interacting more frequently with communities in southern Sakhalin Island, a process that may have resulted in the formation of a new cultural hybrid, the Okhotsk on both sides of Soya Strait. The Okhotsk became intensive sea mammal hunters and traders in a growing commodities trade, fueled in part by elite markets in central Japan and mainland Northeast Asia. They lived in large pithouse villages, often in substantial dwellings suitable for extended family groups (Ohyi, 1975; Amano, 1979; Ono, 2008). They were soon traveling down the west coast of Hokkaido as far as Okushiri Island where they interacted with Epi-Jomon/Satsumon people from southern Hokkaido and possibly northern Honshu (Masuda et al., 2001; Matsumura et al., 2006). In the 6^th^ and 7^th^ centuries CE, the Okhotsk expanded east along the Sea of Okhotsk coast and up the Kuril Island chain. Elsewhere in Hokkaido, the Epi-Jomon adopted horticultural practices from Honshu, leading them to be recognized archaeologically as a new culture, the more terrestrially Satsumon culture. In some combination, the Okhotsk and Satsumon gave rise to the Ainu, the Indigenous people of northern Hokkaido today, and—until the end of World War II—in southern Sakhalin, the Kurils and southern Kamchatka (Sato et al., 2009; Takase 2018). Building on trends pioneered by their predecessors, the Ainu became increasingly entangled in the East Asian commodities trade, as producers of wild products and active intermediaries in trade between the wealthy states of China and Manchuria on the one hand and Japan on the other (Hudson, 1999; Walker, 2001; Tezuka, 2009).

Except for the northern most islands adjacent to Kamchatka, the Kurils share the same basic culture historical framework as Hokkaido. They saw at least three intervals of expanded settlement followed by collapse, corresponding to the Late Jomon/Epi-Jomon phase, the Okhotsk and then the Ainu phase (Fitzhugh et al., 2016; Fitzhugh, 2019; Takase, 2020b). We will return to these population fluctuations more quantitatively, below.

#### Northwest Pacific TFD Paleodemography.

In comparison with the Alaska series discussed below, the Hokkaido + Kuril trends are considerably less synchronous in long-period variability. Nevertheless, shared patterns are observed that fit the general archaeological understanding of the area. In many of the regions, populations were generally growing from 6000 to 5000 yr, particularly around the Oshima coast (#14), E. Hokkaido coast (#10), and, to a lesser extent, in the Central Interior (#13) and North Interior (#12). This interval ushered in the Early and Middle Jomon phases and our data is consistent with dramatic population growth recognized previously in Hokkaido as well as across the northeastern half of Honshu (Crema et al., 2016; Koyama, 1978). Middle Jomon expansion lagged in the Kurils (#9) until around 4500 yr (when we date the earliest settlement of the more remote and smaller central islands: Fitzhugh et al., 2016). In contrast, growth is not seen at this time on the North and West Hokkaido coasts (#11) though this series has a uniquely small sample size and is not considered reliable prior to about 3000 yr (Table 1, Figure 4). Populations appear to go into decline around Hokkaido as they do in Honshu during the Late Jomon phase: in the North Hokkaido Interior (#12) about 5000 yr, in Kanto (central Honshu) ca. 4400 yr (Crema et al., 2016), and closer to 4000 yr in Amori (northern Tohoku; *ibid*), the Oshima Peninsula (#14), and Eastern Hokkaido (#10). Except for the Middle Jomon interval, the Oshima Peninsula is exceptional for its uniquely low archaeological representation through the later Holocene. This anomaly deserves additional scrutiny by scholars of Hokkaido archaeology.

Hokkaido’s Central Interior data set (#13) stands out for its overall lack of the long period/large amplitude variability seen in most other data sets of Hokkaido and the Kurils, a pattern replicated as well in Alaska’s Forested Interior (#5). Data set #13 spans the Ishkari Plain between modern day Sapporo and Chitose and could be interpreted as the most stable of the Northwest Pacific regions under consideration. Like the Tanana corridor in Alaska (within #5), the Ishkari Plain is the region with the highest concentration of archaeological research and hence the largest sample size (by large margin) than any other Hokkaido (or Alaska) region. This opens the possibility that differences in sampling intensity play a role in the differences between Hokkaido assemblages. On the other hand, the use of 95% confidence intervals (CI) to identify significant deviations in all series allows us to evaluate small sample bias through time in each series, and all have very significant fluctuations in intervals of tight Cis after 5000 yr, except for the two northern Hokkaido sets (#11 & #12) that become meaningful ca. 3500 yr.

Whereas southern, central and eastern Hokkaido were fairly continuously occupied in the mid-Holocene, northern Hokkaido and the Sea of Japan coasts (Fig. 4, #11) and north interior regions (#12) appear to have been sparsely populated prior to 3500 yr. Despite this, archaeological settlement dated by diagnostic pottery suggests a significant increase in the number of archaeological sites of Middle Jomon phase both on the coast and interior of northern Hokkaido (Abe et al., 2016). This pattern shows up as a spike in trend #12 (North Interior) but is missing completely in #11 (on the North/West Coast).

The material culture affiliations link southern and central Kuril occupations through the mid and late Holocene exclusively to cultures in Hokkaido (Tezuka and Fitzhugh, 2004; Fitzhugh et al., 2016). Kamchatka residents did settle the northernmost islands of Shumshu and Paramushir, eventually meeting and integrating with cultures from the south as early as Epi-Jomon times (Takase et al., 2017). The Kuril curve (Fig. 4, #9) suggests that Jomon populations persisted through a series of low-level fluctuations from 4500 to 2400 yr (from the end of the Middle Jomon and through the Late and Final Jomon phases), while regions in Hokkaido saw larger scale declines over this interval. From that base, the Kurils then saw explosive growth during the Epi-Jomon from 2400–2000 yr, likely as a result of substantial in-migration from Hokkaido (Fitzhugh et al., 2016). This migration may have emanated out of the Eastern Hokkaido coastal region, which was amply populated and adjacent to the entryway to the Kurils.

The Final Jomon and Epi-Jomon population boom in the Kurils after 3000 yr is balanced to some extent by negative populations trends in the North/West Hokkaido coast (#11) and the North Interior (#12). A decline in late Epi-Jomon sites across Hokkaido in the early 1st millennium CE has been previously recognized and interpreted as an increase in residential mobility (Ishii, 1997; Takase, 2014). Southern Sakhalin was also settled by migrants from Hokkaido in this same interval suggesting that opportunity or necessity drew Jomon/Epi-Jomon people from northern Hokkaido up both sides of the Sea of Okhotsk at this time (Vasilevski, 2003).

The collapse of the Epi-Jomon in the Kurils from 2000 to 1400 yr helped inspire the LIONPac model. The other Hokkaido data sets do not share the Kuril trend in the aggregate. While East Hokkaido (adjacent to the Kurils) also declines briefly at 2000 yr, the trend reverses a few hundred years later while the Kurils continues to decline. In fact, the only other series from Hokkaido that matches the Kuril Epi-Jomon collapse is the non-adjacent and presumably unrelated inland Central Interior (#13). As we will argue this does not rule out the LIONPac-model, but it does underline the point that complex variables in addition to large-scale ecological regime shifts were in play in Hokkaido during the first millennium CE. Trans-regional migrations were clearly part of the story as increasingly were inter-regional, political-economic forces affecting the East Asian ‘world system’ (Hudson, 2004).

The Okhotsk expansion is exemplified by a ‘traveling mode’ in growth and decline starting earliest (ca. 1700 yr) in the North Okhotsk/Sea of Japan coastal series (#11). This growth subsequently shifts to the eastern Hokkaido coastal series (#10) and then moves to the Kurils (#9). This mode fits the archaeologically described growth and expansion of the Okhotsk cultural complex (Ono, 2008; Fitzhugh et al., 2016). While consistent with an interpretation of migration, these offset modes do not disprove the LIONPac model—migrations may have been fueled by changes in resource availability/productivity from the Sea of Japan to the Pacific sides of Hokkaido—but they do remind us that the mechanisms of ecological change would not have been spatially uniform even across relatively short distances. Japanese archaeologists have noted that the Okhotsk migration co-occurred with a shift in subsistence focus from predominantly fish-based diets in the Sea of Japan and Soya Straits to more marine mammal-based diets in the Sea of Okhotsk (Amano, 1979; Yamaura, 1998). These discrepancies may also reveal inconsistencies in the middle Okhotsk chronologies.

### Northeast Pacific Regional Analyses (Alaska)

#### Culture Historical Framing.

The Alaska region is considerably larger than Hokkaido and the Kurils. As a result, the archaeological history of the region cannot be summarized as simply. The Gulf of Alaska, the Bering Sea, the North Slope/Arctic coast and Interior all have unique, though not unrelated, cultural sequences. The earliest archaeological evidence is currently concentrated in the boreal Interior along the Tanana River and northern foothills of the Alaska Range. The terminal Pleistocene archaeological record of Alaska has long been the focus of interest in connection with questions about the initial peopling of the Americas (Graf and Buvit, 2017; Potter et al., 2017).

Out of these earliest settlements, in combination with apparent influx of migrants from the Northern Plains at the Pleistocene-Holocene transition, terrestrial adaptations persist through the early, middle, and late Holocene (Holmes, 2008). One of the most profound changes to face this region was the establishment of the northern boreal forest, which moved into central Alaska in the early Holocene following a succession from steppe tundra to open parkland and eventually closed spruce/birch forests by approximately 6000 yr (Edwards et al., 2000). Holmes (2008) groups the cultural assemblages of the last 6000 years in the middle and late Taiga phases, in which the Northern Archaic centers in the middle phase and the introduction of the bow and arrow defines the late phase, which can be connected directly to northern Athabaskan ancestry. Subsistence throughout variously included resources such as moose, wood-land caribou, waterfowl, and anadromous fish.

Across south-central and southwest Alaska, the earliest coastal and near-coastal settlements between 8500 and 7000 yr share common technological features, in part similar to, and possibly derived from, the terminal Pleistocene traditions of the Interior (Jordan, 1992; Steffian et al., 2002). Early occupation on the coasts of the eastern Aleutians, Kodiak, and the mainland Alaska Peninsula involved hunting marine mammals with harpoons, fishing offshore with hook and line, and harvesting more or less the full complement of coastal and marine resources that would be used later, albeit less intensively and while living relatively mobile lifestyles (Fitzhugh, 2003; Kopperl, 2003; Schaaf, 2009). By around 5000 yr on Kodiak and the Lower Alaska Peninsula and by 4000 yr in the Aleutians, people started experimenting with net technologies (Jordan and Maschner, 2000; Fitzhugh, 2003; Davis et al., 2016). Net fishing had a fundamental effect on settlement and social life as people for the first time could produce enough food to store through the lean winter, encouraging the establishment of more permanent settlements and investment in more substantial dwellings. From 2500 yr and especially after 1000 yr, southwest and southcentral Alaskan communities grew to include substantial villages associated with smaller seasonal fishing camps. Both prestige contests and violent competition is evident in the use of artifacts of personal adornment, competitive games and feasting, large whale hunting, defensive sites and mortuary treatment (Fitzhugh, 2003; Davis et al., 2016; Steffian et al., 2016). The last phase in this history was initiated by Russian conquest in the late 18^th^ century, when conscription of Native hunters for sea otter harvesting, impacts of epidemic diseases and missionization transformed Indigenous communities across the region (Veltre and McCartney, 2002; Lührmann, 2008). The U.S. purchase of Alaska, followed by the explosion of commercial salmon, cod, crab, and other fisheries, further altered Native lifestyles in the late 19^th^ and 20^th^ centuries (Pullar, 1992).

North of the Alaska Peninsula, Alaskan coastal settlement is first detected, albeit indirectly, after about 5000 yr with the initiation of the Artic Small Tool tradition (ASTt) from Norton and Kotzebue Sounds. ASTt communities are thought to have lived rather mobile lifestyles. In Northwest Alaska they moved seasonally between the unforested interior and northern coasts, hunting caribou, catching fish on rivers and lakes, and apparently harvesting seals and other marine fauna around the Chukchi Sea and Norton Sound, though definitive evidence is sparse (Tremayne and Rasic, 2016). A series of poorly understood archaeological components follow ASTt in Northwest Alaska (and Chukotka) from around 3200 to 2200 yr. These include an apparently intensive maritime occupation known only from a few houses at Cape Krusenstern (Darwent and Darwent, 2016) and at the Un’en’en site on Chukotka (Gusev, 2022), somewhat better represented sites of the Choris phase, introducing Siberian pottery traditions, and at least in the one site with faunal preservation, relying on caribou and small seals in roughly equal measure, along with very small percentages of other fauna, including beluga whale (Darwent and Darwent, 2016). The Norton tradition may or may not derived directly from Choris, but it has a more substantial occupation, especially around the shores of the Bering Sea, where large, sedentary villages were supported by a combination of maritime hunting and net-based fishing along the lower courses of regional rivers (Shaw, 1998).

From St. Lawrence Island and the Bering Strait, north to the Chukchi Sea and Arctic coasts, the interval between 2000 and 700 yr was incredibly dynamic with the emergence of populous, competing culture groups living in sometimes massive villages in prominent locations to take advantage of predictable polynyas (ice leads) and the resulting availability of ice seals, walrus herds, beluga pods, and the migrations of bowhead and gray whales (Mason, 1998, 2009, 2016). With ongoing influences across the Bering Strait, these polities gave rise to the classic Thule culture that subsequently spread across the Canadian Arctic to Greenland and pushed south along the shores of Bering Strait to the Alaska Peninsula in the 13^th^ century CE. Thule expansion is thought to have had greater or lesser influence on the otherwise autochthonous traditions of the late Holocene Eastern Aleutians and Kodiak Archipelago (Mason and Friesen, 2017). In as yet poorly understood ways, the Inupiaq and Yupik cultures developed into what they are today through some complex relationship to these archaeological histories.

#### Northeast Pacific (Alaska) TFD Paleodemography.

In contrast to the Hokkaido/Kuril comparisons, macro-regional synchrony is a surprisingly dominant feature of the Alaska series through much of the past 5000 years as illustrated in Figure 4c. Correlated trends in TFD growth (matched with neutral trends in a minority of cases) is observed from 4500 to 4000 yr, 3000 to 2700 yr (in the north) and 2800 to 1900 yr (in the south), and 900 to 600 yr (throughout). Correlated declines are observed in three of the northern series between 4000 and 3500 yr (continuing until 3000 yr in the Bering Inland (#3) when the Arctic Coast (#1), Brooks Arctic Inland (#2) and Bering Coast (#4) had already bottomed out (#1–4). The Kodiak trend declines almost in tandem with these northern fluxes, but starts and ends somewhat later, from 3500 to 2600 yr (#8). A second interval of correlated declines (encompassing both trends that exceed the 95% confidence interval and others that do not) starts at about 1350 yr and lasts until about 900 yr. Almost all of the series share dramatic decline after 600 yr, most starting around 450 yr and continuing to 0 yr (i.e., 1950 CE). Some of this final decline, as on Hokkaido, may be attributed to the tendency for archaeologists not to radiocarbon date colonial-era archaeological sites. Nevertheless, the trend starts several hundred years too early for ‘historic’ sampling bias to account sufficiently for the overall pattern. Also notably, there are very few intervals in the last 5000 years during which the Alaska series are in direct opposition, and then only in non-adjacent regions.

As with the Hokkaido and Kuril patterns, the trends observed in Alaska can be attributed in many cases to known archaeological developments, such as the emergence of the Arctic Small Tools tradition between 4500 and 3500 yr (Tremayne and Brown, 2017) and the expansion of Norton around northern and western Alaska and the florescence of the Late Kachemak tradition in the Gulf of Alaska and Kodiak, both starting around 2500 yr and both accompanied by technological intensification in fishing, expansion of semi-sedentary villages, and the emergence of new kinds of social and political patterns (Fitzhugh, 2003; Harritt, 2010; Steffian et al. 2016). Rapid growth between 900 and 600 yr corresponds to the radiation of Thule people from the Bering Strait region south to the Alaska Peninsula or farther. Interestingly, and except for Kodiak, this last interval of major growth is effectively synchronous at the scale of resolution seen in radiocarbon TFS, meaning that it is unlikely to be driven exclusively by Thule migration, unless that was accomplished more rapidly than the century resolution of the TFDs. That is, population was growing in *all* of the Alaska coastal and near-coastal regions early in the second millennium CE, minimally implying that food was plentiful enough to support the growth.

As noted above when discussing the Hokkaido Central Interior (#13), the Alaska Forested Interior (#5) stands out from the other Alaska time series for its uniquely muted variability, with few significant excursions of growth or decline. This region covers the boreal zone around the Yukon and Kuskokwin Rivers, stretching from the southern Brooks Range foothills to the Alaska Range and across it to the Cook Inlet drainage. The bulk of data for this region comes from the Tanana River corridor east of Fairbanks. The only notable feature in this series is the very gradual decline of TFD mass over the last 2000 years, interrupted only briefly about 700 yr when all of the other Alaska series were also growing (dotted red arrow on Fig. 4c).

In southern Alaska (Gulf of Alaska, Kodiak, Aleutians), semi-sedentary winter settlements linked to smaller seasonal fishing camps have been the primary form of settlement organization for the last 5000 years. In Northern Alaska, maritime adaptations were only just forming 5000 yr, and semi-sedentary winter settlements become a feature of the archaeological records only in the last 2500–3000 years. Some of the increase in TFD representation identified as “Norton” on Figure 4c may therefore derive from increased visibility of coastal villages. On the other hand, the increase in seasonal camps, if recorded, should balance out the change. The adoption of seasonal surplus production in times of abundance would have facilitated and to some extent necessitated increased sedentism and population growth, making sense of the positive trends in the TFDs for this time period.

The other major innovation was the development of large marine mammal (whale and walrus) hunting in Northwest Alaska, possibly as early as 3200 yr, but with intensity in the last 2000 years (Old Bering Sea/Okvik, Ipiutak and subsequent cultures). Some of the increase in the Arctic Coast series (#1) may derive from the in-migration of NE Asians to St. Lawrence Island and Northwest Alaska. The Thule expansion out of Northwest Alaska did not significantly dent the presumed founding population in that region. The Thule expansion, or its influence on other populations, is seen as a new spike in populations after 1000 yr in many of the Alaskan TFDs. While these spikes can be attributed in some cases to the migration of a population from one region to another, that population was supported by the local ecosystems where they ended up, in turn implying an improved capacity to feed a growing population. Future analyses of TFD measures for eastern Siberia may reveal if the trends in Alaska are compensated by a related decline of people in Chukotka or further south or west.

#### Visual Evaluation of the Hypotheses.

Support for the hypotheses outlined near the top of this article is decidedly uneven in the visual analyses, with the Alaska macro-region presenting a more coherent and correlated set of trends than those from the Northwest Pacific macro-region. As predicted by Hypothesis 1, adjacent regions are more often positively associated than non-adjacent region, especially in Alaska. The fluctuations in the Alaska series are not only observed beyond the original three data sets (Fitzhugh et al. 2020), but also show synchrony back to almost 5000 yr in both positive and negative growth trends. This is broadly true across the Alaskan series *except* for the Forested Interior (#5), which appears insensitive to, and for the most part buffered from, the variability witnessed in coastal and near coastal (“inland”) regions. If populations in the near-coastal series (#2 and #3) were significantly dependent on anadromous fish tied to the marine ecosystem, then it is reasonable to conclude that the similarities between coastal and those inland series are consistent with the marine-focused LIONPac model.

The Northwest Pacific series are distinctly different from the Alaskan set, which is also predicted by the LIONPac model. Even so, the trends in many adjacent regions around Hokkaido and into the Kurils often lag. This is consistent with archaeological descriptions of Hokkaido culture history and settlement change from the early Jomon to Ainu intervals (e.g., Abe et al. 2016). In some intervals, adjacent regions show opposing trends and at other times the regions just seem to have unrelated patterns. The northern Hokkaido, eastern Hokkaido and Kuril time series do exhibit a pattern of growth and decline between 1700 and 750 yr, consistent with the Okhotsk expansion, but also inversely correlated with the Alaska trends, again as expected from LIONPac.

Except the Oshima Peninsula, all Hokkaido regions (plus the Kurils), have synchronized proxy population loss during the few centuries following 750 yr (black dashed line in Figure 4b). This was a time when most Alaskan populations were growing vigorously. Notably, however, Japanese mercantile interest in and contact with Hokkaido’s Ainu populations increased significantly at about the same time. It seems plausible that the synchronous decline in Ainu populations relates in part to this greater contact, and the associated introduction of diseases and exploitative treatment that came with it (see Hudson, 1999; Walker, 2001). The pan-Alaskan decline after 500 yr may also have been triggered by the introduction of devastating epidemic contagions, though the impacts show up at least a century before direct contact. That would imply that the diseases spread through Indigenous networks either across North America from Colonial contacts in the east or from Northeast Asia, across the Bering Strait. Recent reanalysis of the history of epidemic transmission from European hosts to and through Native American communities suggests that such devastating mortality typically occurred well after direct contact and the resulting disruption of Native food security and health that increased vulnerability to pathogens (Liebmann et al., 2016; Feinman and Drake, 2021).

These observations confirm the existence of macro-regional coherence between proxy population trends, especially over the last two millennia. These correspondences could be explained by a LIONPac ecological dynamic or some other spatially correlated process. Colonial impacts could account for the synchronous declining trend ca. 800 yr across the Hokkaido and the Kurils data sets, while colonial encounters were too late to explain the largely synchronous collapse across Alaska starting ca. 500 yr. Colonialism also cannot explain the synchronous population *growth* in Alaska at several earlier intervals, nor can it explain the earlier coordinated declines back to 5000 yr.

Along with any colonial impacts, taphonomic and sampling factors also probably play a role in the declining TFD trend seen across Hokkaido and Alaska data sets in the last few hundred years. The Ainu period on Hokkaido is characterized by the abandonment of semisubterranean dwellings and the adoption of above ground, wood-framed houses that leave less of a durable archaeological trace. As a result, archaeologists in the past tended to discover and excavate Ainu burials more than house or midden contexts (a practice now discouraged in law and practice), and dating was more often achieved through the cross-dating of known aged artifacts and historical documents such as iron pans (Koshida, 2004), harpoon heads (Utagawa, 1989), coins, ad brass pipes (Koizumi, 1987). The recent end of the Alaska sequences (Fig. 4c) should share some of the sampling biases noted for Ainu-aged dates. With colonial settlement occurring so close to radiocarbon “modern,” there is little incentive to carbon-date post-contact archaeological assemblages, and archaeologists there also rely on known aged artifacts and documents for dating. This bias could, in principle, be rectified by including historic and *terminus post quem* dates (e.g., the manufacture dates of trade goods and, eventually, commercially available materials) into TFDs. Doing so would require additional conceptual and methodological development, not to mention the labor involved, but theoretically there is no reason that time stamps (dates) generated from different kinds of materials and processes could not be merged in TFD analyses. Despite the likely under-representation of dates from the last 200 years in the Alaska time series, we see less of a reason to expect sampling bias in the synchronized collapse of TFDs that started before 400 yr.

Finally, we have been struck by the uniquely unimodal distribution of dates in the Oshima TFD (#14). While the overall shape is consistent with other evidence from the region, there may be some bias against Final Jomon and Epi-Jomon dates. According to the database of archaeological sites provided by Education Board of Hokkaido (https://www2.wagmap.jp/hokkai_bunka/portal) at the time of this writing (Winter 2022), 298 Oshima sites are attributed to the Final Jomon and 226 to the Epi-Jomon, while 527 and 558 sites have been confirmed in the Middle and Late Jomon, respectively. When accounting for differences in the duration of these cultural periods, these number predict 12%, 20% and finally 39% reductions between each successive phase from Middle Jomon to Late Jomon, Final Jomon and Epi-Jomon, respectively—a cumulative 57% drop from Middle Jomon to Epi-Jomon. The TFD in #14 shows an even more extreme cumulative decline, over 90% between the peak at 5000 yr (Middle Jomon) to the end of the slide around 3000 yr (Final Jomon). There could be various reasons for the discrepancy, including the possibility that the site count is less representative of demography than the TFD or vice versa. Arguably, the pottery-based chronology used to date the settlement patterns is less precise than the radiocarbon TFDs.

We cannot point to any other systematic sampling bias in these data sets, other than the sample-size problem already noted for the North/West Hokkaido coastal series (#11) and the possibility that variable sample sizes in other series could more or less subtly change the structure of TFD distributions. The degree to which we are able to interpret the TFDs over the last 5000 years with reference to existing archaeological understandings reinforces the conclusion that we have robust patterns overall, if not in all details. While gratifying, that impression does not automatically authorize the assumption that these TFDs necessarily represent *population* fluctuations as opposed to other interesting cultural dynamics. For example, we have noted archaeological changes in settlement organizations that could affect the frequency of dated components irrespective of demographic variability.

To a degree, visual comparison of time series can address the first question asked at the start of this article: we see considerable pan-regional coherence in population proxies, especially in the Alaskan data. Nevertheless, visual comparison is insufficient to address our hypotheses or assess the viability of the LIONPac model, given the need to think in at least three dimensions at once when trying to identify meaningful relationships between multiple TFD series, as well as the difficulty of maintaining objectivity in interpreting complex patterns. We therefore need a more quantitative means for comparing trend correlations across space and through time. To do this, we propose a new method to track changes in spatial autocorrelation of the set of 14 TFD series through time.

## TIME-ITERATIVE MORAN INDEX (TIMI): A NEW APPROACH TO TIME SERIES COMPARISON

Autocorrelation estimates the strength of non-random relationships in some variables between spatial or temporal units of analysis (regions or subsets of time series) within larger data sets (larger geographical spaces or longer time series). Measures of *spatial* autocorrelation, such as the Moran’s Index, are often used in geographic analyses to specify the degree to which different regions (e.g., land parcels, counties, countries) are significantly more alike (positive autocorrelation) or less alike (negative autocorrelation) than expected for randomly selected pairs of regions within the study universe (Anselin, 1988; Getis, 2007, 2008). *Temporal* autocorrelation analyses (used in time-series analysis), by contrast, seeks to identify the degree to which a value at any point in a time series is influenced by the value of the variable in past times over some lag interval (i.e., a measure of “memory” in the time series). Spatial autocorrelation is *static* and is not by itself designed to address temporal trends, although spatial autocorrelations of different time periods are sometimes compared (e.g., Han et al., 2020). Temporal autocorrelation, while incorporating temporality, does not address spatial correlations between multiple series or measure variability through time. *Periodicity* in time series can be interrogated using Fourier and similar analyses on individual series (Bloomfield, 2004) but does not directly compare the chronology of any patterning between series. The challenge we face in evaluating the question of interrelated behavior in regional TFDs is to find a way to test for correlation in trends between series based on one or another shared trait *as they change through time*.

Finding no ready-made solution to this analytical task (but see Gao et al., 2019; Han et al., 2020 for related efforts), we introduce here the ***Time Iterative Moran Index***, TIMI for short. TIMI monitors spatial autocorrelation in trends over successive time intervals through the history of time series. TIMI plots provide the means to examine *histories of autocorrelation* between time series with selected attributes. Details of the assumptions and analytical procedures are provided in the Supplementary Material. TIMI is built on the calculation of Global Moran I statistics (Moran, 1950) from comparison of directional changes in pairwise TFD values. We transform the TFD series into mean adjusted growth rates (MAGR or $\bar{r}$) at increments of 5-year intervals, grouped into moving 200-year windows to match the smoothed data.

### TIMI Method

For each time step, the TIMI procedure calculates the Moran I value (Anselin 1995) for each pairwise comparison of regional TFDs, where certain filtering criteria are applied based on the hypothesis under examination (in our case: adjacency, ecological similarity, or macro-regional unity overall and for coastal data). We compute running series of global Moran I values to evaluate each of the hypothesis. This is done by filtering pairwise relationships by means of a “1” (“include”) when the relationship is one of membership in the hypothesized in-group (adjacent/sharing a border; sharing a coastal or interior ecotype; sharing a macro-region/Alaska vs. Hokkaido+Kurils) and “0” (“exclude”) when not. For example, in testing for adjacency, we set TIMI to run paired correlation analyses only on those regions that share a border (coded as “1”) relative to the average correlation between all regions. The TIMI analyses allow us to evaluate changes in the autocorrelative relationships between different time series as they change through time. This makes it possible to evaluate the initial research questions/hypotheses temporally, to reveal previously unconsidered patterning in the data, and to further address questions about sample sufficiency and the evolution of human-environmental dynamics across the North Pacific.

**Figure 5.**
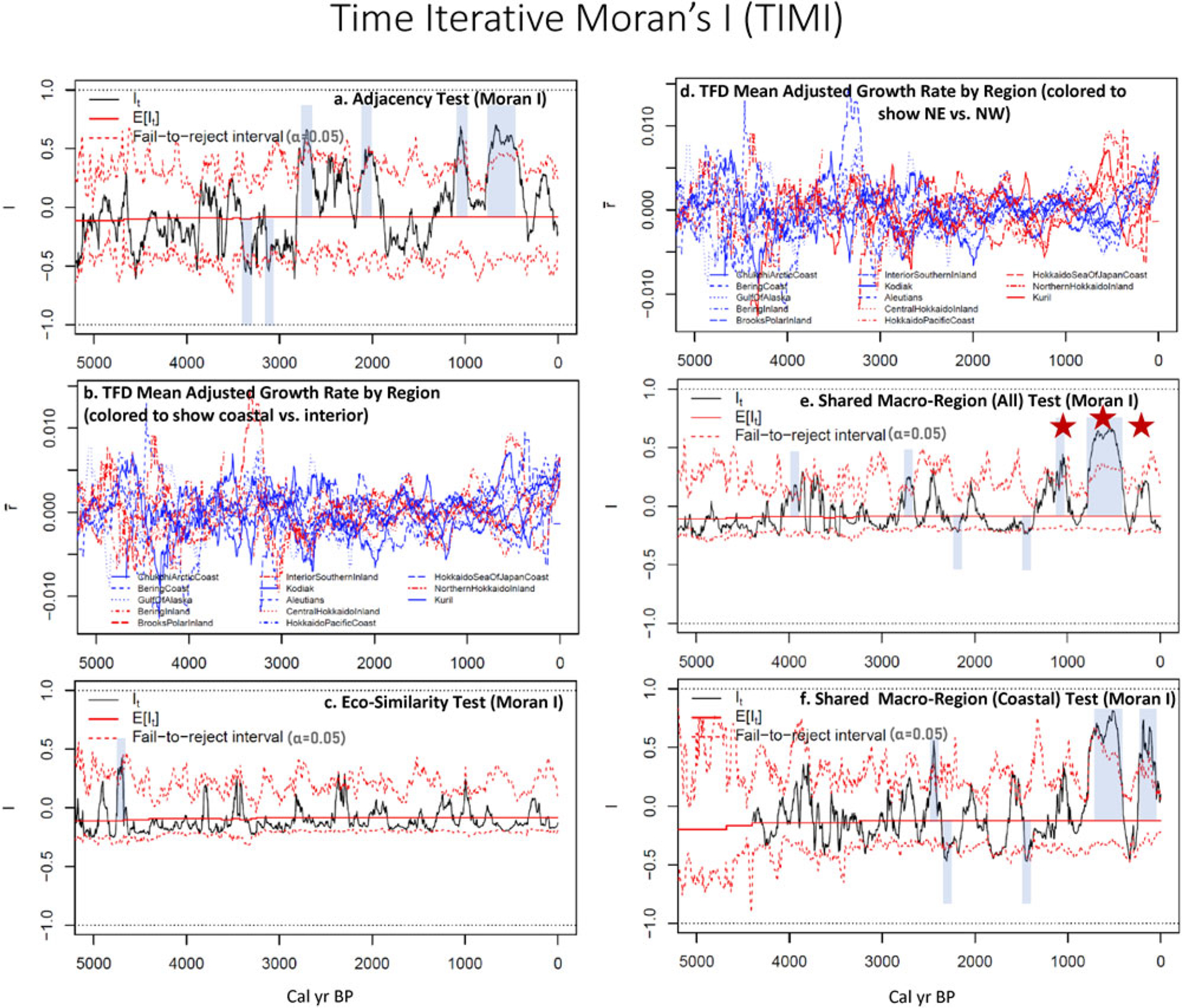
(a, c, e, f) Time Iterative Moran Index (TIMI) plots of global Moran I statistic (*I*) calculated on pairwise MAGR datasets to measure degrees of spatial autocorrelation as they change through time relative to hypothesized relationships. (b and d) Trends in Mean Adjusted Growth Rate $\left(\bar{r}\right)$ plotted for each regional time series and color coded (in graph b) for coast (blue) vs. interior (red) and (in graph d) for macro-regional association (blue for Alaska; red for Hokkaido + Kurils). (a) Moran’s I adjacency analysis (“Do adjacent regions tend to be more similar than non-adjacent regions?” Conventional spatial autocorrelation); (c) general eco-region analyses (“Are coastal vs. Interior data sets more autocorrelated within their sets than between them?”); (e) Shared macro-region analysis for all ecoregions, with stars denoting peak positive autocorrelations in the last 1100 years (“Do regions within Alaska and regions within Hokkaido autocorrelated with other regions in the same macro-region?”); (f) Shared macro-region analysis for coastal and near-coastal data sets (“Do TFD series for coastal and near coastal regions within the same macro-region autocorrelated relative to other regions and all regions in the other microregion?”). Gray overlays on panels a, c, e and f denote Moran I values that exceed the formal 95% “fail to reject” threshold (α=0.05) in a positive or negative direction. See text and Supplement for discussion of the TIMI approach to spatial autocorrelation of time series and the calculations applied.

Running Moran I plots are calculated on the mean adjusted growth rates (MAGR or $\bar{r}$), which is a transformation of the TFD plots reflecting the changing *slopes* of the TFDs in Fig. 4 (the first derivative), not the ‘raw’ TFD values themselves. The Moran I statistic estimates, for each time step, the degree to which pairs of MAGR plots are doing the same thing (increasing, decreasing, staying constant) averaged across the within-group set (i.e., the set coded for either adjacency, identical ecosystem class, or membership in the same macro-region) relative to averaged pairwise comparisons of all regions. On the TIMI plots presented in Figure 5 (a, c, e, f), any index value above the mean expected value (E[l_t_], solid red lines) indicates a greater than average positive autocorrelation in favor of the hypothesis under evaluation. Confidence intervals (*CI*s) of 95% (α=0.05) are calculated based on repeated permutations of the MAGR values randomly reassigned to dummy series (Supplement). With the confidence interval set at 95% and a relatively small number of regions included in the analyses, our TIMI results rarely exceed the “fail-to-reject” threshold. Any random error noise in the underlying TFDs would also tend to dampen TIMI values below the confidence interval levels, making it especially hard to achieve significant measures of autocorrelation. Given the typically smaller sample sizes earlier in each time series, we might expect less significant autocorrelations earlier in time. If so, then autocorrelation closer to the present may be an indication that the hypothesis is correct. Acknowledging those issues, we interpret the results with attention to both significance and the intervals spent substantially above or below the mean expected value (E[l_t_]).

Figure 5b and 5d show the mean adjusted growth rates $\left(\bar{r}\right)$ for each time series. The trends are noisy, which is expected based on our analyses of the stacked TFD series in Figure 4. Figure 5b is color-coded to identify shared eco-regions (Coastal zones = blue; Interior regions = red). Figure 5d shows the same plots with each trend color-coded to indicate Alaska (blue) and Hokkaido + Kurils (red). These MAGR plots are included to allow visual inspection of the data underlying the TIMI analyses.

Next, we discuss these results in the context of the original hypotheses.

### TIMI Results

Figure 5a, ***Hypothesis 1: Adjacency*.** *Irrespective of any larger scale oscillations, populations living in adjacent (contiguous) regions should have more correlated population trends than populations living far apart*.

Our application of the TIMI calculation for this hypothesis outputs above-expected values when growth rate trends in adjacent regions correlate more than with non-adjacent regions (hypothesis confirmation). As a result, a positive TIMI tendency reveals a trend in support of the first corollary (C1) of Hypothesis 1, that of shared growth or decline due to net fertility relative to mortality and/or in-migration relative to out-migration from non-adjacent regions. Neutral or negative pulling values would indicate more uncorrelated or inversely correlated trends in neighboring series, including net population movement from one adjacent region to another (C2).

In Figure 5a, the Moran I statistics cross the confidence threshold in the positive direction (indicating similar trends) four times, briefly around 2700 yr, 2000 yr, 1100 to 1000 yr, and again for a longer interval from ca. 800 to 450 yr. Given the sampling issues already discussed and the likelihood that the underlying TFD patterns are formed by multiple causes operating at different spatial scales, it is not surprising that the TIMI analyses rarely reach the 95% significance threshold. As a result, we are comfortable attending to persistent positive and negative excursions from the expected value (E[l_t_]). Positive TIMI autocorrelations of adjacent regions are notable for most of the last 3000 years. Earlier patterns are variable or even predominantly negatively autocorrelated. In marked contrast to intervals before and after, the TIMI plot trends negative from 1900 to 1400 yr. This was an interval of considerable population flux from one region to another in Hokkaido. It was also a time of relative constancy but low level and uncorrelated flux in the Alaskan series (Fig. 4).

In broad terms, we interpret the results of the adjacency analysis to support the hypothesis that neighboring regions are more likely than not to share similar population trends, particularly in the most recent millennia, when data sets are most robust. Differences in regional scales between Alaska and Hokkaido likely complicate this analysis, something we return to in the Discussion.

***Hypothesis 2: Eco-Region Autocorrelation*** *Regardless of adjacency, if populations are density-dependent or otherwise tuned to the unique ecological conditions in which they live, then population trends should trend together in regions sharing similar ecological/environmental characteristics and histories more than across ecologically differentiated regions*.

This hypothesis gets at the idea that hunter-fisher-gatherer populations are, to a significant degree, dependent on the resources they can harvest within their regions. It assumes that those resources are more likely to respond in parallel to similar environmental changes, including changes that alter the net availability of food (holding harvesting methods constant), and that the productivity of food harvests is driven by bottom-up ecological factors. These assumptions underly both Hypotheses 2 and 3, but we approach the issue first without regard to differentiation in how our ecozones in different locations might experience environmental drivers differently. If human populations associated with North Pacific Rim ecosystems followed the same trends from Hokkaido to Alaska members in pan-subarctic/Arctic ecosystem, this hypothesis should be supported. Accordingly, our Eco-Similarity analysis lumps all coastal and near-coastal regions together and all Interior regions together and computes the TIMI autocorrelation within each group compared to the total set of regions. Figure 5c shows the results of the Eco-Similarity test. In this case, there is little if any autocorrelation, or even patterning, in the data—a characteristic seen also in the color-coded MAGR plot in Figure 5b. We can safely reject this hypothesis.

***Hypothesis 3*.** *If spatially structured oscillations (ecologically driven or otherwise) distinguish the NE Pacific (Alaska) and NW Pacific (Hokkaido and Kurils) population growth dynamics, then populations living in different Alaska regions should be positively correlated with each other and negatively (inversely) correlated with Hokkaido and Kuril regions and vice versa*.

Hypothesis 3 embraces the spatially structured expectation of the LIONPac model. Figure 5d shows the same MAGR plot as in Figure 5c but colored for macro-region affiliation (blue for Alaska, red for Hokkaido). Despite the noisiness, there are clear correspondences between subsets of time series through various intervals. From the colors, it is particularly apparent that Alaskan series tend to track together predominantly from 2500 yr to the present, in distinction from the Hokkaido+Kuril series, which is both more chaotic and somewhat inverted relative to the Alaskan series over the last 800 years.

Figures 5e and 5f each evaluates a variation of Hypothesis 3. Figure 5e measures the extent of autocorrelation between regions within one or the other of Alaska and Hokkaido+Kurils macro-regions, irrespective of eco-zone (i.e., testing H3c). High positive values, such as the three dominant peaks between 1400 and 150 yr (starred), indicate positive correspondence within those macro-regions. Other intervals of high if not significant (at α=0.05) positive correlation are seen from 4000 to 3400 yr and, to a somewhat lesser extent, 2800 to 1900 yr. Because these analyses run both ways (looking for greater than average correspondence within both macro-regions), the greater discordance in the Northwest Pacific data sets are likely responsible for the low autocorrelation elsewhere in the series. It is notable that the interval from 800–450 yr is the most significant interval in all of the TIMI tests (Figs. 5a, 5e and 5f) except for Figure 5c (Eco-Similarity) and it is the most significant of any of the TIMI analyses in this test.

Figure 5f is an attempt to directly test the LIONPac model by looking at the coastal set of samples by macro-region (Hypothesis H3a). The result shows more accentuated positive *and negative* autocorrelation. While significant positive autocorrelation is indicated again for the interval of 650–450 yr in Figure 5e, it is less significant than for the total macro-region analysis (Fig. 5f). Based on these two tests, we would have to reject the narrowly defined LIONPac model. Our effort to quantify the degree of autocorrelations using TIMI forces us to conclude that the archaeological population proxies are not predominantly patterned as expected if only populations in coastal regions were affected by an east-west oscillating (marine) ecosystem dynamic. This does not mean that the LIONPac model is necessarily wrong, or that there are not some other forces driving regional population variability. It is highly likely that the rejection of the marine specified hypothesis (H3a) is in part a function of the greater heterogeneity of the Hokkaido TFDs compared to those of Alaska. We explore the factors that could be behind this below and in the Discussion.

***Hypothesis 4*.** *If regional populations followed unique and idiosyncratic histories prior to the influence of commodities market systems and before exposure to epidemic diseases like smallpox linked these regions together in economic and demographic systems, then Hokkaido and Kuril population trends should become correlated around the 7th-9th century CE, while the Alaska series should remain uncorrelated until after the 18th century*.

This hypothesis formalizes the proposition that the inverse trends initially identified between southern Alaska and the Kuril Islands (Fig. 3d; Fitzhugh et al. 2020) are, in fact, artifacts of the expansion of commodities trade networks, epidemic diseases, and ultimately colonial incursions, first in maritime Northeast Asia and later in Alaska. The increase in autocorrelation seen in Figures 5e and 5f after 1400 yr, but especially after 800 yr, and the more strongly aligned positive growth rates seen in the Hokkaido plots (red in Fig. 5d) from 900–500 yr might be taken as evidence for population growth connected to beneficial consequences of access to trade with Japan, Sakhalin, and the mainland by Satsumon, Okhotsk, and ultimately Ainu communities. The expansion the Okhotsk may also have been driven by the development of more productive sea mammal hunting techniques, potentially opening a new niche for Okhotsk populations to thrive and grow.

This explanation does less to help us make sense of the auto-correlated Alaskan series through time. Colonial impacts arrived in Alaska between 200 and 100 yr (1750–1850 CE), at the very tail end of radiocarbon discrimination. It is well-documented that Alaska Native populations were heavily impacted by introduced diseases and associated famines in waves during the 19^th^ and early 20^th^ centuries (Fortuine, 1989), while Ainu communities were ravaged by conquest, resettlement and coerced labor starting in the 17^th^ century, but especially in the late 19^th^ and early 20^th^ centuries (Walker, 2001). These colonial impacts doubtless contribute to the declining population trends on both sides of the North Pacific in the last couple of centuries, but they do not explain the earlier onset of these trends, especially in Alaska starting ca. 400 years ago.

One limitation in evaluating the colonial impacts hypothesis (H4) from the macroregional ecology hypotheses (H3) is the possibility that the TIMI analyses are sensitive to decreasing sample robustness and consequently more noise in TFD signals as we move back in time. While further investigation of the TIMI method will be needed to clarify how sample robustness relates to TIMI outputs, for now we recognize that the presence of strong autocorrelation in the macroregional data sets primarily in the last 1100 years (Figs. 5e and 5f), could support both Hypotheses 3 and 4, and both could be in operation together. For the time being, we can only cleanly reject Hypothesis 2. There is no support for the idea that coastal populations around the North Pacific Rim were locked into similar trends of growth and decline in distinction to terrestrial populations on both sides of the Pacific/Bering Sea.

## DISCUSSION

### Findings

The analyses presented here illustrate the importance of using large comparative data sets when attempting to interpret the paleodemographic significance of archaeological proxy data. Expanding the number of TFD curves beyond those that initially triggered our curiosity reinforces our confidence in some of the patterns that inspired the LIONPac model, particularly for Alaska. By investigating the relationships between adjacent regions, we have found most trends mutually interpretable and consistent with known archaeological histories of both the Northeast and Northwest Pacific. Framing our investigation around hypotheses of adjacency, ecological zonation and macroregional characteristics helped to formalize our expectations of inter-regional population patterning and human-environmental interactions.

Including larger numbers of regional data sets in paleodemographic analyses, as we have done in this paper, yields both benefits and difficulties. The ability to track the behavior of TFDs from neighboring regions (both adjacent and non-adjacent) provides greater perspective for the interpretation of individual series. In tandem with other archaeological evidence, these comparisons allow us to distinguish between inter-regional mobility, large-scale migration, and internal population growth and decline. As emphasized early on, TFD trends can arise from several different data-generating processes, often simultaneously. As a result, they are inherently noisy and should not be interpreted directly as population change without considering other possibilities. The ability to compare adjacent and near-adjacent TFD time series is one way to identify meaningful and interpretable features, particularly where the series show systematic correspondence, such as the correlated peaks and declines in the Alaska TFD stack (Figure 4c) and when compared with other archaeological information.

The TIMI autocorrelation metric in principle promises to help simplify comparisons between time series. Our application of the new technique here has shown utility, especially when evaluating between different TIMI plots created under alternative assumptions (e.g., adjacency vs. eco-similarity). Additional development of the technique and the way it calculates confidence intervals can be expected to provide more definitive applications. However, it is also quite likely that the results accurately reveal deficiencies in the expectation of simultaneous (if inverse) correlations in population trends on either side of the North Pacific.

The source of the failure is found to be in the Hokkaido data (as opposed to the Alaska data, which meet the expectation of correlated trends within the macro-region to an unexpected degree). The greater asynchronicity and heterogeneity between Hokkaido data sets means that autocorrelation is rarely identified. The TIMI metric, as we used it to determine autocorrelations within macro-regions (Hypothesis 3), did not distinguish the output from one macro region vs. the other. Doing so would allow us to evaluate the extent to which Alaska and Hokkaido may have different historical trajectories of autocorrelation. Visual analysis makes it likely that the Alaska set was often more correlated, which may ultimately be linked to environmental variability.

That heterogeneity in the Hokkaido series does not necessarily undermine the LIONPac model, but it does suggest that there was a lot of gradual shifting of populations from one part of Hokkaido to another, potentially driven by economic, ecological, epidemic, social, and/or political factors discriminating population dynamics at rather local levels. The Alaskan population changes were often faster and more synchronized. Some of these were driven by migration, others by technological innovations and perhaps environmental changes. We propose that one reason for the differences in these two regions may come down to differences in spatial scales. Hokkaido measures just over 84,000 km^2^, and we divided it into 6 regions (avg. ca. 14,000 km^2^ per region). The Kuril Archipelago spans a longer linear distance but is only 10,500 km^2^ in area. Alaska, not including the southeast “Panhandle” (which was excluded from this analysis) spans just over 1.66 million km^2^. We divided Alaska into 8 areas (ca. 208,000 km^2^ each). In other words, the average Alaska region is almost twice the size of Hokkaido and about 15 times the size of the average Hokkaido region used in this analysis. Why might this matter?

Although we created our units based on the needs to maintain appropriate sample sizes, the size of our units of analysis can also be expected to capture different scales of human behavior. Hunter-fisher-gatherers move more or less often and do so to differing degrees depending on their lifestyles and the reasons for moving. While there can be many reasons for moving, in general, larger and longer migrations are less common because there are distinct advantages to making a living in a place one knows well and where one has strong social networks of support. Hokkaido’s heterogeneous TFDs likely capture something of the dynamic mobility of small, largely autonomous, semi-sedentary communities over time. If we were able to break any of the Alaska regions into smaller subregions, we might expect to discover similar kinds of heterogeneity between them.

**Figure 6.**
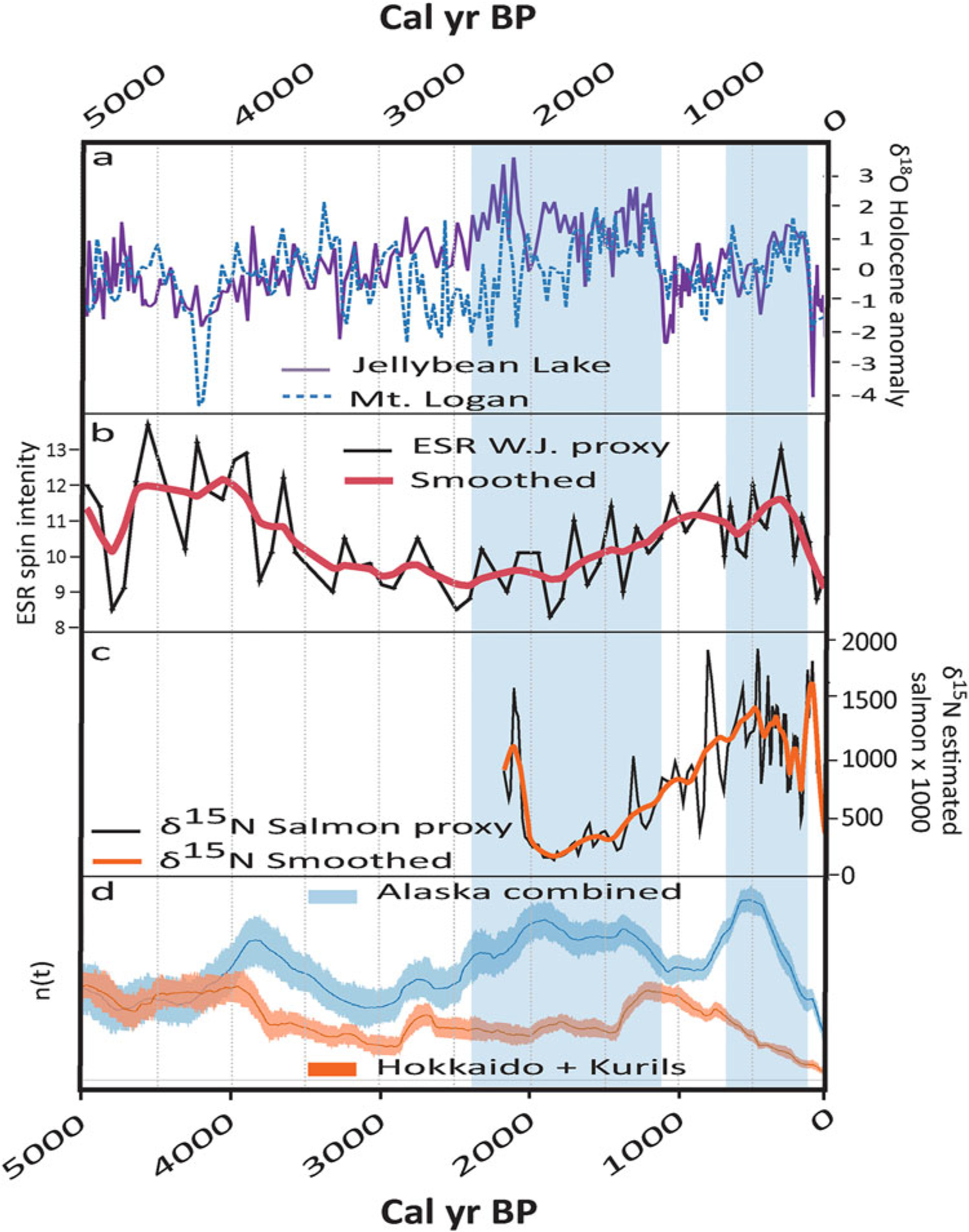
North Pacific climate proxies compared to composite Northeast vs. Northwest Pacific paleodemography proxies (TFDs). Paleoclimate/paleoecology proxies (6a-6c) as described in Fig. 3a, 3b, and 3c. (3a: Anderson et al. 2005 and Fisher et al. 2008; 3b: Nagashima et al., 2013; 3c: Finney et al., 2002). d. North Pacific archaeological population proxies (CRFPAB, taphonomically corrected) combining all Hokkaido+Kurils (orange) and all Alaskan (blue) series and showing 95% confidence envelopes based on the CRFPAB procedure (Supplemental Material). While series drawn from smaller regions reveal sometimes complex and contradictory structures through time, when aggregated, the last 2500 years can be seen to have had largely anti-phase population trends between the eastern and western North Pacific. Transparent blue overlays represent intervals of strong Aleutian Low pressure after Nagashima et al., 2021.

But just as dividing spatial units (where possible, given available sample sizes) might yield more inter-regional heterogeneity, combining them enables us to factor out the relatively local heterogeneity. We take this approach in Figure 6 (d), *combining* the TFD data sets into just two composite TFDs series, one each for Alaska and Hokkaido+Kurils. The result reveals the large-scale temporal fluxes characterizing both cases. The Hokkaido+Kurils series is somewhat less pronounced than the Alaska series, but both fluctuate significantly and both shows similar *and largely* inverted multi-century to millennial-scale patterning. If we are comfortable with the assumption that environmental dynamics could have net effects on populations that are only apparent when we scale back from the “regional” to the macro-regional, then these results would appear to support some version of the LIONPac model, at least so far as to justify the pursuit of robust paleoecological and zooarchaeological data that can more directly test the ecological predictions of the model themselves.

### Future of the LIONPac Model

The analyses above neither fully supports nor refutes the simple oscillation model of human population fluctuations in response to reversals of ecological productivity (or some other underlying cause), but in fact we would have been surprised if it had, for two reasons. First, LIONPac is modeled on a simplified version of 20^th^ century decadal oscillations projected back in time and assumed to scale up to multi-century to millennial versions of the decadal patterns. This was a useful strategy to form a model on which to explore spatio-temporal patterning at a grand scale, and the model may still reveal an underlying reality about North Pacific ecosystems and human demography. But if so, that ecological reality is more complex than a simple ‘seesaw’ oscillation tied exclusively to marine ecosystems. For one thing, the ecological drivers may work differently at the longer time scale. For another, humans are not fish (or lemmings) that tightly follow the variance in their food supply. When times get tough, people broaden their diets, store for later, move more often, visit their neighbors, attempt to make resources more productive through intensification and specialization, fight for access to scares resources, and under the worse cases, emigrate to distant lands. Human paleodemography, instead of measuring simple changes in fertility and mortality, tracks the population related outcomes of all these complex processes.

A second reason to complicate the LIONPac model is more specifically related to the ecological mechanisms thought to underlie it. The PDO was initially discovered because of coherent patterns observed in different parts of the eastern North Pacific basin, where currents and climate are structured by their delivery across the largely unobstructed ocean. Climate dynamics on the western side of the basin are more complex, influenced not only by ocean currents and cyclonic storm patterns but also by continental weather patterns out of Siberia and tropical East Asia. Not only this, but the western manifestation of the PDO and similar North Pacific SST anomalies are more spatially focused in the west such that the patterns of positive and negative anomalies in any given phase are close together and the direction of the anomaly could switch from anomalously warm to cold over the distance from Kamchatka to Hokkaido. If those conditions are not geographically fixed, then we would expect more variability in the ecological responses to conditions that might be more phase-consistent in the Northeast Pacific.

The degree to which spatial autocorrelation is not specifically tied to the coastal assemblages provides another opportunity to revise our thinking about the LIONPac model. We could conclude from this finding that whatever is causing the major swings in the TFDs (which we take to be human population flux) it is not ecologically relevant (or at least specific to the integrated marine ecosystem). But it is necessary to acknowledge that in both Alaska and Hokkaido, the marine ecosystems projects itself into the interior in a significant way through the action of anadromous fish, primarily the various species of Pacific salmon (*Oncorhynchus* sp.), and Arctic char (*Salvelinus* sp.) in the northernmost regions. Anadromous fish carry ‘unearned’ nutrients deep into the interior regions surrounding the North Pacific Rim and feed a terrestrial ecosystem through the consumption and decomposition of their carcasses. As a result, human communities deriving significant proportions of their subsistence economies from these fish might be said to be as dependent on marine ecosystem dynamics as coastal dwellers, and a marine ecological regime shift could control not only coastal population dynamics but also those in more terrestrial locations.

The LionPac model is founded on the inference that a set of systematic climate patterns creates long-interval regime shifts in North-Pacific fisheries ecology, which in turn affects the food security of fisheries-dependent communities. While we don’t yet have the evidence to examine the direct paleoecological link to human subsistence (which will require abundant zooarchaeological and archaeobotanical research at the intersection of the marine food web and human diet), we can look at available paleoclimate proxies and attempt to interpolate between them. The difficulty remains that the available proxies are often inconsistent, and researchers are still trying to understand how to integrate proxy series of different kinds from somewhat different locations, and with differing precision and age-model control (e.g., Kaufman et al., 2016).

In Figure 6 we replot the last 5000 years of paleoclimate/paleoecology proxy data from Figure 3. The trends in Figure 6a, 6b, and 6c are sometimes interpreted as coupled with or forced by changes in Aleutian Low dynamics. They include oxygen isotope proxies from the Jellybean Lake core (Anderson et al., 2005) and the Mt. Logan ice core (Fisher et al., 2008) in the Southwest Yukon (Fig. 5a), a Japan Sea sediment record tracking change in the seasonal dynamics of the East Asian westerly jet stream (Nagashima et al., 2013), and Bruce Finney’s δ^15^N isotope salmon proxy series from Karluk Lake on Kodiak (Finney et al., 2002). Blue shading overlaid on the Figure 6 stack are Nagashima et al.’s (2021) ‘strong western AL’ intervals (published as part of this special issue). At minimum, if there were a large-scale ecological regime shift pattern operating over multi-century to millennial scales, we expect to see correspondence between the paleoclimate signals proxy population curves. Given the greatest autocorrelation seen in macroregional comparisons, for Figure 6d we combine all the regional data sets into aggregate time series by macro-region. The result is a much stronger and mostly inversely correlated comparison of Northeast and Northwest Pacific population dynamics.

While more environmental proxies are needed, the results do show reasonable, though not perfect, correspondence between paleoclimate proxies (Fig. 6a, 6b), ecological regime shifts (Fig. 6c) and human population modes (Fig 6d). Nagashima et al. (2021) found that the Aleutian Low became a more prominent climate feature in the late Holocene North Pacific and that it varied in strength and positions at the millennial scales. The blue overlay bars on Figure 6 represent intervals for which they find multi-variate evidence for a strong westward shift of the AL storm tracks in consort with a more northerly shift of the East Asian jet stream. Based on processes operating over the past half century, a westward shift of the AL storm track should translate into reduced productivity in the Gulf of Alaska, potentially shifting the locus of ecological productivity away from southern Alaska and towards Hokkaido. Because the marine ecosystem of western and northwestern Alaska is linked to the Gulf of Alaska through nutrients advected via of the Alaska Coastal Current and the dynamics of upwelling in the eastern Aleutian passes (Hunt and Stabeno, 2005), more northern parts of Alaska, especially near the coast could be affected by the impacts of the westerly shift of the AL as well.

This interpretation is intriguing and deserves more research into both the mechanisms and the empirical evidence of past ecological changes. Even so, the correlations in Figure 6 are only partial and we do not propose such an ecological model to be more plausible than other explanations at this stage. In some cases, the human population proxy data sets need expansion, more interrogation, and more detailed consideration in the context of archaeological evidence. Here we have not been able to consider independent archaeological predictions for population growth and decline. For example, if robust TFD collapse is in fact indicative of serious population failure or out-migration, we might expect to see archaeological indicators of subsistence stress, increased mobility, or greater discontinuity in occupation histories leading up to collapse. By that token, we might expect rapid growth to correspond to evidence of increased wealth differentials and elaboration of communal ceremonialism, feasting and conspicuous consumption in times of plenty. Altogether there are many different lines of evidence to explore growing from the deceptively simple effort to make sense of aggregated TFD proxy models.

### Human Ecology, Cultural Resilience and Implications

A particularly salient line of investigation that we can only touch on here is the importance of paleodemographic proxy analysis for the interrogation of human socio-ecological resilience. A growing concern among archaeologists, historical ecologists, and community planners today ties to better understanding what communities and resource managers can do to improve the resilience of vital human-environmental relationships in times of growing uncertainty. Study of deep histories of socio-ecological interactions, focusing on the legacies of social and economic organizations and engagement with dynamic ecosystems under climate variability, promises to provide a richer field for understanding vulnerable states and how to strengthen socio-ecological resilience for the future. Indications of human population declines, especially rapid “collapses,” raise questions about causes. Why did Norton communities in the Bering coast and inland regions of Alaska decline sharply between 1400 and 900 yr (if they did, as the TFDs suggest) and did their organizational response to the challenges of the era erode their capacity for resilience, potentially making them more vulnerable to invasion (or rescue) by Thule immigrants? Why did Gulf of Alaska and Aleutian populations decline significantly at the same time, and did they respond differently, perhaps with more resilience, perhaps by expanding their social networks throughout the region, intermarrying and having larger families better able to defend against encroaching Thule groups on the Upper Alaska Peninsula (Fitzhugh, 2003; Misarti and Maschner, 2015)? Were Okhotsk communities in the Kurils isolated from their social security networks as attention in Hokkaido gradually shifted to supplying trade goods for Japanese and Manchurian trade (Fitzhugh et al., 2011)?

As we face the growing threat of accelerating climate change and its uncertain implications for future marine dynamics, we should pay more attention to variability in past resilience and applying that information to support the resilience of vulnerable communities in the present and future. Resilience theorists have shown that systems often become brittle as they are pushed to the limits of their performance (Nelson et al., 2012). Resistance to change and economic intensification to maximize yields have been shown to work in the short run but create increased vulnerability to unpredictable perturbations and greater risks of failure in the long term (Dugmore et al. 2012; Nelson et al., 2016). North Pacific archaeology, supported by proxy paleodemography and its integration with expanding paleoecological research, will help us better understand the nature of ecosystem variability beyond the scale of historical and instrumental data sets and to see how communities of the past faced, and were affected by, past changes, some of which appear to have had real implications for populations.

### Connecting People, Cultures and Ecosystems Across Oceans – Thoughts on Integrating the Non-Local and Local for the Benefit of Communities.

Importantly, the records we have been examining in this paper reflect the deep histories of Indigenous communities living today around the North Pacific Rim. Those communities established rich and robust knowledge and effective ways of interacting with the North Pacific natural world. We can assume that the population crises reflected in at least some of the proxy models examined helped shape that knowledge and the approaches that have worked to support these communities in the face of past loss and hardship. North Pacific people were not constant. They did not always live as they do today (or did upon first contact with writers). Opening a window into the dynamic past of population histories allows community members and others to appreciate the complex pasts that led to the present. Whether or not the LIONPac model describes a realistic mode of ecological periodicity in North Pacific ecological regimes, communities have been and will continue to be affected by, and to respond to, environmental, socio-economic, and political dynamics that can threaten the security of local adaptations.

## CONCLUSION AND OUTLOOK

### What does it all mean?

Quaternary paleoecologists are well aware that past environmental changes have often exceeded the bounds of recent variability, and world-class climate modelers seeking to forecast future impacts under alternative scenarios of anthropogenic inputs regularly include paleoclimate proxy data to evaluate the performance of model assumptions. Even so, it is often challenging to compile robust paleo-records to track the dynamics of past climates and environments. Given the vital importance of fisheries to contemporary fishing communities and to the global food supply, it is as important to gain better understanding of the histories of marine ecosystems as it is to reconstruct past climates (which of course are related problems). This effort is still in its early stages. Arguably, it is even more important to understand how *people* have interacted with marine ecosystems and how those systems have in turn affected human ways of life and societies. People have been living around and by means of the greater North Pacific Rim ecosystem for 5000 to 10,000 years or more, and yet we have very limited understanding of how those communities managed their lives and economies in the face of significant climate and ecosystem shifts.

In this paper, we examined the proposition that a North Pacific Rim long-interval oscillating ecosystems dynamic has been operating at millennial scales through the Holocene. Lacking the data to consider the proposition directly, we considered it through the lens of archaeological paleodemography proxies. Preliminary analysis (Fitzhugh et al., 2020) had shown that a selection of four proxy population reconstructions from southern Alaska and the insular Northwest Pacific (Kuril Islands) fluctuated as if facing century to millennial scale oscillating cycles of food availability and insecurity. We call the hypothetical socioecological model the *long-interval oscillation in the North Pacific* or LIONPAC model. The model envisions neighboring populations experiencing synchronous shortages in some intervals and surpluses in others, *in opposite phase* on either side of the North Pacific. One plausible mechanism to explain these patterns is the presence of a multi-century to millennial scale ecological regime dynamic driven by persistent intervals of strong and low Aleutian Low dynamics.

Building on the original analysis, here we have brought together fourteen archaeological demographic proxy models from across Alaska, Hokkaido, and the Kuril Islands to test the integrity of the original interpretations and to facilitate broader analyses. To our knowledge this represents a novel exploration of a large set of archaeological population proxies, taking advantage of the greater interpretability of neighboring distributions with partially shared archaeological, cultural, demographic, and environmental histories. We conducted qualitative visual comparisons and introduced the Time Iterative Moran I (TIMI) spatial autocorrelation method to compare archaeological temporal frequency distribution (TFD) trends quantitatively. Our population models are all built from cleaned archaeological radiocarbon data sets assembled into occupation-index-based temporal frequency distributions or TFDs.

Results indicate considerable population dynamism around the North Pacific. Adjacent and neighboring trends show strong associations in time and trend that reinforce the assumption that (for the most part) these proxy models represent meaningful variation in the intensity of archaeological deposition, usually as a reflection of changing population densities, reflecting net fertility, mortality, in-migration, and out-migration. The combined analyses culminated in the test of several hypotheses constructed around expectations for demographic relationships between neighboring regions (adjacency), coastal vs. interior locations (as a proxy for targeted ecological adaptation and susceptibility to ecologically differentiated fluctuations in resource availability), and membership in one or another microregion (Northwest or Northeast Pacific). Evaluating the predictions of these hypotheses, we show that adjacent regions were most likely to share demographic patterns and that membership in one or the other macro-regions contributed strongly to the likelihood of shared demographic trends, especially for Alaska. Interestingly but not surprising in hindsight, the ecosystem similarity test failed (Fig. 5b,5c), revealing that ecosystem dynamics, and hence human food security, is not strictly determined by whether people lived on the coast or interior.

Differences in spatial autocorrelation of Alaska vs. Hokkaido proxies suggests either that Hokkaido populations were far more spatially dynamic than those of Alaska, or that the different sized spatial units characterizing these two regions, respectively, open windows into different scales of demographic behavior. As such smaller regions, such as those within Hokkaido and the Kurils, may be characterized by more inter-regional movement and population shifts; whereas behavior at these scales operates ‘below the radar’ at the larger scales reflected in the Alaskan regions, which are thereby more reflective of more macro-scale (and interestingly more correlated) trends. Even so, Alaskan archaeological history is characterized by several major migrations, and these can also be detected in the population proxy modes across the Alaska data sets.

While the LIONPac model remains hypothetical at this point, the aggregate (macro-regional) comparison (Fig. 6d) suggests that even with greater heterogeneity in details, the Northwest Pacific data (Hokkaido+Kurils) encompass significant net growth and decline in generally opposing phase to that of the Alaskan series. This comes closest to supporting the hypothesis that populations were under the influence of some coherent millennial scale dynamic. The more regularly oscillating Alaskan pattern, in particular, comes closest to supporting something like the LIONPac model. This is especially true when viewed in consort with available paleoclimate and paleoecological proxy series that carry the signal of millennial scale fluctuations in Aleutian Low strength and position (Fig. 6).

### Where do we go from here?

The paleodemographic proxy data presented in this paper contain a wealth of information that should be scrutinized, challenged, tested and interpreted by researchers interested in understanding the dynamics of past populations around the North Pacific. We should seek to fill the gap in distribution around the Chukchi and Bering Sea by building a more comprehensive set of radiocarbon data from sites around eastern Siberia and the Sea of Okhotsk beyond Japan.

The TIMI autocorrelation analysis is experimental and needs refinement to be most useful for comparison of contemporaneous trends and time series. We think the method holds potential for quantitative analysis in comparative time series analyses where visual evaluation is challenging. This could prove important in Quaternary paleoecology, paleoclimatology, archaeology or any similar field that produces and compares stacked time series to identify correlated features and infer causal relationships.

Finally, we believe that interdisciplinary perspectives such as those presented here are essential for improving our understanding of critical socio-ecological dynamics that have fallen out of knowledge but are especially critical in managing intensively exploited ecosystems under conditions of growing climate change and futures that are less predictable than the recent past. Interpolating this ancient knowledge from archaeological assemblages and environmental proxies and linking it to our best available understanding of human-environmental processes around the North Pacific is needed for anticipating potential future challenges and supporting the ongoing resilience of resident human communities and the ecosystems they depend on.

## Supplementary Material

Appendix

## Figures and Tables

**Table 1.** Archaeological radiocarbon total and effective sample sizes used to produce Figures 3(d–g), 4, and the TIMI analyses in Figure 5.

Region	Total sample (n)	Effective sample size (n_eff)
1. Arctic Coast (AK)	324	194.18 < n_eff<203.26
2. Brooks Arctic Inland (AK)	278	178.14 < n_eff < 188.04
3. Bering Inland (AK)	192	116.81 < n_eff < 125.44
4. Bering Coast (AK)	162	105.78 < n_eff < 112.66
5. Forested Inland (AK)	522	401.99 < n_eff < 417.88
6. Aleutians (AK)	350	233.46 < n_eff < 245.02
7. Gulf of Alaska (AK)	307	222.99 < n_eff < 232.07
8. Kodiak (AK)	304	200.19 < n_eff < 210.16
9. Kuril Islands	364	178.6 < n_eff < 186.09
10. E. Hokkaido	327	155.81 < n_eff < 163.41
11. N/W Hokkaido	76	33.9 < n_eff < 38.85
12. N. Interior (HK)	160	120.92 < n_eff < 128.22
13. Central Interior (HK)	1117	431.52 < n_eff < 443.21
14. Oshima: n=321	321	122.8 < n_eff < 129.35
