# Supplementary material for: Human Paleodemography and Paleoecology of the North Pacific Rim from the Mid to Late Holocene
Source: Quat Res. Author manuscript; Available in PMC 2024 Aug 14. (PMC11323057; doi:10.1017/qua.2022.35)
Supplement: Appendix [file NIHMS2011082-supplement-Appendix.pdf]

Paleodemography of the North Pacific: Supplemental material

2022-06-16

Contents

1. Overview and justification of the radiocarbon TFD analysis used in this study **2**

1.1. Paleodemographic Inference using TFDs – approaches and caveats 2

1.2. Generating and Interpreting North Pacific Archaeological TFD Curves 3

1.3. Analyzing Proxy Population Time Series 5

2. Data management **6**

2.1. Cleaning the Hokkaido data 6

2.2. Cleaning the Alaska data 12

2.3. Packaging the Hokkaido and Alaska data for TFA 18

3. Formal and computational details of the composite redundancy filtering through presence-absence buffering (CRFPAB) procedure **19**

3.1. Calibration 19

3.2. Redundancy filtering through presence-absence buffering (RFPAB) 21

3.3. Composite RFPAB (CRFPAB) 22

3.4. Taphonomic correction 23

3.5. Analysis 24

4. Formal and computational details of the time iterative Moran's I (TIMI) analysis **59**

4.1. Calculating Moran's I time series for MAGR when MAGR is unavailable for some regions at certain points in time 59

4.2. Permutation tests 61

4.3. Computation 61

4.4. Results 69

1. Overview and justification of the radiocarbon TFD analysis used in this study

1.1. Paleodemographic Inference using TFDs – approaches and caveats

Estimating human population history is both an essential element to understanding long-term human-environmental dynamics and one of the more difficult tasks in archaeology (Kelly, 2018). Throughout the second half of the 20th century, temporal frequency distributions (TFDs) built from counts of dated artifacts, burials, houses, rooms, sites and site areas within study regions have provided imprecise population estimates. Over the past two decades, the ability to aggregate large radiocarbon data sets has allowed us to improve on these methods, under certain conditions (Rick, 1987; Williams, 2012; Brown, 2015, 2017; Fitzhugh et al., 2016; Crema et al., 2016, 2017; Robinson et al., 2019; Kelly et al., 2021; Price et al., 2021; Shennan and Sear, 2021; Crema, 2022). Efforts to aggregate and archive data in digital repositories and targeted attempts to compile archaeological radiocarbon databases at up to-continental scales (e.g., Canadian Archaeological Radiocarbon Database /CARD: https://www.canadianarchaeology.ca; Kelly et al., 2021; Palmisano et al., 2022) have made it possible to compile TFDs of dates for many regions of interest. In TFD paleodemography (based on events dated by radiocarbon or other chronometric methods), accumulation rates are sought as proxies for population change. The resulting graphs are taken to reflect trends in population histories (e.g., Figs. 2d and 4 in the main text).

Even so, the shapes of TFDs are inevitably biased, not only by the interests of the researchers who selected the original samples for dating (sampling bias), but also by underlying issues of archaeological visibility and preservation (what remains to be sampled; taphonomic bias). Where research histories, visibility and preservation issues can be controlled and mitigated, TFDs track the relative accumulation rates of dated archaeological materials left by people in the past in focal sampling regions. Changes in the per capita rate of creation/deposition of dateable materials will also factor into the shape of TFDs. For example, changes in settlement organization (moving from single to multi-family dwellings), in the practices of residential mobility (from highly mobile to more sedentary or vice versa), or in approaches to ceremonial activity (e.g., in feasting practices, ceremonial burning of houses following the death of an important resident) introduce non-demographic influence on TFDs. Such cultural factors might amplify, mute or overwhelm demographic signals represented in TFD trends. In sum, TFDs can be taken as demographic proxies when sampling, preservation and cultural changes can either be ruled out or mitigated. More pragmatically, TFDs serve as hypothetical paleodemographic models, and their interpretations should include efforts to identify non-demographic influences when possible.

In many cases—especially when aggregated data come from numerous prior research projects with varying degrees of documentation—it is not possible to rule out all of the various forms of bias (especially sampling bias). In these cases, paleodemographic researchers often make the working assumption that, with large enough data sets drawn from numerous independent sources, the idiosyncrasies of the original research projects will be statistically neutralized (Rick, 1987). This assumption makes many archaeologists nervous and has led to a strong undercurrent of skepticism (Contreras and Meadows, 2014; Attenbrow and Hiscock, 2015; Hiscock and Attenbrow, 2016). One form of known systematic taphonomic bias relates to the loss of archaeological visibility/preservation reflected in the overall positive trends observed in most raw TFD curves. Surovell and colleagues (2009) have argued that the deterioration of the archaeological signal over time can be estimated (at least globally) as a function of the changing rate of preservation of volcanic tephras globally which also deteriorate over time.

While unique TFD trajectories should contain demographic information, perhaps predominantly so, they provide no inherent basis on which to evaluate their demographic relevance. In this study, our approach to the uncertainty in individual radiocarbon-based TFDs is comparative. Rather than interpreting any single TFD as a clean representation of past population trends, we assume that TFDs are noisy and the product of multiple influences. We look for structural coherence in spatio-temporal features visible in the comparison of multiple time series. The greater the coherence in data structure between series, the more likely the trends are meaningful at scales larger than individual series, and not driven by idiosyncratic research histories or local preservation factors (see Contreras and Meadows, 2014; Brown, 2015, 2017). Ruling out non-demographic

cultural influences requires consideration of the archaeological evidence associated with the TFD data sources themselves. We address cultural considerations when reviewing the regional series in the main text.

In this study, after addressing known statistical and taphonomic biases by applying the CRFPAB procedure and taphonomic correction discussed below, we conduct visual comparison and time-incremental spatial autocorrelation analyses of thirteen radiocarbon TFDs from Alaska, Hokkaido and the Kuril Islands. We aim to isolate variability that can be attributed to demographic changes and to use them to evaluate the hypotheses above. Demographic processes underlying net population change include intrinsic fertility and mortality as well as in-migration and out-migration.

Ideally, we would define localized areas for construction of TFDs and allow spatial correlations to identify the unique spatial scales of meaningful variability. Limitations in archaeological radiocarbon sample sizes force us to define spatial units larger than might be ideal, especially in Alaska. TFD analysis is inevitably affected by the spatial boundaries imposed, as noted above. Any scale of aggregation will average the signals of TFD generating processes encompassed within the aggregation. For some questions, the ideal scale may be the site, settlement or neighborhood, and for others, much larger areas may be more appropriate. While the minimal analytical unit is set by the need for sufficient sample size, there is no limit at the maximum unit size, aside from the challenges of linking aggregate trends to underlying causal processes. The logical implications of these considerations are that smaller sample areas (with fewer dates) will generate a greater abundance of idiosyncratic distributional structures (peaks and valleys) in sample distributions (i.e., sampling error), while too-large areas (with more dates) will entangle too many, more localized and independent processes that could wash out stronger, but more local signals. Where sample sizes are small across large areas, both problems are compounded and trends are least interpretable. Ultimately, spatial scale needs to be established with consideration both for sample sufficiency and the scale/s at which the processes of interest may be expected to have operated. Spatial scale mismatch is a real hazard in interpreting TFDs. This is one reason to define smaller units and look at structural patterns shared between them. Table 1 in the main text reports the numbers of dates included in each set, as well as estimates of effective sample size after penalizing redundant dates from the same locations.

1.2. Generating and Interpreting North Pacific Archaeological TFD Curves

In brief, the procedure we apply for synthesizing radiocarbon datasets into TFDs—which we have labeled 'composite redundancy filtering through presence-absence buffering' (CRFPAB)—is a three-step approach:

1. For each archaeological site, a binary presence-absence time series is derived first by placing a binary 'presence-absence buffer' around each of the site's calibrated age estimates, extending for 100 years in either direction, then by assessing whether one or more of these buffers covers each point along the timeline. By design, this step reduces intra-site oversampling bias by insistently single-counting site occupation whenever multiple buffers overlap, hence the label 'redundancy filtering through presence-absence buffering' (RFPAB). By the same token, it places what has elsewhere been labeled an 'occupation window' (Maschner et al. 2009) around the set of events represented by a site's full set of calendar age estimates, in effect presuming to fill in gaps in the sample distribution resulting from sampling error, in a manner analogous to kernel density estimation. A measure of intra-site effective or nonredundant sample size can also be derived from the area under this presence-absence time series.
2. At the scale of the study region, a TFD time series is derived by summing all sites' presence-absence time series at each point along the timeline. Temporal changes in the height of this TFD measure result from changes in the estimated count of synchronously occupied sites in the regional data set over time. Intra-site effective sample sizes calculated in the first step can also be summed to further estimate an effective or non-redundant sample size for the overall regional sample.
3. To mitigate chronometric uncertainty accompanying calibrated radiocarbon age estimation, the first two steps are repeated a large number of times through Monte Carlo (MC) simulation: a new sample of calendar age estimates is repeatedly drawn from each radiocarbon date's calibrated posterior distribution, and intra-site presence-absence and inter-site TFD time series are recalculated for each new sample of draws. The resulting ensemble of TFDs are then synthesized through averaging into a

'composite' TFD. Graphically, the ensemble of simulated TFDs can be represented with a spaghetti plot or summarized with a confidence ribbon or band, overlaid with the composite TFD.

By design, this procedure mitigates both intra-site oversampling bias (in the first step) and chronometric uncertainty (in the third step). In addition, it mitigates random sampling error to the degree that the aggregate TFD can be interpreted as a kernel density estimate (KDE). The presence-absence time series produced in the first step can validly be interpreted as a novel variant of a kernel density estimate (KDE), in which overlapping rectangular kernels are disallowed from stacking up in the manner typical of kernel density estimation. By implication, the TFD calculated in the second step can also validly be interpreted as a penalized KDE in which the nonduplicate segments of all sites' kernels are stacked up for the study region. Consequently, the composite TFD calculated in the third step contributes a new member to the broad family of composite KDEs discussed under different names by Baxter and Cool (2016), Bronk Ramsey (2017), Brown (2017), and McLaughlin (2019). We concede that in the CRFPAB case, the default 200-year buffer width is set a priori rather than adapted to the particular radiocarbon data set under analysis. As a result, this method cannot claim to optimize the mitigation of sampling error, as in the case of standard kernel density estimation procedures. Consequently, there is room for improvement on this aspect of the procedure in future methodological work, as was also true for kernel density estimation in its nascency (Jones et al. 1996).

Finally, we concede that the finite mixture approach recently introduced by Price et al. (2021; see also Brown, 2019: Chapter 4) should replace the use of both summed probability distributions (SPDs) and KDEs (composite or otherwise) as the standard of practice in modeling regional TFDs. However, in the present study the desire to explicitly inform regional-scale mixture models with site occupation models that mitigate intra-site sampling redundancy would require a hierarchical expansion on Price's single-level finite mixture approach. In the interim, we believe that the CRFPAB procedure offered and applied here suffices to serve this purpose. Formal and computational details of the CRFPAB procedure are described in Section 3 of Supplemental Materials, below.

Following the insights of Surovell and colleagues (Surovell and Brantingham, 2007; Surovell et al., 2009) and unlike the approach taken in our own previous radiocarbon TFD analyses (Fitzhugh et al., 2016, 2020; Gjesfjeld et al., 2019), we include standardized taphonomic corrections to mitigate the effects of declining archaeological preservation/visibility with greater antiquity, described above. The 'correction' function standardizes TFDs by adjusting the overall trend by a factor dependent on the rate of signal loss calculated from preserved and globally sampled volcanic tephras (Surovell et al., 2009). The 'correction' is deterministic and approximate, necessarily inflating features at the older end of the time series where samples sizes are typically smaller, and its accuracy will vary between geographical regions with different underlying rates of deposition, erosion and degradation. See below for comparisons of corrected and uncorrected series (cf. Williams, 2012, fig 7 and text). Despite these caveats, the taphonomic adjustment enables readers to view the TFD curves without undue attention to the aggregate positive trends, drawing focus instead to the deviations from those trends, which is where our primary interests lie.

Unlike the time series comparisons commonly presented in paleoclimate and paleoecological publications, radiocarbon TFDs are built on time-stamped data points (e.g., radiocarbon-anchored site occupation events or episodes). Consequently, no age model calculations, axis adjustments or curve fitting are necessary. The correspondence between TFD time series is literally one-to-one along the x-axis. This does not mean that the radiocarbon based TFD series are necessarily accurate representations of the archaeological events they are taken to represent (whether population trends and/or cultural shifts). TFD time series built on radiocarbon dates are constrained by the same issues affecting their interpretation in other contexts. Where terrestrial charcoal and other wood are used exclusively for building the TFDs (as in this analysis), the most likely issue to affect the trends the inclusion significant numbers of "old wood" dates in TFD data sets. Like most other radiocarbon paleodemographic analyses, here we assume that old wood issues do not systematically bias our results. That working assumption deserves scrutiny, however, especially for data sets from places like the Arctic where deadwood can preserve for centuries after death or in places where long-lived trees could have been used as fuel and raw materials (Anderson and Feathers, 2019; Ledger and Forbes, 2019; McGhee and Tuck, 1976). Collectively, large samples of dates (which is what radiocarbon-based TFDs are) should

reflect the antiquities of the life-histories of the underlying source forests, not specifically the antiquity of tree deaths—however closely associated those deaths were to the human use of the wood (the target date). We must assume for now that, on average, prehistoric fire-builders disproportionately used fuels from recently dead trees and shrubs because 1) prehistoric use of long-dead wood should be rare relative to use of recently dead wood in any assemblage, 2) the volume—and therefore chance of selection for dating—of later-growth wood (closer to the date of harvest) should be greater than the volume and chance of sampling earlier growth from the dead tree, 3) processing large logs or trees for domestic fires (the assumed source of most wood sampled here) would have been inconvenient compared to the use of smaller branches with age profiles closer to the death of the tree (or branch) and 4) common tree taxa around the North Pacific Rim live an average of only 100-200 years (Fitzhugh et al., 2016). Accordingly, the preponderance of dates in any large data set should derive from wood that predominantly dates close to the target event (human activity). The resulting one-sided skew of included 'old-wood' dates, randomly distributed through large data sets, should have the main effect of dampening amplitude variability in trends by pulling some of the TFD mass in the direction of greater antiquity, but the inclusion of such dates should not seriously affect the location of trend inflections.

1.3. Analyzing Proxy Population Time Series

Most archaeological analyses of TFDs are presented individually and interpreted as if they were de facto population trends to a given region. As we have seen, several factors can influence TFDs values, and while the fluctuation of population numbers in a region may be a significant or even dominant factor, trends necessarily also include the influence of other cultural, taphonomic and sampling variability. Arguably, taphonomic and sampling biases create mostly non-systematic biases ('noise') in individual time series. When comparing contemporaneous trends between different time series, we would not expect such noisy variability to generate correspondence more often than random. In principle, non-random, or statistically significant, trend correlations between neighboring series could be taken as a strong sign of an underlying process worth further investigation. Comparison of multiple TFD time series, therefore, provides a method to identify coherence between regions, while retaining the spatial discrimination that would be lost by pooling the samples into larger regional units of analysis. In the case of TFDs constructed as radiocarbon date summed probability distributions (SPDs), we know that the data are also influenced by both instrument error (on the order of decades) and calibration errors tied to variability in atmospheric production of radiocarbon. Brown (2015; see also Williams, 2012; Fitzhugh et al., 2016; Bronk Ramsey, 2017; Price et al., 2021) argues that SPD features (spikes and troughs) shorter than 100 years are most vulnerable to calibration error effects. As a result, demographic assessments of SPDs should only consider trends that persist for more than a century in interpreting both single and multiple time series. In contrast, the double-smoothing inherent in the CRFPAB procedure—resulting first from the use of presence-absence buffers or kernels, then from repeated MC recalculation and averaging across these recalculations—should mitigate the presence of such misleading high-resolution artifacts.

Visual examination of TFD time series allows us to consider the relationship between regional trends to each other and to compare them to known archaeological evidence. Approaching the series this way, before distilling the analyses to statistical generalizations, serves as a 'ground-truthing' exercise and one that we hope will allow readers to judge for themselves the integrity of the interpretations proposed. Visual trend comparisons make it possible to see the structure of the individual regional trends and provide a means to evaluate inferences from statistical simplifications. Nevertheless, the sheer number of trends and their inherent noisiness calls out for some method or mechanized procedure to evaluate the relatedness of multiple trends. After visual inspection, we then turn to what we believe is a novel spatial autocorrelation approach to formally evaluate the hypotheses presented above, which we have labeled a time iterative Moran's I (TIMI) analysis. Formal and computational details of this approach are presented in Section 4 below.

2. Data management

The raw North Pacific radiocarbon data sets are loaded for the Kurils (including only IKIP-KBP radiocarbon data), Hokkaido (based on a data set compiled by T. Katsunori), Alaska (compiled by A. Tremayne), Kodiak (based on a series of compilations by R. Mills, B. Fitzhugh, W. Brown, and others, featured in Brown, 2015), and the Aleutians (compiled by W. Brown).

```
## Northwest Pacific Rim (Hokkaido-Kurils)
allKurils = read.csv(
  file = "IKIP_KBP_data.csv",
  header = TRUE, stringsAsFactors = FALSE
  )
allHokkaidoRaw = read.csv(
  file = "Hokkaido_data_revised.csv",
  header = TRUE, stringsAsFactors = FALSE
  )

## Northeast Pacific Rim (Alaska)
AK_raw = read.csv(
  file = "AK_RCdates_2017Nov28.csv",
  header = TRUE, stringsAsFactors = FALSE
  )
allKodiak = read.csv(
  file = "Kodiak_data.csv",
  header = TRUE, stringsAsFactors = FALSE
  )
allAleutians = read.csv(
  file = "Aleutians_data.csv",
  header = TRUE, stringsAsFactors = FALSE
  )
```

2.1. Cleaning the Hokkaido data

The following chunks clean up the Hokkaido data.

```
# Combining Hokkaido region and coastal/inland setting into a new categorical variable
allHokkaidoRaw$RegionSetting = paste0(
  allHokkaidoRaw$Region,
  ", ",
  allHokkaidoRaw$CoastInland
  )

#EHokSoJind = which(allHokkaidoRaw$RegionSetting=="Eastern Hokkaido, Sea of Japan")

kable(table(allHokkaidoRaw$RegionSetting))
```

Var1	Freq
Central Hokkaido, Inland	1616
Central Hokkaido, Pacific Ocean	78
Central Hokkaido, Sea of Japan	32
Eastern Hokkaido, Inland	175

Var1	Freq
Eastern Hokkaido, Okhotsk Sea	215
Eastern Hokkaido, Pacific Ocean	143
Northern Hokkaido, Inland	66
Northern Hokkaido, Okhotsk Sea	18
Northern Hokkaido, Sea of Japan	65
Southern Hokkaido, Inland	15
Southern Hokkaido, Pacific Ocean	208
Southern Hokkaido, Sea of Japan	36
Southern Hokkaido, Tsugaru Strait	405

```
# Selecting only the variables that (might) matter
allHokkaido = subset(
  x = allHokkaidoRaw,
  select = c(
    Region, CoastInland, X...North.latitude,
    X...East.longitude, SiteRoman, SpecimenType,
    HalfLife, MeasuredAge, MeasuredAgeError,
    X.13C.permil., ConventionalAge, ConventionalAgeError,
    LabNo, RegionSetting
    )
  )
```

The next chunk recodes radiocarbon age measures as `NA` if they lack *point* measures. Note that age measures are included in either of two columns in the data set: `MeasuredAge` and `ConventionalAge`.

```
#sort(unique(allHokkaido$MeasuredAge))
naIndexes = which(
  allHokkaido$MeasuredAge == "" |
  allHokkaido$MeasuredAge == "?" |
  allHokkaido$MeasuredAge == "??" |
  allHokkaido$MeasuredAge == "????" |
  allHokkaido$MeasuredAge == "Failed" |
  allHokkaido$MeasuredAge == "Modern" |
  allHokkaido$MeasuredAge == "Older than 18000" |
  allHokkaido$MeasuredAge == "Unmeasurable"
  )

allHokkaido$MeasuredAge[naIndexes] = NA

#sort(unique(allHokkaido$MeasuredAge[-naIndexes]))
allHokkaido$MeasuredAge[which(allHokkaido$MeasuredAge == "6550-")] = 6550

allHokkaido$MeasuredAge = as.numeric(allHokkaido$MeasuredAge)

# This table compares region counts of the raw dataset
# and the dataset after recoding non-point measures as NA;
# sizes are identical because these cases aren't yet removed
# from the dataset
kable(cbind(
  table(allHokkaidoRaw$RegionSetting), table(allHokkaido$RegionSetting)
  ))
```

Central Hokkaido, Inland	1616	1616
Central Hokkaido, Pacific Ocean	78	78
Central Hokkaido, Sea of Japan	32	32
Eastern Hokkaido, Inland	175	175
Eastern Hokkaido, Okhotsk Sea	215	215
Eastern Hokkaido, Pacific Ocean	143	143
Northern Hokkaido, Inland	66	66
Northern Hokkaido, Okhotsk Sea	18	18
Northern Hokkaido, Sea of Japan	65	65
Southern Hokkaido, Inland	15	15
Southern Hokkaido, Pacific Ocean	208	208
Southern Hokkaido, Sea of Japan	36	36
Southern Hokkaido, Tsugaru Strait	405	405

```
## Cleaning up non-point conventional 14C ages
  #sort(unique(allHokkaido$ConventionalAge))

naIndexes2 = which(
  allHokkaido$ConventionalAge == "" |
  allHokkaido$ConventionalAge == "?" |
  allHokkaido$ConventionalAge == "Modern" |
  allHokkaido$ConventionalAge == ">29950" |
  allHokkaido$ConventionalAge == "Unmeasurable"
  )

allHokkaido$ConventionalAge[naIndexes2] = NA

#unique(allHokkaido$ConventionalAge[-naIndexes2])

allHokkaido$ConventionalAge = as.numeric(allHokkaido$ConventionalAge)
```

The following code chunk recodes radiocarbon errors as `NA` if they lack point values. Once again, separate columns are included in the dataset for measured and conventional radiocarbon ages.

```
#sort(unique(allHokkaido$MeasuredAgeError))

naIndexes3 = which(
  allHokkaido$MeasuredAgeError == "" |
  allHokkaido$MeasuredAgeError == "-" |
  allHokkaido$MeasuredAgeError == "?"
  )

allHokkaido$MeasuredAgeError[naIndexes3] = NA

#sort(unique(allHokkaido$MeasuredAgeError[-naIndexes3]))

allHokkaido$MeasuredAgeError = as.numeric(allHokkaido$MeasuredAgeError)

#sort(unique(allHokkaido$ConventionalAgeError))

naIndexes4 = which(
  allHokkaido$ConventionalAgeError == "" |
```

```
  allHokkaido$ConventionalAgeError == "?"
  )

allHokkaido$ConventionalAgeError[naIndexes4] = NA

#sort(unique(allHokkaido$ConventionalAgeError[-naIndexes4]))

allHokkaido$ConventionalAgeError = as.numeric(allHokkaido$ConventionalAgeError)
```

The following chunk removes observation that are missing either some point measure of radiocarbon age (measured or conventional) or some measure of error.

```
allHokkaidoTrimmed = subset(
  x = allHokkaido,
  subset =
    (is.finite(allHokkaido$MeasuredAge) | is.finite(allHokkaido$ConventionalAge)) &
    (is.finite(allHokkaido$MeasuredAgeError) | is.finite(allHokkaido$ConventionalAgeError))
  )

# This table summarizes the reduction in sample size after
# removing cases with incomplete radiocarbon age measurements.
kable(cbind(
  table(allHokkaido$RegionSetting),
  table(allHokkaidoTrimmed$RegionSetting)
  ))
```

Central Hokkaido, Inland	1616	1570
Central Hokkaido, Pacific Ocean	78	76
Central Hokkaido, Sea of Japan	32	31
Eastern Hokkaido, Inland	175	170
Eastern Hokkaido, Okhotsk Sea	215	215
Eastern Hokkaido, Pacific Ocean	143	143
Northern Hokkaido, Inland	66	64
Northern Hokkaido, Okhotsk Sea	18	18
Northern Hokkaido, Sea of Japan	65	65
Southern Hokkaido, Inland	15	15
Southern Hokkaido, Pacific Ocean	208	206
Southern Hokkaido, Sea of Japan	36	36
Southern Hokkaido, Tsugaru Strait	405	405

The following code chunk removes cases of questionable material type (unknown, animal bone, animal tooth, brass pipe, charred material, fish bone, human bone, peat, shell, soil, and unknown)

```
#sort(unique(allHokkaidoRaw$SpecimenType))

allHokkaidoTrimmed2 = subset(
  x = allHokkaidoTrimmed,
  subset =
    SpecimenType != "" &
    SpecimenType != "Animal bone" &
    SpecimenType != "Animal tooth" &
```

```
    SpecimenType != "Brass pipe" &
    SpecimenType != "C" &
    #SpecimenType != "Cahrred grass" &
    #SpecimenType != "Charcoal" &
    #SpecimenType != "Charcoal?" &
    #SpecimenType != "Charred bark" &
    SpecimenType != "Charred material" &
    SpecimenType != "Charred material (collected by flotation)" &
    SpecimenType != "Charred material attached to pottery surface" &
    SpecimenType != "Charred material attached to pottery surface (exterior surface)" &
    SpecimenType != "Charred material attached to pottery surface (interior surface)" &
    #SpecimenType != "Charred nuts" &
    #SpecimenType != "charred plant remain" &
    #SpecimenType != "Charred plant remain" &
    #SpecimenType != "Charred Sasa" &
    #SpecimenType != "Charred seed" &
    #SpecimenType != "Charred seed (nuts)" &
    #SpecimenType != "Charred seeds (six Polygonum)" &
    #SpecimenType != "Deer antler" &
    #SpecimenType != "Deer bone" &
    #SpecimenType != "Deer tooth" &
    #SpecimenType != "Drift wood" &
    SpecimenType != "Fish bone" &
    #SpecimenType != "Grass" &
    SpecimenType != "Human bone" &
    #SpecimenType != "Japanese lacker fragment" &
    #SpecimenType != "Japanese lacquer" &
    SpecimenType != "Peat" &
    #SpecimenType != "Root" &
    #SpecimenType != "Seed"  &
    SpecimenType != "Shell" &
    SpecimenType != "Soid attached to iron pan surface" &
    SpecimenType != "Soil" &
    #SpecimenType != "Texile" &
    SpecimenType != "Unknown" #&
    #SpecimenType != "Wood" &
    #SpecimenType != "Wooden artifact"
  )

# This table describes sample size reductions after cases
# with incomplete radiocarbon age measures and cases with
# questionable material type are removed.
kable(cbind(
  table(allHokkaido$RegionSetting),
  table(allHokkaidoTrimmed$RegionSetting),
  table(allHokkaidoTrimmed2$RegionSetting)
  ))
```

Central Hokkaido, Inland	1616	1570	1170
Central Hokkaido, Pacific Ocean	78	76	35
Central Hokkaido, Sea of Japan	32	31	22
Eastern Hokkaido, Inland	175	170	120
Eastern Hokkaido, Okhotsk Sea	215	215	116

Eastern Hokkaido, Pacific Ocean	143	143	133
Northern Hokkaido, Inland	66	64	40
Northern Hokkaido, Okhotsk Sea	18	18	10
Northern Hokkaido, Sea of Japan	65	65	45
Southern Hokkaido, Inland	15	15	8
Southern Hokkaido, Pacific Ocean	208	206	62
Southern Hokkaido, Sea of Japan	36	36	14
Southern Hokkaido, Tsugaru Strait	405	405	321

This code chunk removes cases whose radiocarbon age measures are either based on unknown half-lives or the Cambridge half-life

```
#sort(unique(allHokkaidoTrimmed2$HalfLife))
#table(allHokkaidoTrimmed2$HalfLife)
#par(mfrow = c(1,1))
#boxplot(formula = allHokkaidoTrimmed2$MeasuredAge~allHokkaidoTrimmed2$HalfLife)
#CharacteristicLifeLibby = 5568*-1/log(0.5)
#CharacteristicLifeCambridge = 5730*-1/log(0.5)
#5568/5730 == CharacteristicLifeLibby/CharacteristicLifeCambridge
#5568/5730

allHokkaidoTrimmed3 = subset(
  x = allHokkaidoTrimmed2,
  subset = (HalfLife == "5568")|(HalfLife == "5570")
  )
allHokkaidoTrimmed3$HalfLife = as.numeric(allHokkaidoTrimmed3$HalfLife)

# This table describes sample size reductions after cases
# with incomplete radiocarbon age measures, cases with
# questionable material type, and cases assuming unknown or
# Cambridge half-lives are removed.
kable(cbind(
  table(allHokkaido$RegionSetting),
  table(allHokkaidoTrimmed$RegionSetting),
  table(allHokkaidoTrimmed2$RegionSetting),
  table(allHokkaidoTrimmed3$RegionSetting)
  ))
```

Central Hokkaido, Inland	1616	1570	1170	1117
Central Hokkaido, Pacific Ocean	78	76	35	27
Central Hokkaido, Sea of Japan	32	31	22	19
Eastern Hokkaido, Inland	175	170	120	120
Eastern Hokkaido, Okhotsk Sea	215	215	116	106
Eastern Hokkaido, Pacific Ocean	143	143	133	132
Northern Hokkaido, Inland	66	64	40	40
Northern Hokkaido, Okhotsk Sea	18	18	10	10
Northern Hokkaido, Sea of Japan	65	65	45	33
Southern Hokkaido, Inland	15	15	8	8
Southern Hokkaido, Pacific Ocean	208	206	62	62
Southern Hokkaido, Sea of Japan	36	36	14	14
Southern Hokkaido, Tsugaru Strait	405	405	321	321

This chunk combines “measured” and “conventional” radiocarbon ages into a single column. It does the same for “measured” and “conventional” errors.

```
allHokkaidoTrimmed4 = allHokkaidoTrimmed3
  allHokkaidoTrimmed4$ConventionalAge = ifelse(
    test = is.na(allHokkaidoTrimmed3$ConventionalAge),
    yes = allHokkaidoTrimmed3$MeasuredAge,
    no = allHokkaidoTrimmed3$ConventionalAge
    )
  allHokkaidoTrimmed4$ConventionalAgeError = ifelse(
    test = is.na(allHokkaidoTrimmed3$ConventionalAgeError),
    yes = allHokkaidoTrimmed3$MeasuredAgeError,
    no = allHokkaidoTrimmed3$ConventionalAgeError
    )
#write.csv(x = allHokkaidoTrimmed4, file = "HokkaidoCleanedImputed.csv")
```

This chunk is the final prepartion of the Hokkaido/W. Pacific sample for temporal frequency analysis (TFA).

```
forTFAnalysisHokkaido = data.frame(
  LabNo = allHokkaidoTrimmed4$LabNo,
  BP = allHokkaidoTrimmed4$ConventionalAge,
  SE = allHokkaidoTrimmed4$ConventionalAgeError,
  Site = allHokkaidoTrimmed4$SiteRoman,
  RegionSetting = allHokkaidoTrimmed4$RegionSetting,
  stringsAsFactors = FALSE
  )
forTFAnalysisKurils = subset(x = allKurils, select = c(labNo, rcybp, rcError, siteName))
colnames(forTFAnalysisKurils) = c("LabNo", "BP", "SE", "Site")
```

2.2. Cleaning the Alaska data

The following chunk combines Alaska region and ecological setting/type into a new categorical variable.

```
AK_raw = AK_raw[order(AK_raw$AHRSNO, AK_raw$Site_Name, AK_raw$Normalized_C14_Date),]
AK_raw$regionRecode = paste0(AK_raw$Habitat, ", ", AK_raw$Ecological_Setting)

#write.csv(x = table(AK_raw$regionRecode, AK_raw$Material), file = "AKmatByRegion.csv")
```

The following code chunk removes Island data points from the Alaska set, including the Kodiak and Aleutian Archipelagos and St. Lawrence, Nunivak, King, and Round Islands. While most sites with Afognak (AFG) and Karluk (KAR) codes are removed as a part of the Kodiak data, two are retained because they are actually located on the Alaska Peninsula: Cabin Point Island Village (KAR-00121) and Sukoi Bay Terrace (AFG-00207). Likewise, while most XNI sites are on Nunivak, Cevnermiut (XNI-00053) and Qengaramiut (XNI-00052) are on the mainland and are retained.

```
KodiakInds = sort(unique(c(
  grep(pattern = "KOD", x = AK_raw$AHRSNO, ignore.case = TRUE),
  grep(pattern = "AFG", x = AK_raw$AHRSNO, ignore.case = TRUE),
  grep(pattern = "KAR", x = AK_raw$AHRSNO, ignore.case = TRUE),
  grep(pattern = "XTI", x = AK_raw$AHRSNO, ignore.case = TRUE)
  )))
KodiakInds = KodiakInds[-which(KodiakInds == which(AK_raw$Site_Name == "Cabin Point Island Village"))]
```

```
KodiakInds = KodiakInds[-which(KodiakInds == which(AK_raw$Site_Name == "Sukoi Bay Terrace"))]

AleutianInds = which(AK_raw$Habitat == "Aleutian Islands")

NunivakInds = sort(unique(c(
  grep(pattern = "XCM", x = AK_raw$AHRSNO, ignore.case = TRUE),
  grep(pattern = "XNI", x = AK_raw$AHRSNO, ignore.case = TRUE)
  )))
NunivakInds = NunivakInds[-which(NunivakInds == which(AK_raw$Site_Name == "Cevnermiut"))]
NunivakInds = NunivakInds[-which(NunivakInds == which(AK_raw$Site_Name == "Qengaramiut"))]

StLawrenceInds = sort(unique(c(
  grep(pattern = "XSL", x = AK_raw$AHRSNO, ignore.case = TRUE)
  )))

KingIslandInds = sort(unique(c(
  which(AK_raw$AHRSNO == "TEL-00210")
  )))

RoundIslandInds = grep(
  pattern = "Round Island",
  x = AK_raw$Site_Name, ignore.case = TRUE
  )

AK_trim1 = AK_raw[
  -sort(unique(c(
    KodiakInds,
    AleutianInds,
    NunivakInds,
    StLawrenceInds,
    KingIslandInds,
    RoundIslandInds
    ))),
  ]
```

The following code chunk maps out Alaska data mid-trim/cleaning, showing trimmed sites in red and retained sites in blue.

```
world1 = map("world", fill=TRUE, col="transparent", plot=FALSE)
world2 = map2SpatialPolygons(
  world1,
  world1$names,
  CRS("+proj=longlat +ellps=WGS84")
  )
# Removing problematic polygons
world3 = world2[-grep("Antarctica", row.names(world2)),]
world3 = world3[-grep("Ghana", row.names(world3)),]
world3 = world3[-grep("UK:Great Britain", row.names(world3)),]
world4 = nowrapRecenter(world3)

rawLongRecode = ifelse(
  AK_raw$DDLONG<0,
  AK_raw$DDLONG+360,
```

```
  AK_raw$DDLONG
  )
trim1LongRecode = ifelse(
  AK_trim1$DDLONG<0,
  AK_trim1$DDLONG+360,
  AK_trim1$DDLONG
  )

plot(
  world4,
  xlim = range(rawLongRecode,trim1LongRecode, na.rm = TRUE),
  ylim = range(AK_raw$DDLAT, AK_trim1$DDLAT, na.rm = TRUE),
  col="dark gray", border = "dark gray",bg="light gray"
  )
points(x = rawLongRecode, y = AK_raw$DDLAT, col = "red", pch=20)
points(x = trim1LongRecode, y = AK_trim1$DDLAT, col = "blue", pch=20)
```

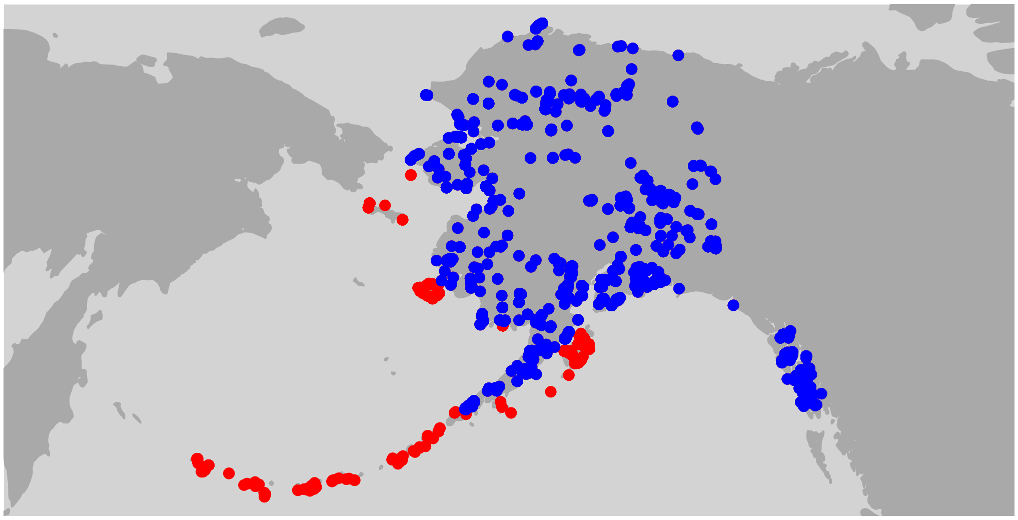

The following chunk removes data points of unreliable or unknown material. Plant matter (wood, twig, charcoal, grass, bark) and antler are retained.

```
kable(table(AK_raw$Material))
```

Var1	Freq
	9
Alkali Soluble Portion	1
antler	1
Antler	28
Ash	1
Bark	14
Bark/Grass/Twigs	1
Birch Bark	1
bone	28
Bone	167
Burned Bone	1
Calcined Bone	3
Carbonized Plant Remains	1
caribou bone collagen; collagène osseux de caribou	1
charcoal	17
Charcoal	1899
Charcoal and Bone	1
Charcoal and Peat	2
Charcoal and Snail Shells	1
Charcoal and Wood	1
Charcoal?	27
Charrded Wood	1
Charred Bone	1
Charred Log	1
Charred material	3
Charred Material	320
Charred Wood	11
Cone	1
construction material (wood?)	1
driftwood	19
Fur	6
Grass	4
Grass mat	1
Grass Mat	1
Hair	1
Hide	3
Ivory	5
Leather	1
Organic Material	1
Organic Matter	1
organic Sediment	1
Organic Sediment	11
Organic Soil	6
Peat	6
Plant Material	6
Plant remains	3
Plant Remains	11
Plant/Wood	4
Pottery Residue	6
Residue	2
Root	1
Sediment	5

Var1	Freq
Shell	1
Sinew	1
Skin and Feathers	1
Soil	1
Soil Organics	4
Soot or Ash	1
Undetermined	147
wood	3
Wood	316
Wood and Charcoal	7

```
## Marine and non-anthropogenic dates already omitted,
## as well as dates challenged by P.I.s

AK_trim2 = subset(
  x = AK_trim1,
  subset =
    Material != "Alkali Soluble Portion" &
    Material != "Animal Remains" &
    #Material != "antler" &
    #Material != "Antler" &
    Material != "ash" &
    #Material != "Bark" &
    #Material != "Bark/Grass/Twigs" &
    #Material != "Birch Bark" &
    Material != "bone" &
    Material != "Bone" &
    Material != "Burned Bone" &
    Material != "Calcined Bone" &
    #Material != "Carbonized Plant Remains" &
    #Material != "caribou bone collagen; collagène osseux de caribou" &
    #Material != "charcoal" &
    #Material != "Charcoal" &
    Material != "Charcoal and Bone" &
    Material != "Charcoal and Peat" &
    Material != "Charcoal and Snail Shells" &
    #Material != "Charcoal and Wood" &
    #Material != "Charcoal?" &
    #Material != "Charred Wood" &
    Material != "Charred Bone" &
    #Material != "Charred Log" &
    Material != "Charred material" &
    Material != "Charred Material" &
    #Material != "Charred Wood" &
    #Material != "construction material (wood?)" &
    #Material != "driftwood" &
    Material != "Fur" &
    #Material != "Grass" &
    #Material != "Grass mat" &
    #Material != "Grass Mat" &
    Material != "Hair" &
    Material != "Hide" &
```

```
    Material != "ivory" &
    Material != "Ivory" &
    Material != "Leather" &
    Material != "Organic Material" &
    Material != "Organic Matter" &
    Material != "organic Sediment" &
    Material != "Organic Sediment" &
    Material != "Organic Soil" &
    Material != "Peat" &
    #Material != "Plant Material" &
    #Material != "Plant remains" &
    #Material != "Plant Remains" &
    #Material != "Plant/Wood" &
    Material != "Pottery Residue" &
    Material != "Residue" &
    Material != "Root" &
    Material != "Sediment" &
    Material != "Shell" &
    Material != "Sinew" &
    Material != "Soil" &
    Material != "Soil Organics" &
    Material != "Soot or Ash" &
    Material != "Undetermined" #&
    #Material != "wall construction (wood?)" &
    #Material != "wood" &
    #Material != "Wood" &
    #Material != "Wood and Charcoal"
    )
```

This chunk is the final prepartion of the Alaska/E. Pacific sample for TFA.

```
forTFAnalysisAlaska = data.frame(
  LabNo = AK_trim2$Lab_NO,
  BP = AK_trim2$Normalized_C14_Date,
  SE = AK_trim2$Normalized_Error,
  Site = AK_trim2$AHRSNO,
  RegionSetting = paste0(AK_trim2$Habitat, ", ", AK_trim2$Ecological_Setting),
  stringsAsFactors = FALSE
  )

forTFAnalysisKodiak = subset(
  x = allKodiak,
  select = c(labNo, rcybp, rcyError, site)
  )
colnames(forTFAnalysisKodiak) = c("LabNo", "BP", "SE", "Site")

forTFAnalysisAleutians = subset(
  x = allAleutians,
  select = c(labNo, rcybp, rcyError, site)
  )
colnames(forTFAnalysisAleutians) = c("LabNo", "BP", "SE", "Site")
```

2.3. Packaging the Hokkaido and Alaska data for TFA

Finally, the following code chunk packages the data sets by region into `R` list objects for analysis.

```
HokkaidoRegions = sort(unique(forTFAnalysisHokkaido$RegionSetting))
listForTFAnalysisHokkaido = list()
  for (i in 1:length(HokkaidoRegions)){
    listForTFAnalysisHokkaido[[i]] = subset(
      x = forTFAnalysisHokkaido,
      subset = RegionSetting == HokkaidoRegions[i]
      )
    names(listForTFAnalysisHokkaido)[i] = HokkaidoRegions[i]
    }

AlaskaRegions = sort(unique(forTFAnalysisAlaska$RegionSetting))
listForTFAnalysisAlaska = list()
  for (i in 1:length(AlaskaRegions)){
    listForTFAnalysisAlaska[[i]] = subset(
      x = forTFAnalysisAlaska,
      subset = RegionSetting == AlaskaRegions[i]
      )
    names(listForTFAnalysisAlaska)[i] = AlaskaRegions[i]
    }
```

3. Formal and computational details of the composite redundancy filtering through presence-absence buffering (CRFPAB) procedure

3.1. Calibration

The IntCal13 and IntCal20 calibration curves are loaded into the work space in the following R code chunk. Several functions are also written:

- A function that can produce a cubic spline interpolation of calibration curves at any desired temporal grain, with a default of 5-year intervals
- A function that will calculate the likelihood of observing an inputted radiocarbon age r_i given a corresponding measurement error s_i, calendar timestamp t, and calibration curve:

$$r_i \sim \text{Normal}\left(\mu_\rho(t), s_i^2 + \sigma_\rho^2(t)\right)$$

$$\therefore p(r_i|s_i,t) = \frac{1}{\sqrt{2\pi \times (s_i^2 + \sigma_\rho^2(t))}} \times \exp\left\{-0.5\frac{(r_i - \mu_\rho(t))^2}{s_i^2 + \sigma_\rho^2(t)}\right\}$$

- A function that normalizes the likelihood for each inputted assay (i.e., calculates a Bayesian posterior for each assay assuming a flat prior and no dependence with other dates)

```
IntCal13.raw = read.table(
  file = "http://intcal.org/curves/intcal13.14c",
  skip = 11,
  sep = ","
  )[,1:3]
IntCal20.raw = read.table(
  file = "http://intcal.org/curves/intcal20.14c",
  skip = 11,
  sep = ","
  )[,1:3]

colnames(IntCal13.raw) = colnames(IntCal20.raw) =
  c("calBP", "mu_rho.t", "sigma_rho.t")

calcurve.cspline = function(temp.res = 5, calcurve = IntCal20.raw){
  timeline = seq(min(calcurve[,1]), max(calcurve[,1]), temp.res)
  cspline.interp = data.frame(
    timeline,
    spline(
      x = calcurve$calBP,
      y = calcurve$mu_rho.t,
      xout = timeline
      )$y,
    spline(
      x = calcurve$calBP,
      y = calcurve$sigma_rho.t,
      xout = timeline
      )$y
    )
  colnames(cspline.interp) = colnames(calcurve)
  return(cspline.interp)
  }
```

```r
IntCal13.5yr = calcurve.cspline(calcurve = IntCal13.raw)
IntCal20.5yr = calcurve.cspline(calcurve = IntCal20.raw)

calGivenRcLlhds = function(
  RCdates, temp.res=5, calcurve=IntCal20.raw
){
  calcurve.interp = calcurve.cspline(
    temp.res = temp.res,
    calcurve = calcurve
    )
  llhdMat = matrix(
    NA, nrow = nrow(RCdates),
    ncol = nrow(calcurve.interp)
    )
  for(i in 1:nrow(RCdates)){
    llhdMat[i,] = dnorm(
      x = RCdates[i,2],
      mean = calcurve.interp$mu_rho.t,
      sd = sqrt(calcurve.interp$sigma_rho.t^2 + RCdates[i,3]^2)
      )
    }
  rownames(llhdMat) = RCdates[,1]
  colnames(llhdMat) = calcurve.interp$calBP
  return(llhdMat)
  }

stratifiedPosts = function(
  RCdates,
  temp.res=5,
  calcurve=IntCal20.raw,
  SPD=FALSE
){
  llhdMat = calGivenRcLlhds(
    RCdates = RCdates,
    temp.res = temp.res,
    calcurve = calcurve
    )
  postsFromUnifPrior = llhdMat/(temp.res*rowSums(llhdMat))
  out = postsFromUnifPrior
  if(SPD){
    SPDout = colSums(postsFromUnifPrior)/nrow(RCdates)
    out = SPDout; names(out) = colnames(llhdMat)
    }
  return(out)
  }
```

3.2. Redundancy filtering through presence-absence buffering (RFPAB)

We wish to elicit a descriptive model of our data comprising a time series that answers the repeated, time-indexed question, "what is the number of distinct geographic locations for which we have evidence of human occupation within a certain $\pm h$ period of time t?" Here, $2h$ (the size of the interval $\pm h$) is the size/width of what will be labeled a *presence-absence buffer* (PAB). To operationalize this question and the time series it elicits, we will need to distinguish between two levels of observational unit. The first is the geographic location (e.g. the archaeological site). In the following mathematical expressions,

- j will be used as a placeholder for the index or unique identifier of a single geographic location out of J locations.
- For each distinct location j, the symbol i_j will be used as a placeholder for the index of a unique radiocarbon-dated event (i.e., a unique radiocarbon specimen), out of n_j specimens.
- $T_{i[j]}$ will denote the timestamp variable of the ith observation from location j.
- t will be used as a placeholder for a particular temporal value belonging to the domain of the variable T, i.e. belonging to the timeline $\mathcal{T}$.
- For a discrete time series, $\mathcal{T}$ will comprise a set of evenly spaced timestamps, in this case $\mathcal{T} = \{0, 5, 10, \ldots, 50000\}$ (the interval covered by the IntCal13 calibration curve, discretized at five-year intervals) or $\mathcal{T} = \{0, 5, 10, \ldots, 55000\}$ (the interval covered by the IntCal20 calibration curve, discretized at five-year intervals).

Assuming that each $T_{i[j]}$ is known exactly, the time series, denoted $RFPAB(t)$ below, is calculated as

$$RFPAB(t) = \sum_{j=1}^{J} I_j(t)$$

where the time series function $I_j(t)$ is an indicator variable equaling 1 if any evidence exists for the occupation of location j within a $\pm h$ interval around t—i.e. if any timestamp known for location j falls within the interval $t \pm h$—or 0 if none do:

$$I_j(t) = \begin{cases} 1 & \bigcup_{i[j]=1}^{n[j]} T_{i[j]} \in [t-h, t+h] \\ \\ 0 & \bigcap_{i[j]=1}^{n[j]} T_{i[j]} \notin [t-h, t+h] \end{cases}$$

The goal of the presence-absence buffer is to avoid the multiple counting of the same geographic unit for any time t. The consequence of this approach is a statistical construct—the time series $RFPAB(t)$ that is formally analogous to a kernel density estimate based on a rectangular kernel with a binwidth of h, with the caveat that repeated observations from the same location are prevented from contributing to the value of $RFPAB(t)$ to the degree that their specimen-indexed rectangular kernels overlap.

A proportional measure of nonredundant data points for each location can be calculated as

$$NR_j = \frac{\frac{5}{h} \sum_{\mathcal{T}} I_j(t)}{n_j}$$

Justification: Assuming no overlap between any pair of specimens' rectangular kernels, the contribution of each specimen's kernel to the area under the $I_j(t)$ curve would be 1. Each kernel would contribution either 0 or $1/h$ to the $I_j(t)$ function at time t. Because the timeline is discretized at five-year intervals, multiplying $1/h$ by 5 accommodates the area "missing" under each kernel between 5-year points. As the degree of overlap between kernels increases, the numerator decreases from n_j toward 1; in the unique case that all observations for a given geographic location have identical timestamps, non-redundancy would minimize at $1/n_j$. The numerator can therefore be thought of as an "effective" sample size for location j.

In a similar fashion, proportional non-redundancy can be calculated for a whole data set across all locations as

$$NR = \frac{\frac{5}{h}\sum_{\mathcal{T}} RFPAB(t)}{\sum_{j=1}^{J} n_j} = \frac{\frac{5}{h}\sum_{\mathcal{T}} RFPAB(t)}{n}$$

In the unique case that all observations for each geographic location have timestamps for all other observations from the same geographic location, this proportional non-redundancy measure would minimize at J/n. Once again, the numerator can be interpreted as an effective sample size for the whole region.

The size of h will affect the smoothness of the resulting descriptive model, with larger values of h smoothing the function to a much greater degree, analogous to the bandwidth of a kernel density estimate. The formal similarity of this statistical construct to a kernel density estimate may thus tempt us to treat the RFPAB time series as a probability density estimate, once normalized:

$$\hat{p}(t) = \frac{RFPAB(t)}{5\sum_{\mathcal{T}} RFPAB(t)}$$

That being said, few of the safeguards that have been developed over the decades for kernel density estimates are in place here. Most importantly, no rule of thumb for tuning h for this unique application has been explored, whose value will not only smooth the distribution but also increase the degree of redundancy as h increases.

3.3. Composite RFPAB (CRFPAB)

If we do not know $T_{i[j]}$ exactly for any timestamp but possess a probabilistic estimate for each, an additional step is recommended and applied here, based on Monte Carlo simulation.

- First, we draw a unique sample from each timestamp's posterior distribution:

$$T_{i[j]}^{(s)} \sim \text{Post}_{i[j]}$$

- Next, for each s out of S draws, we elicit a RFPAB time series as described in the previous section, in this case indexed for the sth simulated draw: $RFPAB^{(s)}(t)$. We can also calculate non-redundancy measures for each geographic location and for the overall data set, $NR_j^{(s)}$ and $NR^{(s)}$.
- Finally, if we imagine that the sth RFPAB time series were a probability distribution estimate (and normalize it accordingly), then the average of all RFPAB time series across all S draws would constitute a sort of predictive distribution for a future observation, incorporating our uncertainty regarding the set of all unknown timestamps included in our data into our prediction about the fundamentally uncertain value $\tilde{t}$ of the predictand:

$$\hat{p}(\tilde{t}) \triangleq CRFPAB_1(t) = \frac{\sum_{s=1}^{S} \hat{p}^{(s)}(t)}{S}$$

- Alternatively, the average of all time-indexed unnormalized RFPAB functions would yield the CRFPAB function,

$$CRFPAB_2(t) = \frac{\sum_{s=1}^{S} RFPAB^{(s)}(t)}{S}$$

  Without first normalizing RFPAB time series, averaging will result in CRFPAB time series with an area under the curve greater than 1.

The following chunk sets up code for calculating $I_j(t)$ and $RFPAB(t)$ time series from data

```
sim.I_jt = function(dates, calcurve, h = 100, S = 201){
  posteriors = stratifiedPosts(RCdates = dates, calcurve = calcurve)
  MC.sample = apply(
    X = posteriors, MARGIN = 1,
    FUN = sample, x = timeline, size = S, replace = TRUE
    )
  I_jt.mat = matrix(data = NA, nrow = nrow(calcurve), ncol = S)
  for (t in 1:nrow(I_jt.mat)){
    I_jt.mat[t,] = colSums(matrix(
      data = between(
        x = MC.sample,
        left = timeline[t]-h, right = timeline[t]+h
        ),
      byrow = TRUE, nrow = nrow(dates), ncol = S
      ))>0
    }
  return(I_jt.mat)
  }

sim.RFPAB = function(dates, calcurve, h = 100, S = 201){
  siteNames = unique(dates$Site); J = length(siteNames)

  # Creates a T-by-J-by-S array:
  # one row per time step,
  # one column per site,
  # one slice per simulation
  I_jt.siteArray = array(data = NA, dim = c(length(timeline), J, S))
  for (j in 1:J){
    tempDates = subset(x = dates, subset = Site == siteNames[j])
    I_jt.siteArray[,j,] = sim.I_jt(
      dates = tempDates, calcurve = calcurve,
      h = h, S = S
      )
    #print(noquote(paste(j,"/",J)))
    }
  return(apply(X = I_jt.siteArray, MARGIN = 3, FUN = rowSums))
  }
```

3.4. Taphonomic correction

Here, we adjust all RFPABs by applying Surovell ~~and Brantingham's~~ (2009) taphonomic correction, i.e. by rescaling the TFD by a time-dependent factor intended to control for the cumulative, time-transgressive hazard of site destruction. This correction factor takes the form of the reciprocal of the Lomax survival function:

$$RFPAB_{taph}(t) = RFPAB_{raw}(t) \times S^{-1}\left(t \mid b, c\right) = RFPAB_{raw}(t) \times \left(1 + \frac{t}{b}\right)^{c}$$

where b is the Lomax survival model's scale parameter estimated at 1788.03 and c is the model's shape parameter estimated at 1.26 by Surovell and Brantingham (2009).

3.5. Analysis

The following chunk applies simulated RFPAB models to the N. Pacific data, at the micro-regional scale. It also corrects each simulated RFPAB for taphonomy.

```
calcurve = IntCal13.5yr; timeline = calcurve[,1]
S. = 201
#S. = 5
h. = 100
correctionFactor = 1/Renext::plomax(
  q = timeline,
  scale = 1788.03, shape = 1.26,
  lower.tail = FALSE
  )

## Alaska
AlaskaCRFPAB = list()
nAlaskaRegions = length(listForTFAnalysisAlaska)
for (i in 1:length(listForTFAnalysisAlaska)){
  AlaskaCRFPAB[[i]] = list()
  AlaskaCRFPAB[[i]][[1]] = sim.RFPAB(
    dates = listForTFAnalysisAlaska[[i]],
    calcurve = calcurve,
    h = h., S = S.
    )
  AlaskaCRFPAB[[i]][[2]] = nrow(listForTFAnalysisAlaska[[i]])
  AlaskaCRFPAB[[i]][[3]] = AlaskaCRFPAB[[i]][[1]]*correctionFactor
  names(AlaskaCRFPAB)[i] = names(listForTFAnalysisAlaska)[i]
  #print(noquote(paste(i,"/", nAlaskaRegions)))
  }
AlaskaCRFPAB[[1+length(AlaskaCRFPAB)]] = list()
AlaskaCRFPAB[[length(AlaskaCRFPAB)]][[1]] = sim.RFPAB(
  dates = forTFAnalysisKodiak,
  calcurve = calcurve,
  h = h., S = S.
  )
AlaskaCRFPAB[[length(AlaskaCRFPAB)]][[2]] = nrow(forTFAnalysisKodiak)
AlaskaCRFPAB[[length(AlaskaCRFPAB)]][[3]] = AlaskaCRFPAB[[length(AlaskaCRFPAB)]][[1]]*correctionFactor
names(AlaskaCRFPAB)[length(AlaskaCRFPAB)] =
  "Kodiak Archipelago"
AlaskaCRFPAB[[1+length(AlaskaCRFPAB)]] = list()
AlaskaCRFPAB[[length(AlaskaCRFPAB)]][[1]] = sim.RFPAB(
  dates = forTFAnalysisAleutians, calcurve = calcurve,
  h = h., S = S.
  )
AlaskaCRFPAB[[length(AlaskaCRFPAB)]][[2]] = nrow(forTFAnalysisAleutians)
AlaskaCRFPAB[[length(AlaskaCRFPAB)]][[3]] = AlaskaCRFPAB[[length(AlaskaCRFPAB)]][[1]]*correctionFactor
names(AlaskaCRFPAB)[length(AlaskaCRFPAB)] =
  "Aleutian Archipelago"

## Hokkaido & Kurils
HokkaidoCRFPAB = list()
nHokkaidoRegions = length(listForTFAnalysisHokkaido)
for (i in 1:nHokkaidoRegions){
```

```
  HokkaidoCRFPAB[[i]] = list()
  HokkaidoCRFPAB[[i]][[1]] = sim.RFPAB(
    dates = listForTFAnalysisHokkaido[[i]],
    calcurve = calcurve,
    h = h., S = S.
    )
  HokkaidoCRFPAB[[i]][[2]] = nrow(listForTFAnalysisHokkaido[[i]])
  HokkaidoCRFPAB[[i]][[3]] = HokkaidoCRFPAB[[i]][[1]]*correctionFactor
  names(HokkaidoCRFPAB)[i] = names(listForTFAnalysisHokkaido)[i]
  #print(noquote(paste(i,"/", nHokkaidoRegions)))
}
HokkaidoCRFPAB[[1+length(HokkaidoCRFPAB)]] = list()
HokkaidoCRFPAB[[length(HokkaidoCRFPAB)]][[1]] = sim.RFPAB(
  dates = forTFAnalysisKurils,
  calcurve = calcurve,
  h = h., S = S.
  )
HokkaidoCRFPAB[[length(HokkaidoCRFPAB)]][[2]] = nrow(forTFAnalysisKurils)
HokkaidoCRFPAB[[length(HokkaidoCRFPAB)]][[3]] = HokkaidoCRFPAB[[length(HokkaidoCRFPAB)]][[1]]*correction[illegible]
names(HokkaidoCRFPAB)[length(HokkaidoCRFPAB)] =
  "Kuril Archipelago"
```

The following chunk aggregates the simulated RFPAB TFDs for micro-regions into larger regional TFDs.

```
## Alaska data aggregates
ChukchiArcticCoastCRFPAB = list()
ChukchiArcticCoastCRFPAB[[1]] =
  AlaskaCRFPAB$`Arctic Coast, Coast`[[1]] +
  AlaskaCRFPAB$`Chukchi Coast, Coast`[[1]]
ChukchiArcticCoastCRFPAB[[2]] =
  AlaskaCRFPAB$`Arctic Coast, Coast`[[2]] +
  AlaskaCRFPAB$`Chukchi Coast, Coast`[[2]]
ChukchiArcticCoastCRFPAB[[3]] =
  AlaskaCRFPAB$`Arctic Coast, Coast`[[3]] +
  AlaskaCRFPAB$`Chukchi Coast, Coast`[[3]]
names(ChukchiArcticCoastCRFPAB)[1:length(ChukchiArcticCoastCRFPAB)] =
  "Chukchi Arctic Coast"

BeringCoastCRFPAB = list()
BeringCoastCRFPAB[[1]] =
  AlaskaCRFPAB$`Bering Coast, Coast`[[1]] +
  AlaskaCRFPAB$`Bering Taiga, Coast`[[1]]
BeringCoastCRFPAB[[2]] =
  AlaskaCRFPAB$`Bering Coast, Coast`[[2]] +
  AlaskaCRFPAB$`Bering Taiga, Coast`[[2]]
BeringCoastCRFPAB[[3]] =
  AlaskaCRFPAB$`Bering Coast, Coast`[[3]] +
  AlaskaCRFPAB$`Bering Taiga, Coast`[[3]]
names(BeringCoastCRFPAB)[1:length(BeringCoastCRFPAB)] =
  "Bering Coast"

GulfOfAlaskaCRFPAB = list()
GulfOfAlaskaCRFPAB[[1]] =
```

```
  AlaskaCRFPAB$`Gulf of Alaska, Coast`[[1]]
GulfOfAlaskaCRFPAB[[2]] =
  AlaskaCRFPAB$`Gulf of Alaska, Coast`[[2]]
GulfOfAlaskaCRFPAB[[3]] =
  AlaskaCRFPAB$`Gulf of Alaska, Coast`[[3]]
names(GulfOfAlaskaCRFPAB)[1:length(GulfOfAlaskaCRFPAB)] =
  "Gulf of Alaska"

SEAKCoastCRFPAB = list()
SEAKCoastCRFPAB[[1]] =
  AlaskaCRFPAB$`SE AK Coast, Coast`[[1]]
SEAKCoastCRFPAB[[2]] =
  AlaskaCRFPAB$`SE AK Coast, Coast`[[2]]
SEAKCoastCRFPAB[[3]] =
  AlaskaCRFPAB$`SE AK Coast, Coast`[[3]]
names(SEAKCoastCRFPAB)[1:length(SEAKCoastCRFPAB)] =
  "Southeast Alaska Coast"

# The combined Bering-Brooks-Polar inland
BeringBrooksPolarInlandCRFPAB = list()
BeringBrooksPolarInlandCRFPAB[[1]] =
  AlaskaCRFPAB$`Bering Taiga, Interior`[[1]] +
  AlaskaCRFPAB$`Bering Tundra, Interior`[[1]] +
  AlaskaCRFPAB$`Brooks Tundra, Interior`[[1]] +
  AlaskaCRFPAB$`Polar Tundra, Interior`[[1]]
BeringBrooksPolarInlandCRFPAB[[2]] =
  AlaskaCRFPAB$`Bering Taiga, Interior`[[2]] +
  AlaskaCRFPAB$`Bering Tundra, Interior`[[2]] +
  AlaskaCRFPAB$`Brooks Tundra, Interior`[[2]] +
  AlaskaCRFPAB$`Polar Tundra, Interior`[[2]]
BeringBrooksPolarInlandCRFPAB[[3]] =
  AlaskaCRFPAB$`Bering Taiga, Interior`[[3]] +
  AlaskaCRFPAB$`Bering Tundra, Interior`[[3]] +
  AlaskaCRFPAB$`Brooks Tundra, Interior`[[3]] +
  AlaskaCRFPAB$`Polar Tundra, Interior`[[3]]
names(BeringBrooksPolarInlandCRFPAB)[1:length(BeringBrooksPolarInlandCRFPAB)] =
  "Bering Brooks Polar Inland"

# The Bering-only inland
BeringInlandCRFPAB = list()
BeringInlandCRFPAB[[1]] =
  AlaskaCRFPAB$`Bering Taiga, Interior`[[1]] +
  AlaskaCRFPAB$`Bering Tundra, Interior`[[1]]
BeringInlandCRFPAB[[2]] =
  AlaskaCRFPAB$`Bering Taiga, Interior`[[2]] +
  AlaskaCRFPAB$`Bering Tundra, Interior`[[2]]
BeringInlandCRFPAB[[3]] =
  AlaskaCRFPAB$`Bering Taiga, Interior`[[3]] +
  AlaskaCRFPAB$`Bering Tundra, Interior`[[3]]
names(BeringInlandCRFPAB)[1:length(BeringInlandCRFPAB)] =
  "Bering Inland"

# The combined Bering-Brooks-Polar inland
```

```
BrooksPolarInlandCRFPAB = list()
BrooksPolarInlandCRFPAB[[1]] =
  AlaskaCRFPAB$`Brooks Tundra, Interior`[[1]] +
  AlaskaCRFPAB$`Polar Tundra, Interior`[[1]]
BrooksPolarInlandCRFPAB[[2]] =
  AlaskaCRFPAB$`Brooks Tundra, Interior`[[2]] +
  AlaskaCRFPAB$`Polar Tundra, Interior`[[2]]
BrooksPolarInlandCRFPAB[[3]] =
  AlaskaCRFPAB$`Brooks Tundra, Interior`[[3]] +
  AlaskaCRFPAB$`Polar Tundra, Interior`[[3]]
names(BrooksPolarInlandCRFPAB)[1:length(BrooksPolarInlandCRFPAB)] =
  "Brooks Polar Inland"

InteriorSouthernInlandCRFPAB = list()
InteriorSouthernInlandCRFPAB[[1]] =
  AlaskaCRFPAB$`Coastal Rainforest, Interior`[[1]] +
  AlaskaCRFPAB$`Interior Boreal, Interior`[[1]] +
  AlaskaCRFPAB$`Mountain Transition, Interior`[[1]]
InteriorSouthernInlandCRFPAB[[2]] =
  AlaskaCRFPAB$`Coastal Rainforest, Interior`[[2]] +
  AlaskaCRFPAB$`Interior Boreal, Interior`[[2]] +
  AlaskaCRFPAB$`Mountain Transition, Interior`[[2]]
InteriorSouthernInlandCRFPAB[[3]] =
  AlaskaCRFPAB$`Coastal Rainforest, Interior`[[3]] +
  AlaskaCRFPAB$`Interior Boreal, Interior`[[3]] +
  AlaskaCRFPAB$`Mountain Transition, Interior`[[3]]
names(InteriorSouthernInlandCRFPAB)[1:length(InteriorSouthernInlandCRFPAB)] =
  "Interior Southern Inland"

KodiakCRFPAB = list()
KodiakCRFPAB[[1]] =
  AlaskaCRFPAB$`Kodiak Archipelago`[[1]]
KodiakCRFPAB[[2]] =
  AlaskaCRFPAB$`Kodiak Archipelago`[[2]]
KodiakCRFPAB[[3]] =
  AlaskaCRFPAB$`Kodiak Archipelago`[[3]]
names(KodiakCRFPAB)[1:length(KodiakCRFPAB)] =
  "Kodiak"

AleutiansCRFPAB = list()
AleutiansCRFPAB[[1]] =
  AlaskaCRFPAB$`Aleutian Archipelago`[[1]]
AleutiansCRFPAB[[2]] =
  AlaskaCRFPAB$`Aleutian Archipelago`[[2]]
AleutiansCRFPAB[[3]] =
  AlaskaCRFPAB$`Aleutian Archipelago`[[3]]
names(AleutiansCRFPAB)[1:length(AleutiansCRFPAB)] =
  "Aleutians"

AllAlaskaCRFPAB = list()
AllAlaskaCRFPAB[[1]] =
  AlaskaCRFPAB$`Arctic Coast, Coast`[[1]] +
  AlaskaCRFPAB$`Bering Coast, Coast`[[1]] +
```

```
  AlaskaCRFPAB$`Bering Taiga, Coast`[[1]] +
  AlaskaCRFPAB$`Bering Taiga, Interior`[[1]] +
  AlaskaCRFPAB$`Bering Tundra, Interior`[[1]] +
  AlaskaCRFPAB$`Brooks Tundra, Interior`[[1]] +
  AlaskaCRFPAB$`Chukchi Coast, Coast`[[1]] +
  AlaskaCRFPAB$`Coastal Rainforest, Interior`[[1]] +
  AlaskaCRFPAB$`Gulf of Alaska, Coast`[[1]] +
  AlaskaCRFPAB$`Interior Boreal, Interior`[[1]] +
  AlaskaCRFPAB$`Mountain Transition, Interior`[[1]] +
  AlaskaCRFPAB$`Polar Tundra, Interior`[[1]] +
  AlaskaCRFPAB$`SE AK Coast, Coast`[[1]] +
  AlaskaCRFPAB$`Kodiak Archipelago`[[1]] +
  AlaskaCRFPAB$`Aleutian Archipelago`[[1]]
AllAlaskaCRFPAB[[2]] =
  AlaskaCRFPAB$`Arctic Coast, Coast`[[2]] +
  AlaskaCRFPAB$`Bering Coast, Coast`[[2]] +
  AlaskaCRFPAB$`Bering Taiga, Coast`[[2]] +
  AlaskaCRFPAB$`Bering Taiga, Interior`[[2]] +
  AlaskaCRFPAB$`Bering Tundra, Interior`[[2]] +
  AlaskaCRFPAB$`Brooks Tundra, Interior`[[2]] +
  AlaskaCRFPAB$`Chukchi Coast, Coast`[[2]] +
  AlaskaCRFPAB$`Coastal Rainforest, Interior`[[2]] +
  AlaskaCRFPAB$`Gulf of Alaska, Coast`[[2]] +
  AlaskaCRFPAB$`Interior Boreal, Interior`[[2]] +
  AlaskaCRFPAB$`Mountain Transition, Interior`[[2]] +
  AlaskaCRFPAB$`Polar Tundra, Interior`[[2]] +
  AlaskaCRFPAB$`SE AK Coast, Coast`[[2]] +
  AlaskaCRFPAB$`Kodiak Archipelago`[[2]] +
  AlaskaCRFPAB$`Aleutian Archipelago`[[2]]
AllAlaskaCRFPAB[[3]] =
  AlaskaCRFPAB$`Arctic Coast, Coast`[[3]] +
  AlaskaCRFPAB$`Bering Coast, Coast`[[3]] +
  AlaskaCRFPAB$`Bering Taiga, Coast`[[3]] +
  AlaskaCRFPAB$`Bering Taiga, Interior`[[3]] +
  AlaskaCRFPAB$`Bering Tundra, Interior`[[3]] +
  AlaskaCRFPAB$`Brooks Tundra, Interior`[[3]] +
  AlaskaCRFPAB$`Chukchi Coast, Coast`[[3]] +
  AlaskaCRFPAB$`Coastal Rainforest, Interior`[[3]] +
  AlaskaCRFPAB$`Gulf of Alaska, Coast`[[3]] +
  AlaskaCRFPAB$`Interior Boreal, Interior`[[3]] +
  AlaskaCRFPAB$`Mountain Transition, Interior`[[3]] +
  AlaskaCRFPAB$`Polar Tundra, Interior`[[3]] +
  AlaskaCRFPAB$`SE AK Coast, Coast`[[3]] +
  AlaskaCRFPAB$`Kodiak Archipelago`[[3]] +
  AlaskaCRFPAB$`Aleutian Archipelago`[[3]]
names(AllAlaskaCRFPAB)[1:length(AllAlaskaCRFPAB)] =
  "Alaska"

## Hokkaido data aggregates
CentralHokkaidoInlandCRFPAB = list()
CentralHokkaidoInlandCRFPAB[[1]] =
  HokkaidoCRFPAB$`Central Hokkaido, Inland`[[1]]
CentralHokkaidoInlandCRFPAB[[2]] =
```

```
  HokkaidoCRFPAB$`Central Hokkaido, Inland`[[2]]
CentralHokkaidoInlandCRFPAB[[3]] =
  HokkaidoCRFPAB$`Central Hokkaido, Inland`[[3]]
names(CentralHokkaidoInlandCRFPAB)[1:length(CentralHokkaidoInlandCRFPAB)] =
  "central Hokkaido Inland"

HokkaidoPacificCoastCRFPAB = list()
HokkaidoPacificCoastCRFPAB[[1]] =
  HokkaidoCRFPAB$`Central Hokkaido, Pacific Ocean`[[1]] +
  HokkaidoCRFPAB$`Eastern Hokkaido, Okhotsk Sea`[[1]] +
  HokkaidoCRFPAB$`Eastern Hokkaido, Pacific Ocean`[[1]] +
  HokkaidoCRFPAB$`Southern Hokkaido, Pacific Ocean`[[1]]
HokkaidoPacificCoastCRFPAB[[2]] =
  HokkaidoCRFPAB$`Central Hokkaido, Pacific Ocean`[[2]] +
  HokkaidoCRFPAB$`Eastern Hokkaido, Okhotsk Sea`[[2]] +
  HokkaidoCRFPAB$`Eastern Hokkaido, Pacific Ocean`[[2]] +
  HokkaidoCRFPAB$`Southern Hokkaido, Pacific Ocean`[[2]]
HokkaidoPacificCoastCRFPAB[[3]] =
  HokkaidoCRFPAB$`Central Hokkaido, Pacific Ocean`[[3]] +
  HokkaidoCRFPAB$`Eastern Hokkaido, Okhotsk Sea`[[3]] +
  HokkaidoCRFPAB$`Eastern Hokkaido, Pacific Ocean`[[3]] +
  HokkaidoCRFPAB$`Southern Hokkaido, Pacific Ocean`[[3]]
names(HokkaidoPacificCoastCRFPAB)[1:length(HokkaidoPacificCoastCRFPAB)] =
  "Hokkaido Pacific Coast"

HokkaidoSeaOfJapanCoastCRFPAB = list()
HokkaidoSeaOfJapanCoastCRFPAB[[1]] =
  HokkaidoCRFPAB$`Central Hokkaido, Sea of Japan`[[1]] +
  HokkaidoCRFPAB$`Northern Hokkaido, Okhotsk Sea`[[1]] +
  HokkaidoCRFPAB$`Northern Hokkaido, Sea of Japan`[[1]] +
  HokkaidoCRFPAB$`Southern Hokkaido, Sea of Japan`[[1]]
HokkaidoSeaOfJapanCoastCRFPAB[[2]] =
  HokkaidoCRFPAB$`Central Hokkaido, Sea of Japan`[[2]] +
  HokkaidoCRFPAB$`Northern Hokkaido, Okhotsk Sea`[[2]] +
  HokkaidoCRFPAB$`Northern Hokkaido, Sea of Japan`[[2]] +
  HokkaidoCRFPAB$`Southern Hokkaido, Sea of Japan`[[2]]
HokkaidoSeaOfJapanCoastCRFPAB[[3]] =
  HokkaidoCRFPAB$`Central Hokkaido, Sea of Japan`[[3]] +
  HokkaidoCRFPAB$`Northern Hokkaido, Okhotsk Sea`[[3]] +
  HokkaidoCRFPAB$`Northern Hokkaido, Sea of Japan`[[3]] +
  HokkaidoCRFPAB$`Southern Hokkaido, Sea of Japan`[[3]]
names(HokkaidoSeaOfJapanCoastCRFPAB)[1:length(HokkaidoSeaOfJapanCoastCRFPAB)] =
  "Hokkaido Sea of Japan Coast"

NorthernHokkaidoInlandCRFPAB = list()
NorthernHokkaidoInlandCRFPAB[[1]] =
  HokkaidoCRFPAB$`Eastern Hokkaido, Inland`[[1]] +
  HokkaidoCRFPAB$`Northern Hokkaido, Inland`[[1]]
NorthernHokkaidoInlandCRFPAB[[2]] =
  HokkaidoCRFPAB$`Eastern Hokkaido, Inland`[[2]] +
  HokkaidoCRFPAB$`Northern Hokkaido, Inland`[[2]]
NorthernHokkaidoInlandCRFPAB[[3]] =
  HokkaidoCRFPAB$`Eastern Hokkaido, Inland`[[3]] +
```

```
  HokkaidoCRFPAB$`Northern Hokkaido, Inland`[[3]]
names(NorthernHokkaidoInlandCRFPAB)[1:length(NorthernHokkaidoInlandCRFPAB)] =
  "Northern Hokkaido Inland"

TsugaruStraitCRFPAB = list()
TsugaruStraitCRFPAB[[1]] =
  HokkaidoCRFPAB$`Southern Hokkaido, Tsugaru Strait`[[1]]
TsugaruStraitCRFPAB[[2]] =
  HokkaidoCRFPAB$`Southern Hokkaido, Tsugaru Strait`[[2]]
TsugaruStraitCRFPAB[[3]] =
  HokkaidoCRFPAB$`Southern Hokkaido, Tsugaru Strait`[[3]]
names(TsugaruStraitCRFPAB)[1:length(TsugaruStraitCRFPAB)] =
  "Tsugaru Strait"

#[Exclude `Southern Hokkaido, Inland` (n = 8)]

KurilCRFPAB = list()
KurilCRFPAB[[1]] =
  HokkaidoCRFPAB$`Kuril Archipelago`[[1]]
KurilCRFPAB[[2]] =
  HokkaidoCRFPAB$`Kuril Archipelago`[[2]]
KurilCRFPAB[[3]] =
  HokkaidoCRFPAB$`Kuril Archipelago`[[3]]
names(KurilCRFPAB)[1:length(KurilCRFPAB)] =
  "Kuril Archipelago"

AllHokkaidoCRFPAB = list()
AllHokkaidoCRFPAB[[1]] =
  HokkaidoCRFPAB$`Central Hokkaido, Inland`[[1]] +
  HokkaidoCRFPAB$`Central Hokkaido, Pacific Ocean`[[1]] +
  HokkaidoCRFPAB$`Central Hokkaido, Sea of Japan`[[1]] +
  HokkaidoCRFPAB$`Eastern Hokkaido, Inland`[[1]] +
  HokkaidoCRFPAB$`Eastern Hokkaido, Okhotsk Sea`[[1]] +
  HokkaidoCRFPAB$`Eastern Hokkaido, Pacific Ocean`[[1]] +
  HokkaidoCRFPAB$`Northern Hokkaido, Inland`[[1]] +
  HokkaidoCRFPAB$`Northern Hokkaido, Okhotsk Sea`[[1]] +
  HokkaidoCRFPAB$`Northern Hokkaido, Sea of Japan`[[1]] +
  HokkaidoCRFPAB$`Southern Hokkaido, Inland`[[1]] +
  HokkaidoCRFPAB$`Southern Hokkaido, Pacific Ocean`[[1]] +
  HokkaidoCRFPAB$`Southern Hokkaido, Sea of Japan`[[1]] +
  HokkaidoCRFPAB$`Southern Hokkaido, Tsugaru Strait`[[1]]
AllHokkaidoCRFPAB[[2]] =
  HokkaidoCRFPAB$`Central Hokkaido, Inland`[[2]] +
  HokkaidoCRFPAB$`Central Hokkaido, Pacific Ocean`[[2]] +
  HokkaidoCRFPAB$`Central Hokkaido, Sea of Japan`[[2]] +
  HokkaidoCRFPAB$`Eastern Hokkaido, Inland`[[2]] +
  HokkaidoCRFPAB$`Eastern Hokkaido, Okhotsk Sea`[[2]] +
  HokkaidoCRFPAB$`Eastern Hokkaido, Pacific Ocean`[[2]] +
  HokkaidoCRFPAB$`Northern Hokkaido, Inland`[[2]] +
  HokkaidoCRFPAB$`Northern Hokkaido, Okhotsk Sea`[[2]] +
  HokkaidoCRFPAB$`Northern Hokkaido, Sea of Japan`[[2]] +
  HokkaidoCRFPAB$`Southern Hokkaido, Inland`[[2]] +
  HokkaidoCRFPAB$`Southern Hokkaido, Pacific Ocean`[[2]] +
```

```
  HokkaidoCRFPAB$`Southern Hokkaido, Sea of Japan`[[2]] +
  HokkaidoCRFPAB$`Southern Hokkaido, Tsugaru Strait`[[2]]
AllHokkaidoCRFPAB[[3]] =
  HokkaidoCRFPAB$`Central Hokkaido, Inland`[[3]] +
  HokkaidoCRFPAB$`Central Hokkaido, Pacific Ocean`[[3]] +
  HokkaidoCRFPAB$`Central Hokkaido, Sea of Japan`[[3]] +
  HokkaidoCRFPAB$`Eastern Hokkaido, Inland`[[3]] +
  HokkaidoCRFPAB$`Eastern Hokkaido, Okhotsk Sea`[[3]] +
  HokkaidoCRFPAB$`Eastern Hokkaido, Pacific Ocean`[[3]] +
  HokkaidoCRFPAB$`Northern Hokkaido, Inland`[[3]] +
  HokkaidoCRFPAB$`Northern Hokkaido, Okhotsk Sea`[[3]] +
  HokkaidoCRFPAB$`Northern Hokkaido, Sea of Japan`[[3]] +
  HokkaidoCRFPAB$`Southern Hokkaido, Inland`[[3]] +
  HokkaidoCRFPAB$`Southern Hokkaido, Pacific Ocean`[[3]] +
  HokkaidoCRFPAB$`Southern Hokkaido, Sea of Japan`[[3]] +
  HokkaidoCRFPAB$`Southern Hokkaido, Tsugaru Strait`[[3]]
names(AllHokkaidoCRFPAB)[1:length(AllHokkaidoCRFPAB)] =
  "Hokkaido"
```

The following chunk programs two functions, one for extracting raw and effective sample sizes from the above regional RFPAB objects, the other for plotting RFPABs and CRFPABs based on these.

```
sampSizeCalc = function(CRFPAB.list, compositeOnly=TRUE){
  n_raw = CRFPAB.list[[2]]
  n_eff = colSums(CRFPAB.list[[1]])*5/(2*h.)
  out = rbind(n_raw=n_raw, n_eff=n_eff, Nonredundancy=n_eff/n_raw)
  colnames(out) = paste0("s", 1:ncol(out))
  out = cbind(
    out, Mean = c(
      CRFPAB.list[[2]],
      sum(rowMeans(CRFPAB.list[[1]]))*5/(2*h.),
      sum(rowMeans(CRFPAB.list[[1]]))*5/(2*h.)/CRFPAB.list[[2]]
      )
    )
  if(compositeOnly) out=out[,ncol(out)]
  return(out)
  }

RFPAB.plotter = function(RFPAB.obj,xlim = NULL){
  if(is.null(xlim)) xlim = rev(range(timeline))
  plot(
    x = NA,
    xlim = xlim, ylim = c(0, max(RFPAB.obj[[3]])),
    type = "l",
    xlab = "T (cal BP)", ylab = "RFPAB(t)",
    main = names(RFPAB.obj[1])
    )
  polygon(
    x = c(timeline, rev(timeline)),
    y = c(
      apply(
        X = RFPAB.obj[[1]], MARGIN = 1,
        FUN = quantile, prob = 0.025
```

```
      ),
      rev(apply(
        X = RFPAB.obj[[1]], MARGIN = 1,
        FUN = quantile, prob = 0.975
        ))
      ), col = "light gray", border = NA
    )
  lines(
    x = timeline,
    y = rowMeans(RFPAB.obj[[1]])
    )
  polygon(
    x = c(timeline, rev(timeline)),
    y = c(
      apply(
        X = RFPAB.obj[[3]], MARGIN = 1,
        FUN = quantile, prob = 0.025
        ),
      rev(apply(
        X = RFPAB.obj[[3]], MARGIN = 1,
        FUN = quantile, prob = 0.975
        ))
      ),
    col = adjustcolor("#1f78b4", alpha.f = 0.5),
    #col = "dark gray",
    border = NA
    )
  lines(
    x = timeline,
    y = rowMeans(RFPAB.obj[[3]]),
    col = "#1f78b4"
    )
  legend(
    "topleft", bty = "n",
    legend = c(
      paste(
        "n =",
        RFPAB.obj[[2]]
        ),
      paste(
        "n_eff =",
        round(sum(rowMeans(RFPAB.obj[[1]]))*5/(2*h.), 2)
        )
      )
    )
  }
```

The following chunk presents sample sizes and (C)RFPAB-based TFDs for the regions aggregated above.

```
xlims=c(10000, 0)
sampSizeCalc(ChukchiArcticCoastCRFPAB)
```

```
##          n_raw          n_eff Nonredundancy
```

```
##   324.0000000   198.6267413     0.6130455
```

```
RFPAB.plotter(RFPAB.obj = ChukchiArcticCoastCRFPAB, xlim = xlims)
```

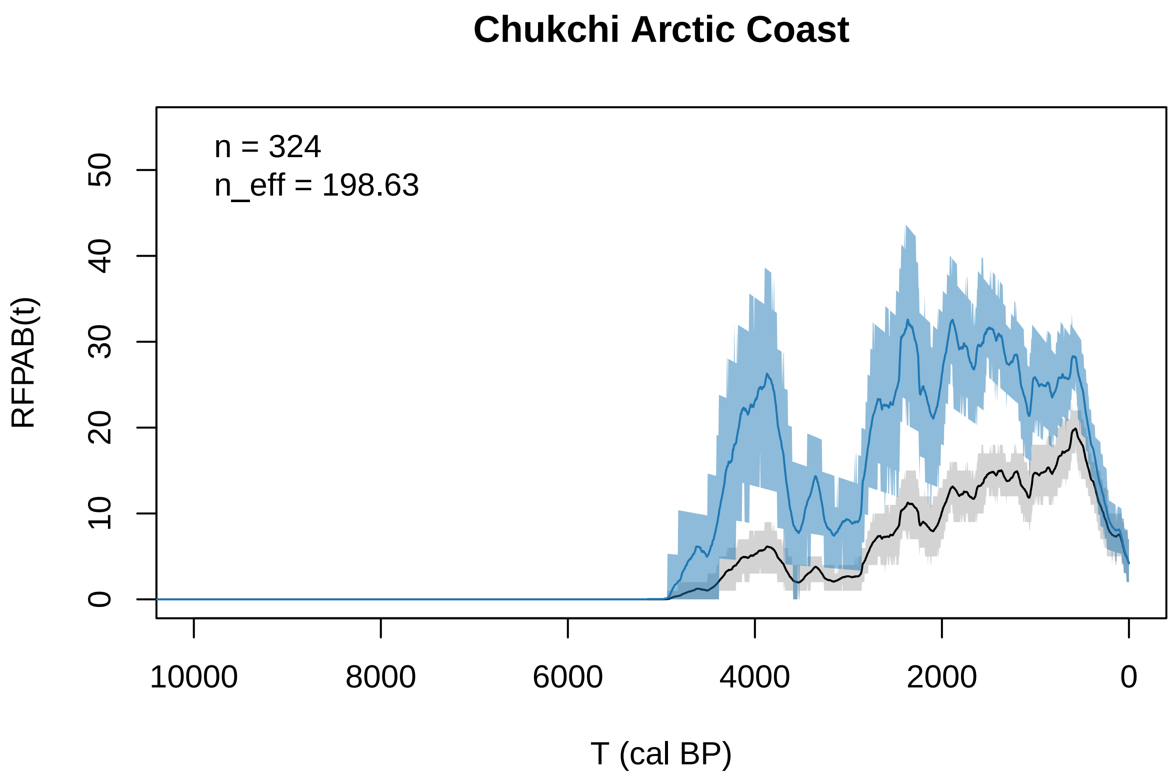


```
sampSizeCalc(BeringCoastCRFPAB)
```

```
##         n_raw         n_eff Nonredundancy
##   162.0000000   108.8370647     0.6718337
```

```
RFPAB.plotter(RFPAB.obj = BeringCoastCRFPAB, xlim = xlims)
```

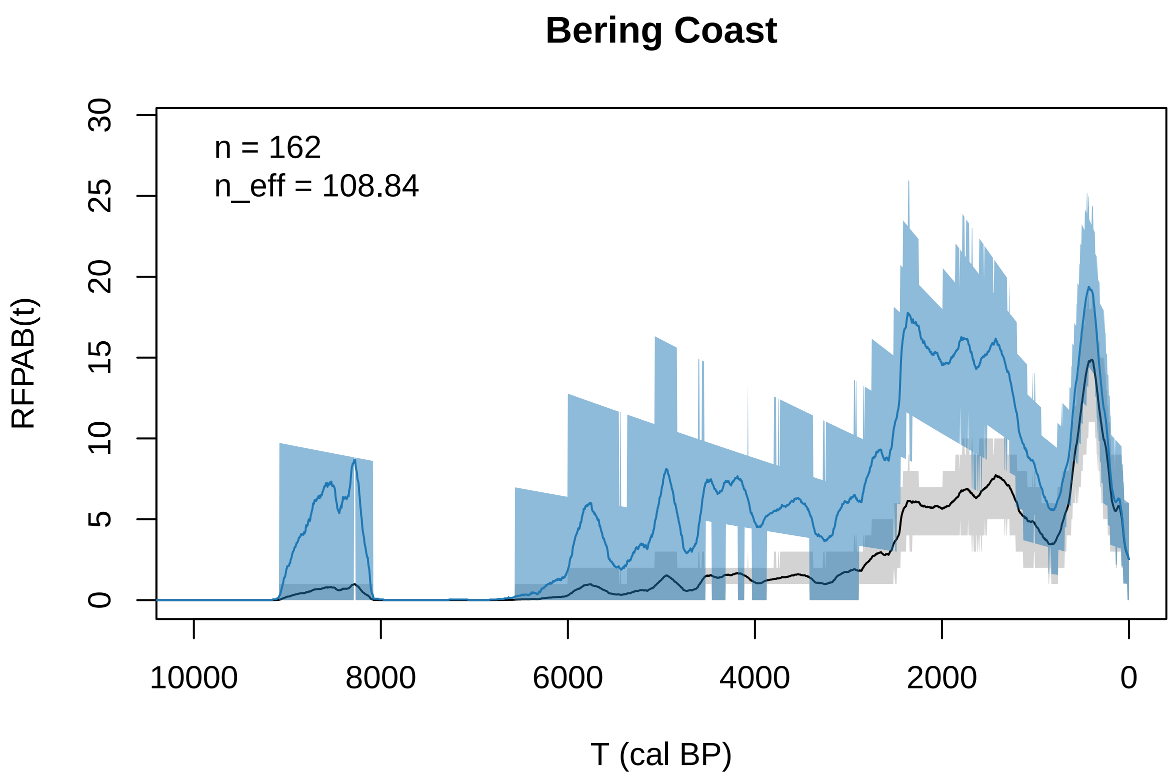


```
sampSizeCalc(GulfOfAlaskaCRFPAB)
```

```
##        n_raw        n_eff Nonredundancy
##   307.0000000   227.4279851     0.7408078
```

```
RFPAB.plotter(RFPAB.obj = GulfOfAlaskaCRFPAB, xlim = xlims)
```

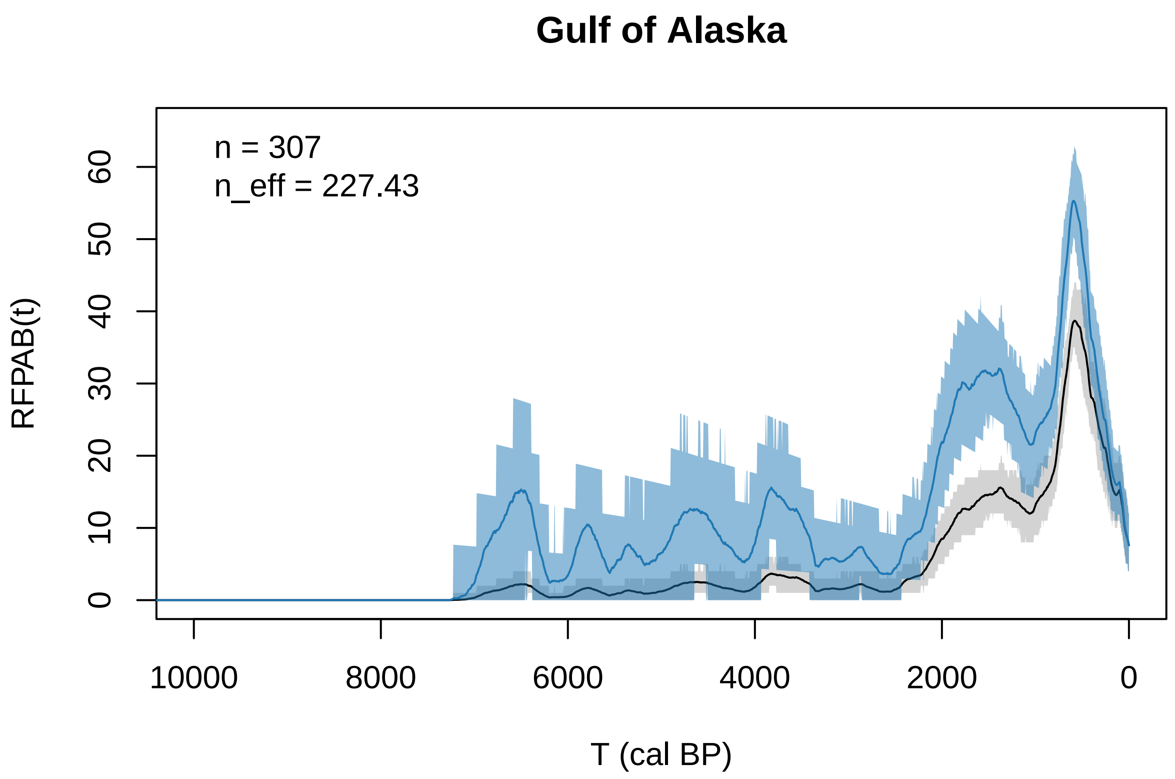


```
sampSizeCalc(SEAKCoastCRFPAB)
```

```
##          n_raw          n_eff Nonredundancy
##    211.0000000    177.5263682     0.8413572
```

```
RFPAB.plotter(RFPAB.obj = SEAKCoastCRFPAB, xlim = xlims)
```

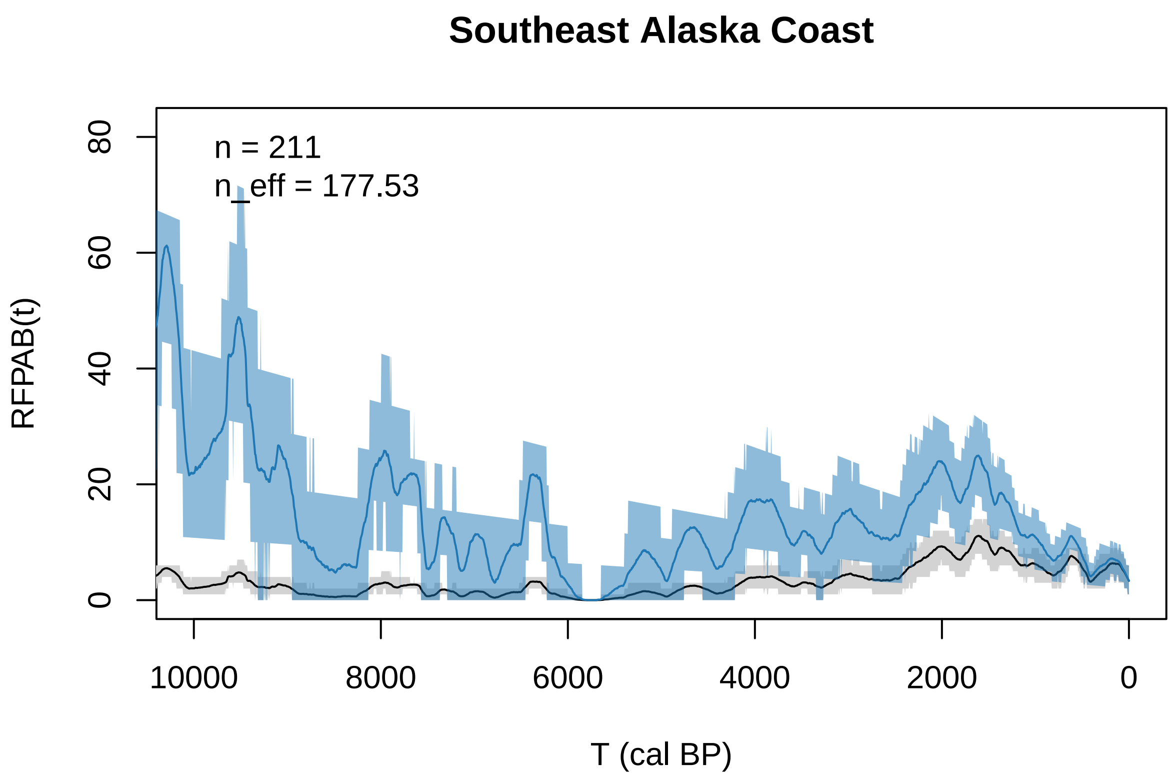


```
sampSizeCalc(BeringBrooksPolarInlandCRFPAB)
```

```
##          n_raw          n_eff Nonredundancy
##    470.0000000    304.0157960     0.6468421
```

```
RFPAB.plotter(RFPAB.obj = BeringBrooksPolarInlandCRFPAB, xlim = xlims)
```

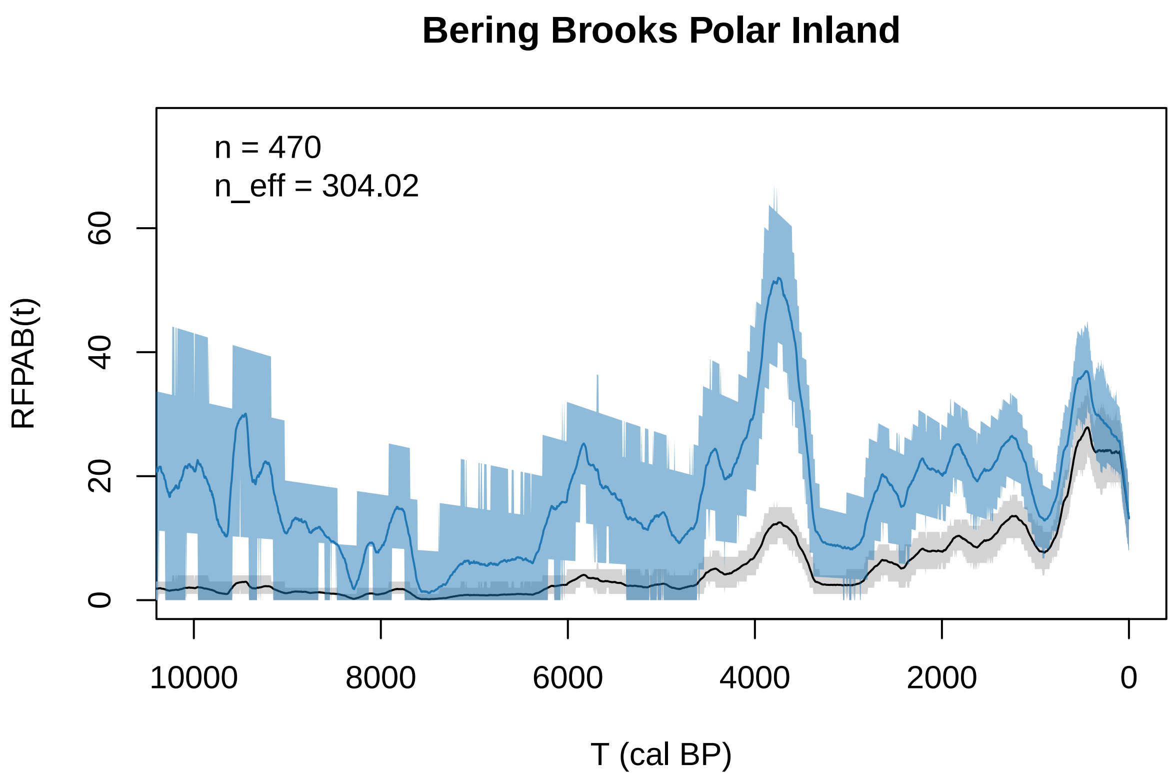


```
sampSizeCalc(BeringInlandCRFPAB)
```

```
##        n_raw        n_eff Nonredundancy
##    192.00000    121.36704       0.63212
```

```
RFPAB.plotter(RFPAB.obj = BeringInlandCRFPAB, xlim = xlims)
```

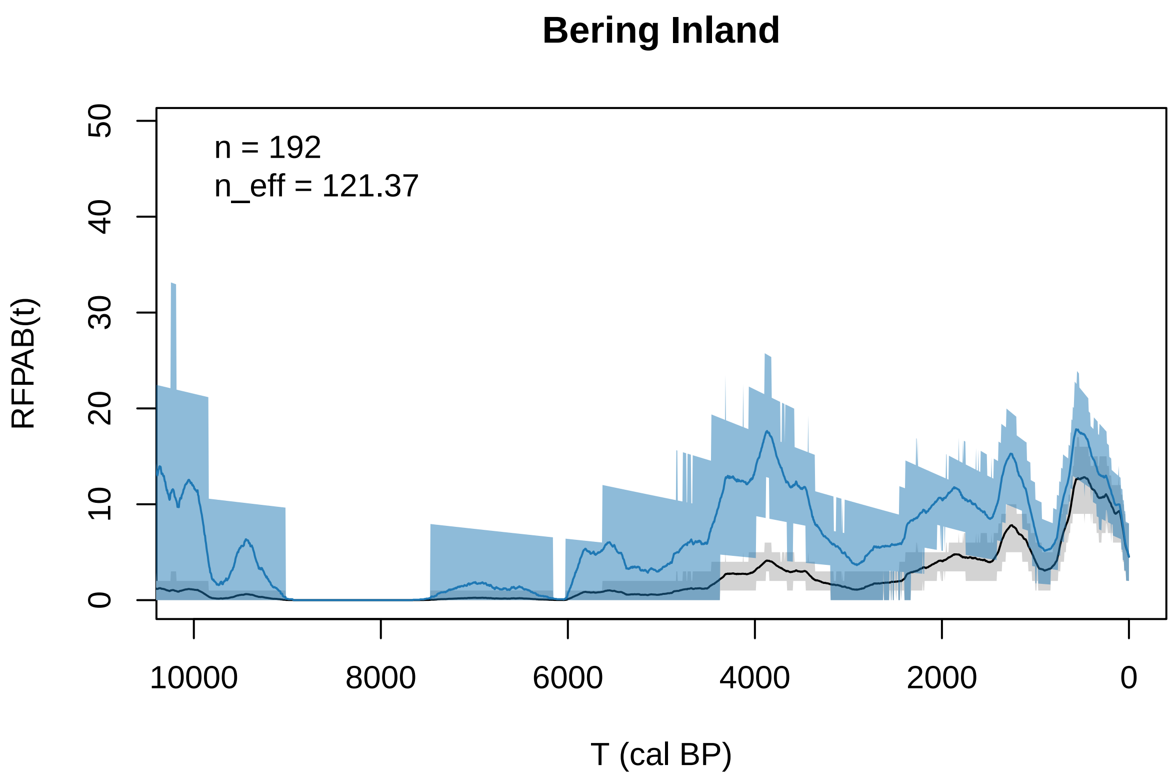


```
sampSizeCalc(BrooksPolarInlandCRFPAB)
```

```
##          n_raw          n_eff Nonredundancy
##    278.0000000    182.6487562     0.6570099
```

```
RFPAB.plotter(RFPAB.obj = BrooksPolarInlandCRFPAB, xlim = xlims)
```

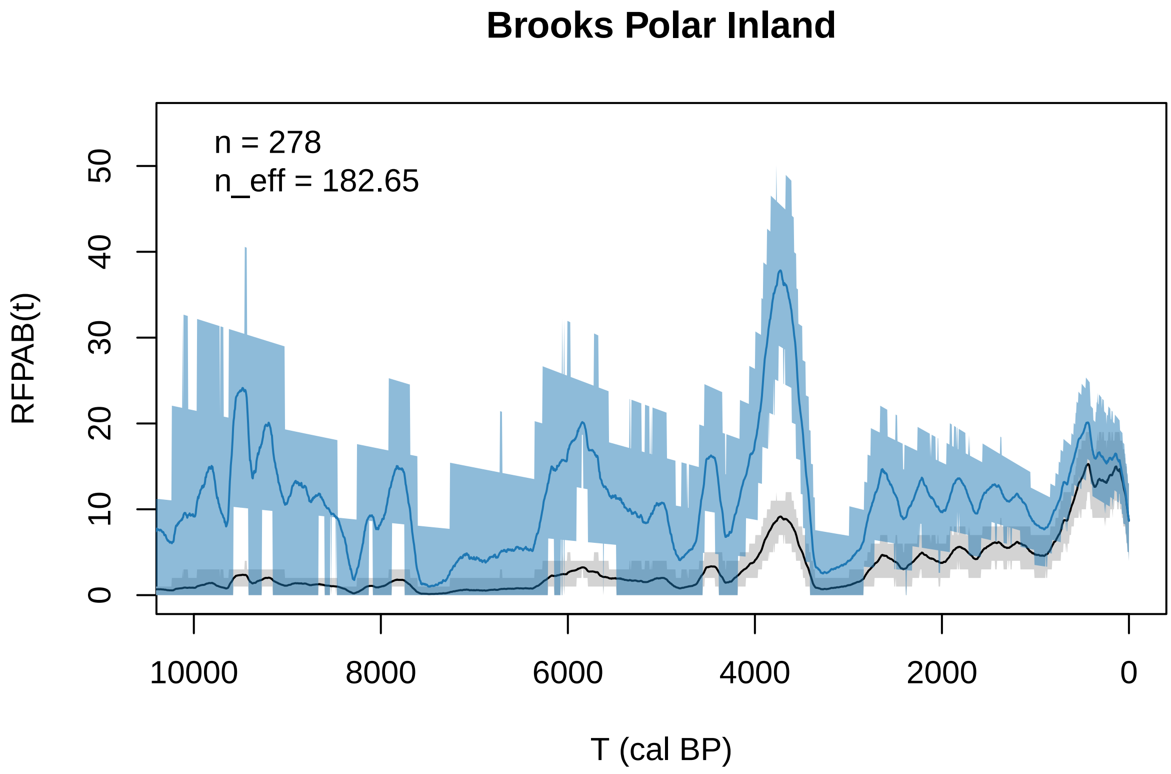


```
sampSizeCalc(InteriorSouthernInlandCRFPAB)
```

```
##          n_raw          n_eff Nonredundancy
##    522.0000000    410.1287313     0.7856872
```

```
RFPAB.plotter(RFPAB.obj = InteriorSouthernInlandCRFPAB, xlim = xlims)
```

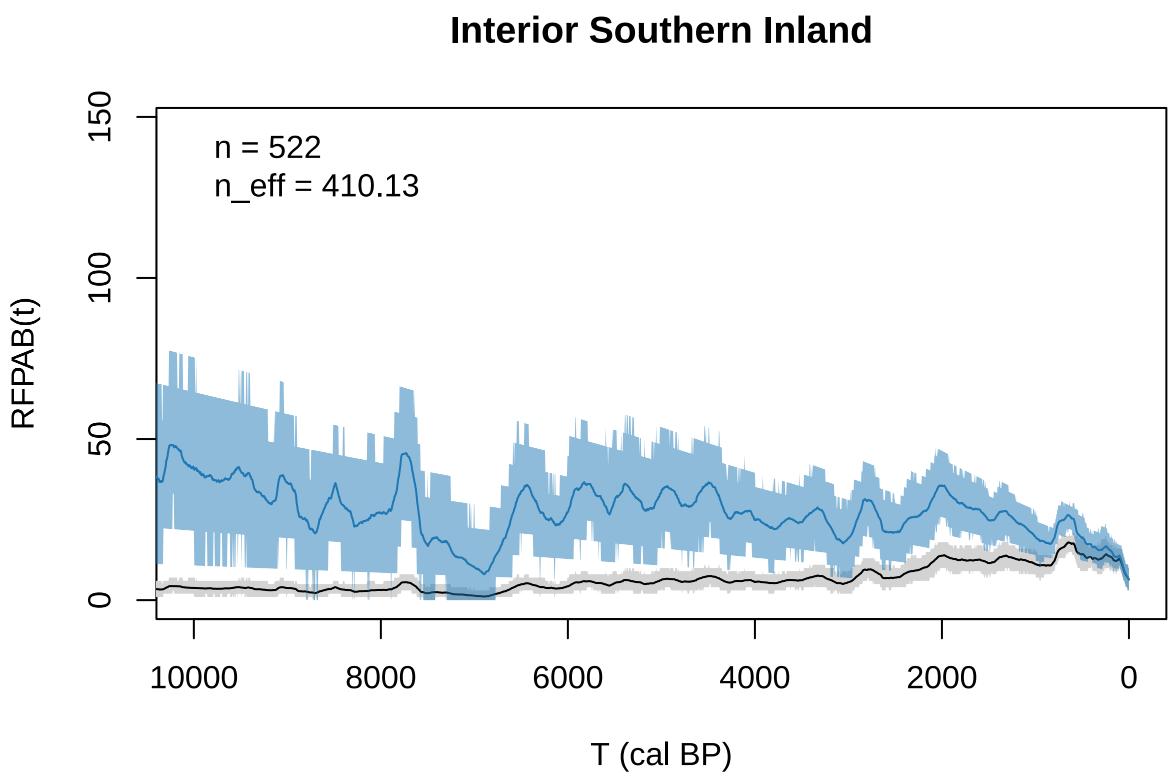


```
sampSizeCalc(KodiakCRFPAB)
```

```
##         n_raw         n_eff Nonredundancy
##   304.0000000   205.1840796     0.6749476
```

```
RFPAB.plotter(RFPAB.obj = KodiakCRFPAB, xlim = xlims)
```

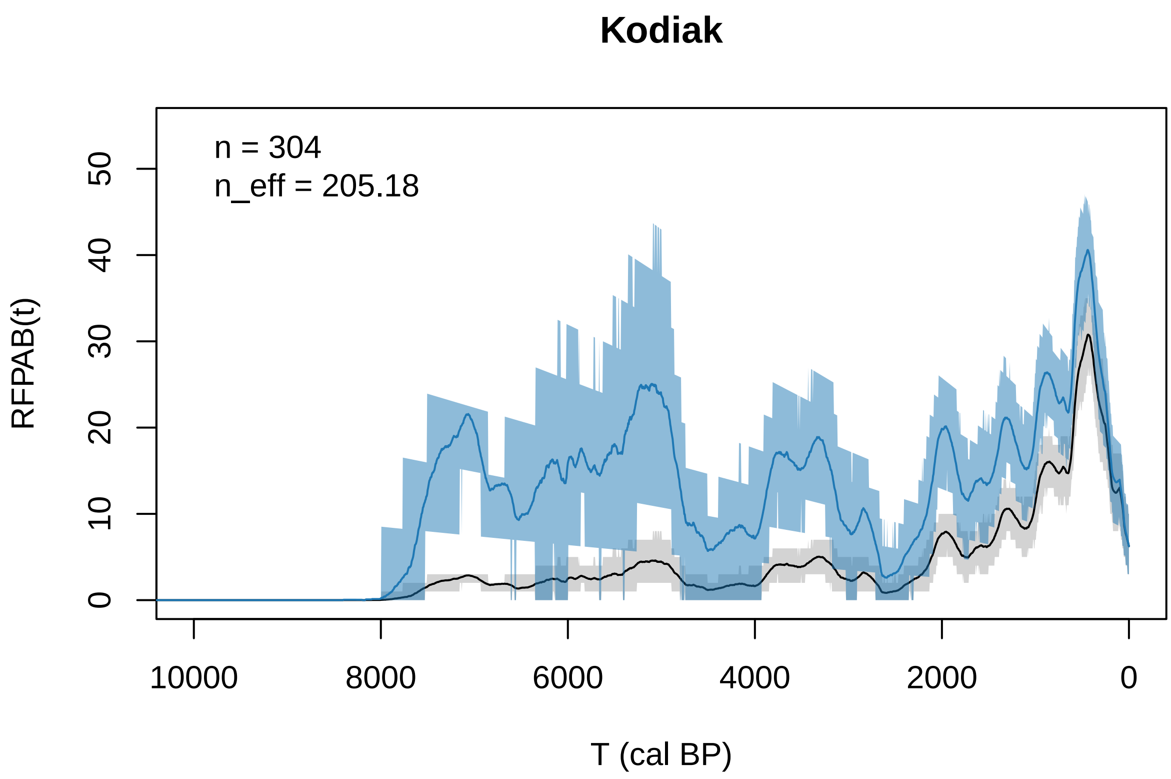


```
sampSizeCalc(AleutiansCRFPAB)
```

```
##         n_raw          n_eff Nonredundancy
##   350.0000000    238.9997512     0.6828564
```

```
RFPAB.plotter(RFPAB.obj = AleutiansCRFPAB, xlim = xlims)
```

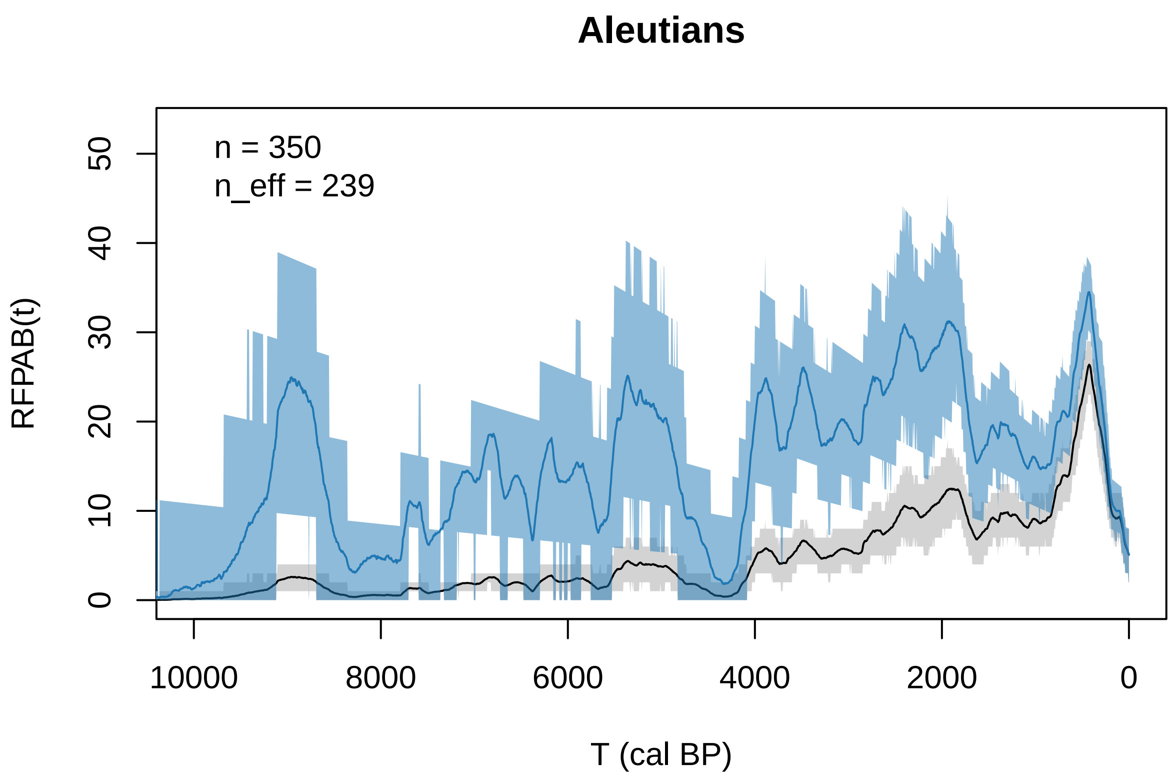


```
sampSizeCalc(AllAlaskaCRFPAB)
```

```
##          n_raw          n_eff Nonredundancy
##   2650.0000000   1870.7465174     0.7059421
```

```
RFPAB.plotter(RFPAB.obj = AllAlaskaCRFPAB, xlim = xlims)
```

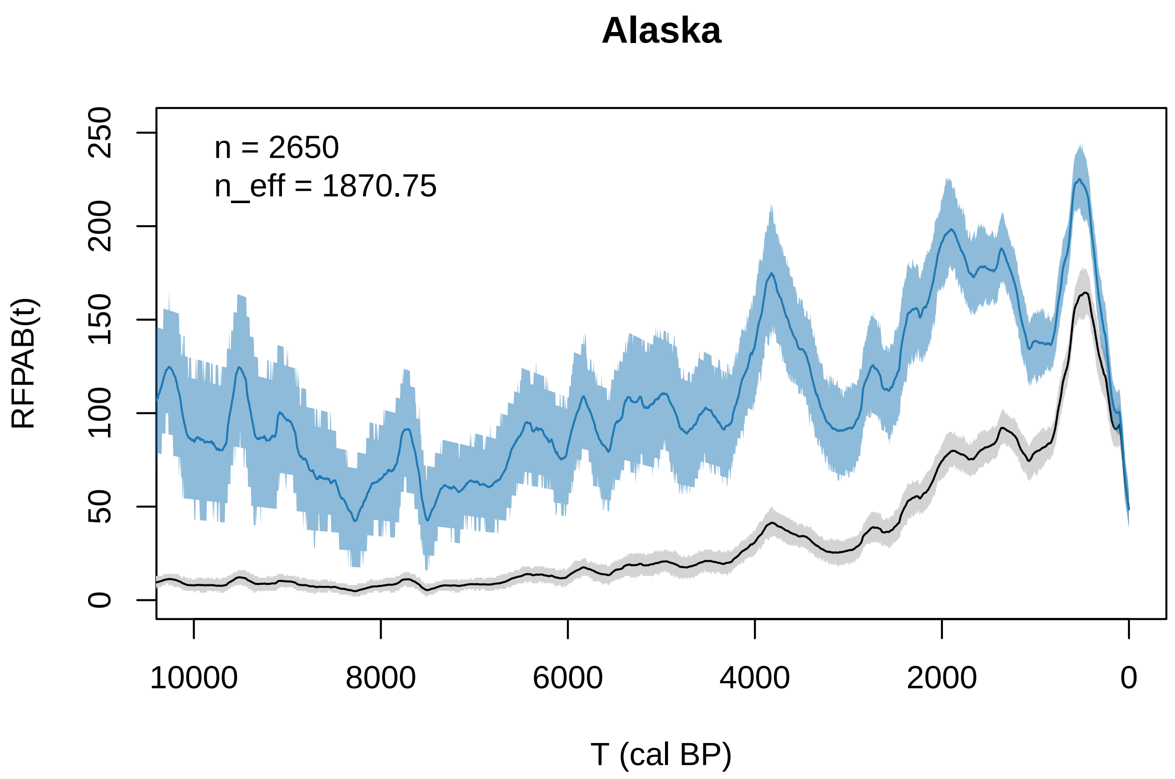


```
sampSizeCalc(CentralHokkaidoInlandCRFPAB)
```

```
##          n_raw          n_eff Nonredundancy
##   1117.0000000    437.2960199     0.3914915
```

```
RFPAB.plotter(RFPAB.obj = CentralHokkaidoInlandCRFPAB, xlim = xlims)
```

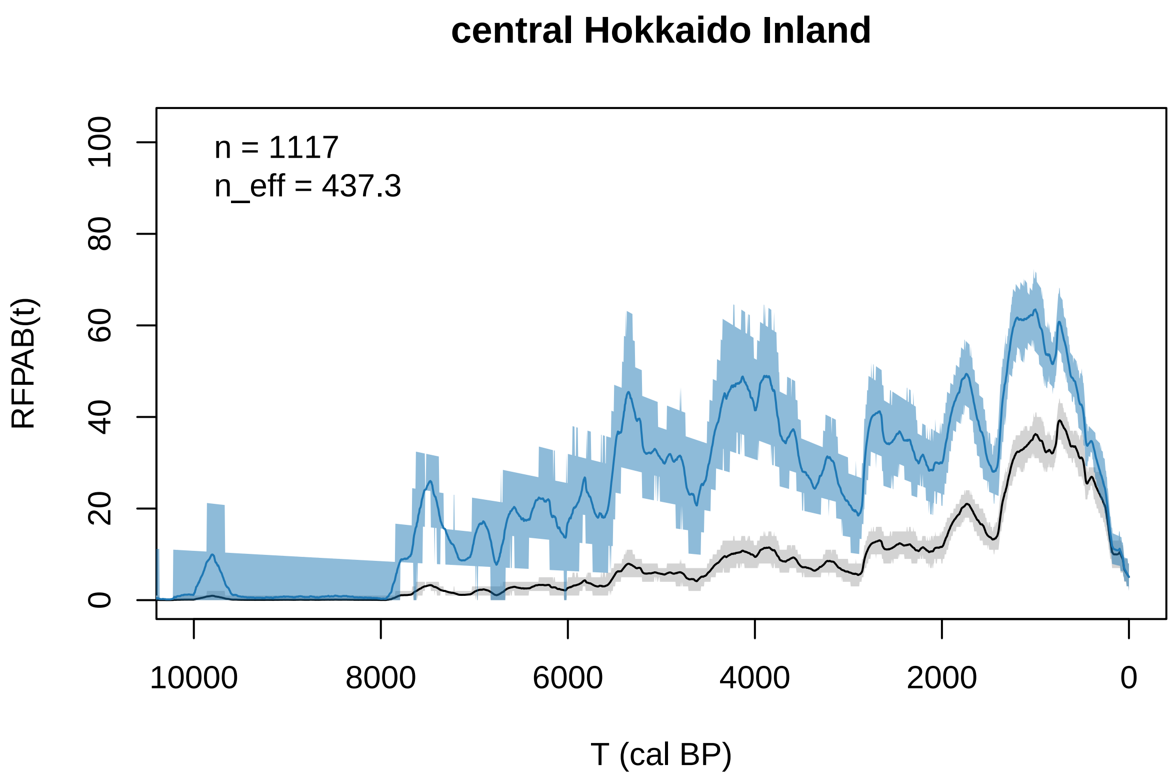


```
sampSizeCalc(HokkaidoPacificCoastCRFPAB)
```

```
##         n_raw         n_eff Nonredundancy
##   327.0000000   159.6756219     0.4883047
```

```
RFPAB.plotter(RFPAB.obj = HokkaidoPacificCoastCRFPAB, xlim = xlims)
```

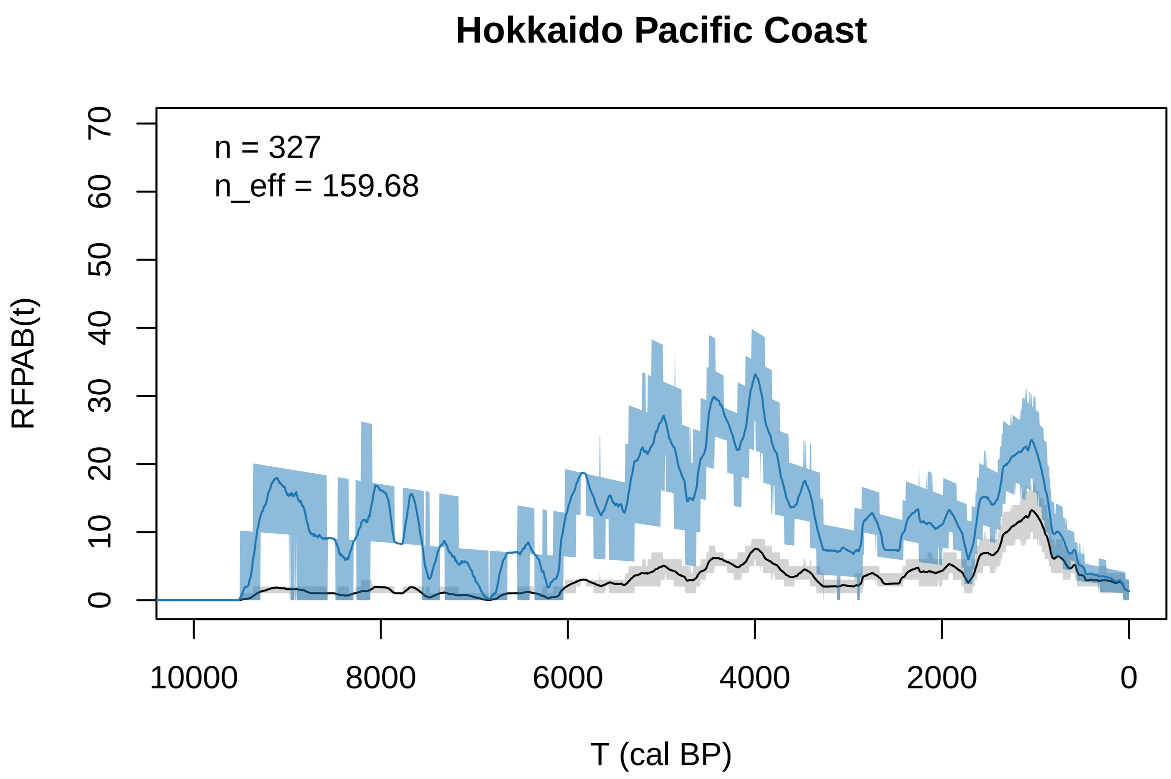


```r
sampSizeCalc(HokkaidoSeaOfJapanCoastCRFPAB)
```

```
##         n_raw          n_eff Nonredundancy
##    76.0000000     36.1284826     0.4753748
```

```r
RFPAB.plotter(RFPAB.obj = HokkaidoSeaOfJapanCoastCRFPAB, xlim = xlims)
```

Hokkaido Sea of Japan Coast

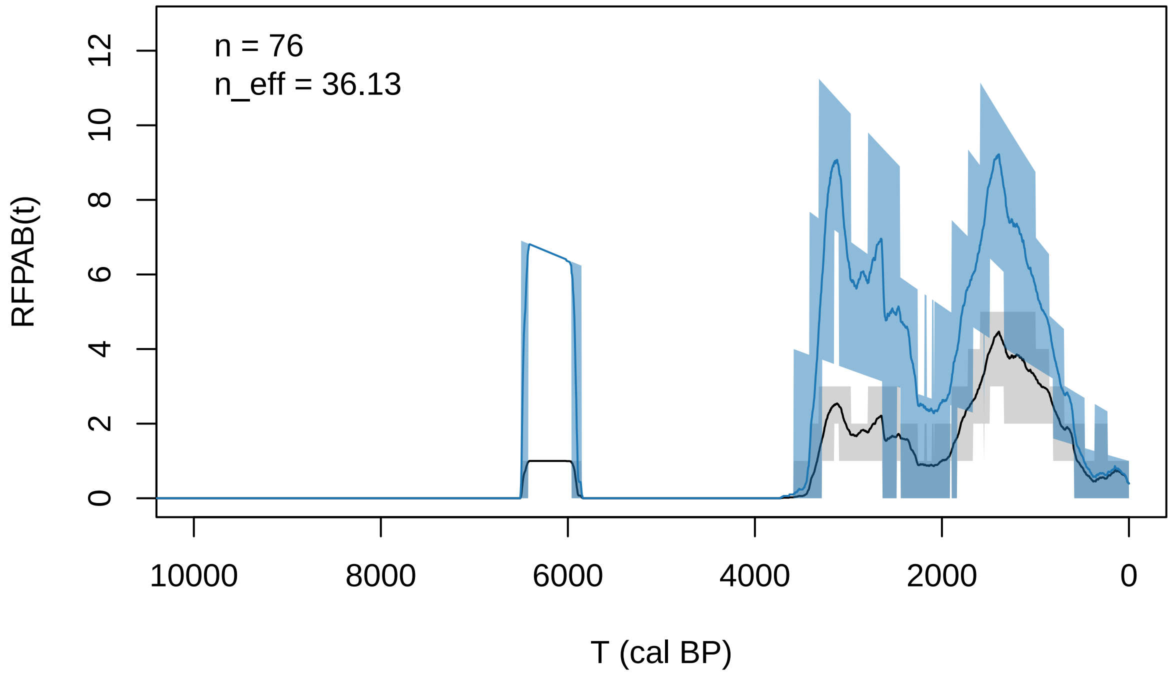


```
sampSizeCalc(NorthernHokkaidoInlandCRFPAB)
```

```
##          n_raw          n_eff Nonredundancy
##    160.0000000    123.9590796     0.7747442
```

```
RFPAB.plotter(RFPAB.obj = NorthernHokkaidoInlandCRFPAB, xlim = xlims)
```

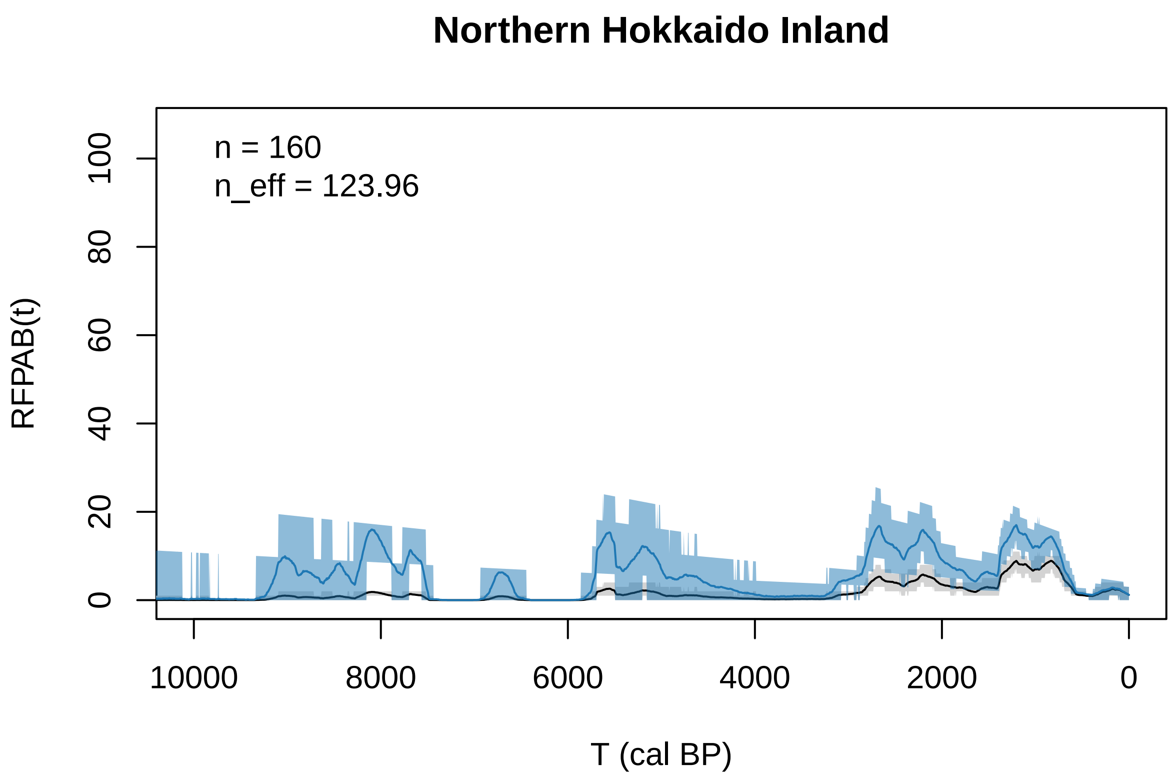


```
sampSizeCalc(TsugaruStraitCRFPAB)
```

```
##          n_raw          n_eff Nonredundancy
##    321.0000000    125.9381841     0.3923308
```

```
RFPAB.plotter(RFPAB.obj = TsugaruStraitCRFPAB, xlim = xlims)
```

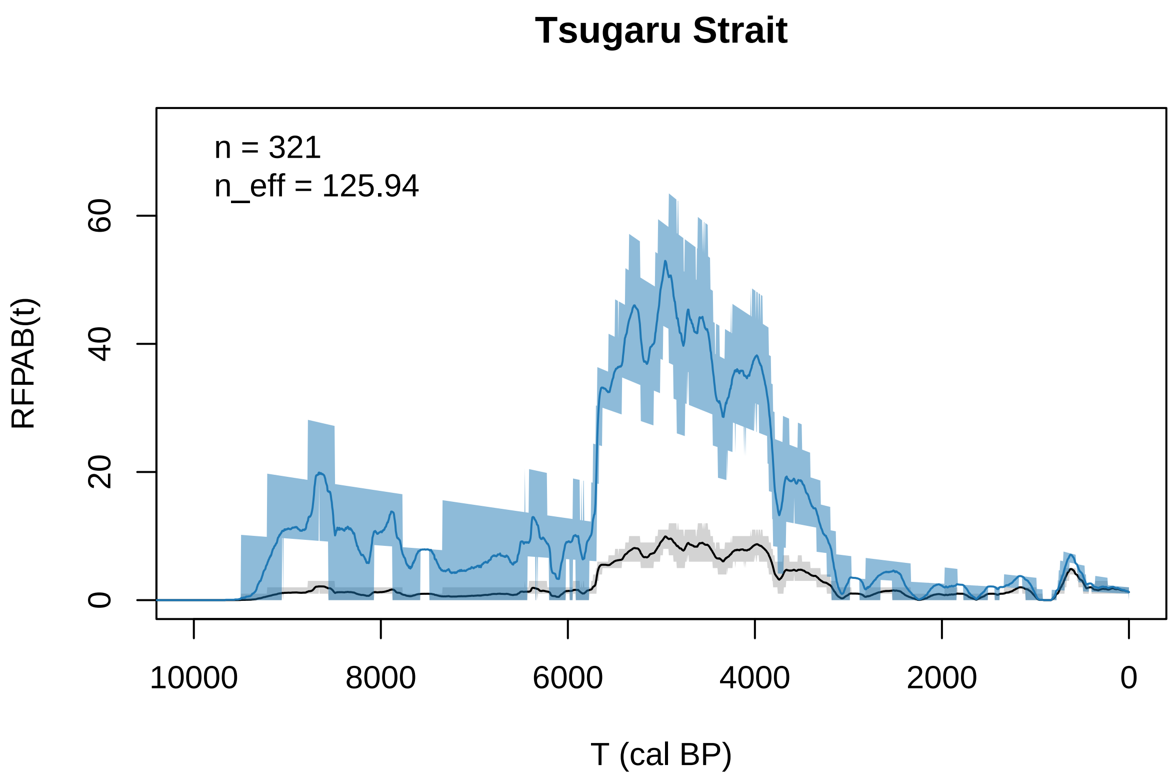


```
sampSizeCalc(KurilCRFPAB)
```

```
##         n_raw         n_eff Nonredundancy
##   364.0000000   182.2447761     0.5006725
```

```
RFPAB.plotter(RFPAB.obj = KurilCRFPAB, xlim = xlims)
```

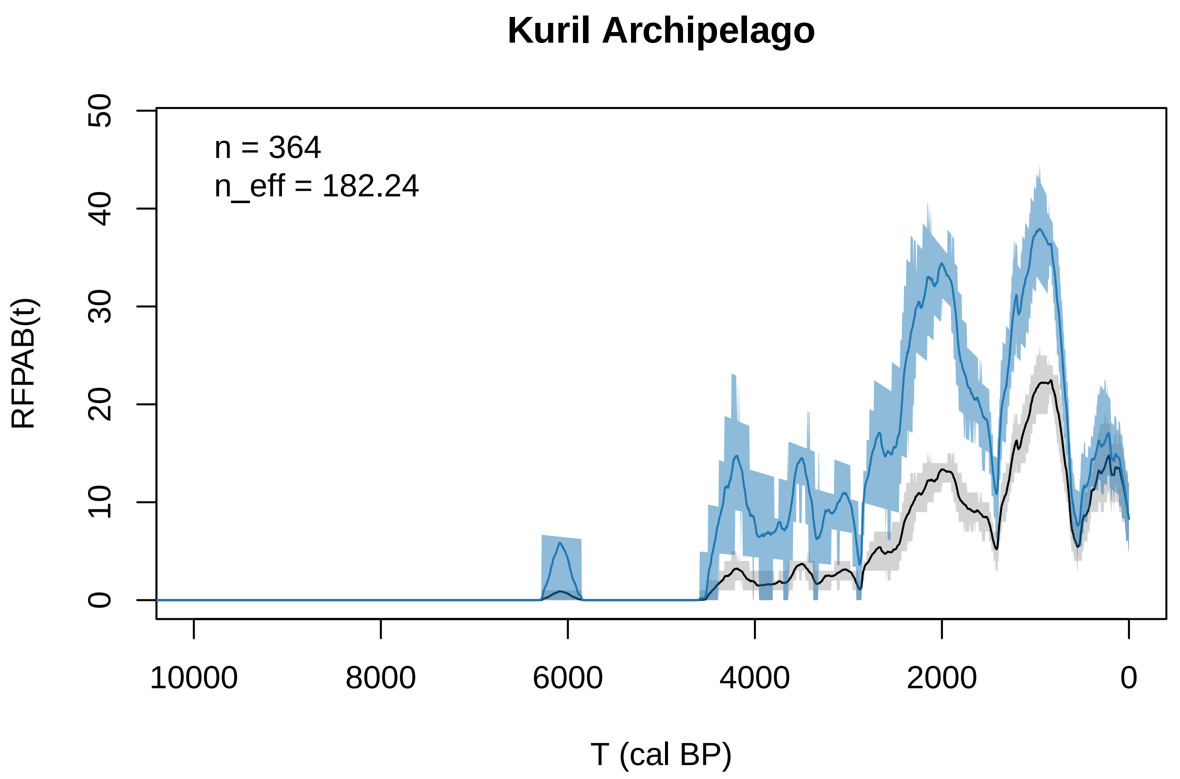


```
sampSizeCalc(AllHokkaidoCRFPAB)
```

```
##          n_raw          n_eff Nonredundancy
##   2009.0000000    890.5488806     0.4432797
```

```
RFPAB.plotter(RFPAB.obj = AllHokkaidoCRFPAB, xlim = xlims)
```

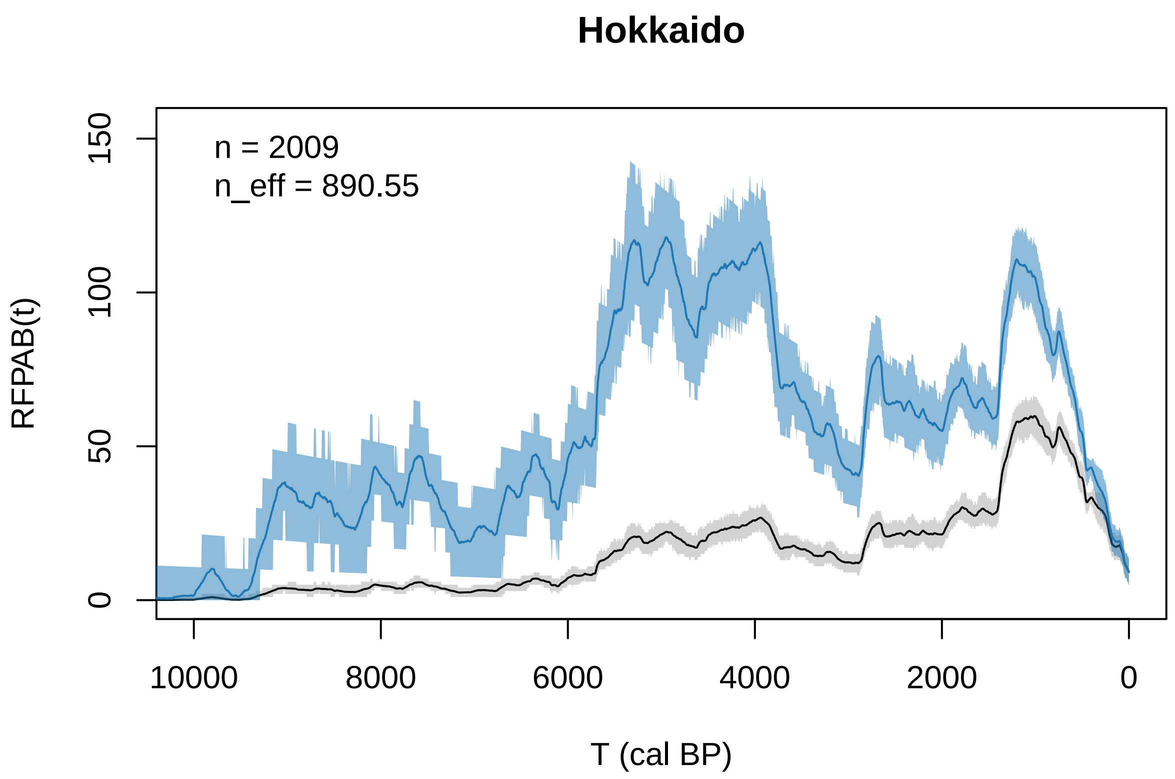


```
## Warning in rug(x = seq(5000, 0, -1000), ticksize = 0.03, col = "light gray"):
## some values will be clipped
```

The following chunk programs a function for generating stacked plots of (C)RFPABs.

```
tfdStackPlot = function(tfdList, xlim = c(50000, 0), h, corrected=TRUE, text=TRUE){
  nPanels = length(tfdList)
  tfdStackLayout = layout(
    mat = matrix(data = 1:nPanels, nrow = nPanels, ncol = 1)
    )
  corr.ind=ifelse(corrected, 3, 1)
  par(oma = c(5,5,5,5))
  for (i in 1:nPanels){
    ciEnvelope = apply(
      X = tfdList[[i]][[corr.ind]], MARGIN = 1,
      FUN = quantile, probs = c(0.025, 0.5, 0.975)
      )
    meanTfd = rowMeans(tfdList[[i]][[corr.ind]])
    est.n_eff = round(quantile(
      colSums(tfdList[[i]][[1]])*5/(2*h.),probs=c(0.025,0.975)
      ),2)
    xlimInds = which(timeline==xlim[1]):which(timeline==xlim[2])
    ylim = c(0, ceiling(max(tfdList[[i]][[corr.ind]][xlimInds,])))
    par(mar = c(0,1,0,1))
    plot(
```

```
      x = NA,
      xlim = xlim, ylim = ylim,
      xaxt = "n", yaxt = "n",
      xlab = NA, ylab = NA
      )
    abline(v = seq(50000, 0, -500), col = "light gray", lty = 2)
    abline(h = 0, lty = 3)
    polygon(
      x = c(timeline, rev(timeline)),
      y = c(ciEnvelope[1,], rev(ciEnvelope[3,])),
      col = NA, border = "dark gray", lty = 1
      )
    #lines(
    #  x = timeline,
    #  y = ciEnvelope[2,],
    #  lwd = 1, col = "black"
    #  )
    lines(
      x = timeline,
      y = meanTfd,
      lwd = 1, col = "black"
      )
    if(text){
      axis(ifelse(i %% 2 == 1, 2, 4))
        if (i == 1) axis(3)
        if (i == nPanels) axis(1)
      text(
        x = xlim[1], y = 0.8*ylim[2], pos = 4,
        font = 2,
        labels = paste0(
          names(tfdList)[i],
          ", n=",
          tfdList[[i]][[2]],
          "\n(",
          est.n_eff[1],
          " < n_eff < ",
          est.n_eff[2],
          ")"
          )
        )
      title(xlab = "t (cal BP)", outer = TRUE)
      title(ylab = "RFPAB(t)", outer = TRUE)
      }
    }
  }
```

The next several chunks generate stacked plots for various regional series of RFPABs.

```
AlaskaCRFPABlist1 = list(
  ChukchiArcticCoastCRFPAB,
  BeringCoastCRFPAB,
  AleutiansCRFPAB,
  KodiakCRFPAB,
```

```
  GulfOfAlaskaCRFPAB
  )
names(AlaskaCRFPABlist1) = c(
  "Chukchi and Arctic Coasts",
  "Bering Coast",
  "Aleutian Archipelago",
  "Kodiak Archipelago",
  "Gulf of Alaska"
  )

tfdStackPlot(tfdList = AlaskaCRFPABlist1, xlim = c(15000, 0), h = h., text = FALSE)
```

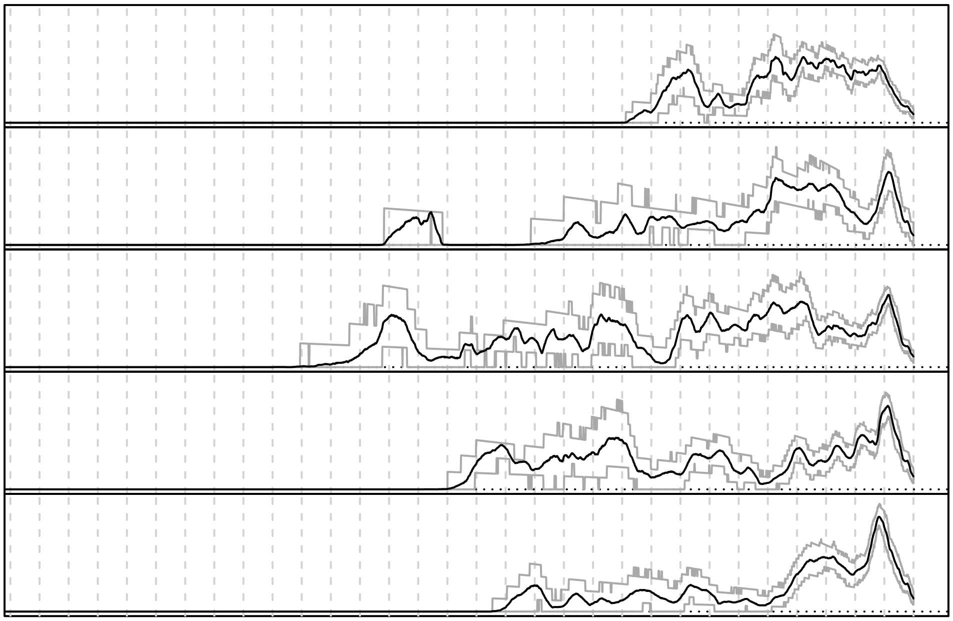

```
AlaskaCRFPABlist2 = list(
  BeringInlandCRFPAB,
  BrooksPolarInlandCRFPAB,
  InteriorSouthernInlandCRFPAB,
  SEAKCoastCRFPAB
  )
names(AlaskaCRFPABlist2) = c(
  "Bering Inland",
  "Brooks and Polar Inland",
  "Interior Boreal Forest, Mountain Transition, and Coastal Rainforest",
  "Southeast Alaska"
  )
```

```
tfdStackPlot(tfdList = AlaskaCRFPABlist2, xlim = c(15000, 0), h = h., text = FALSE)
```

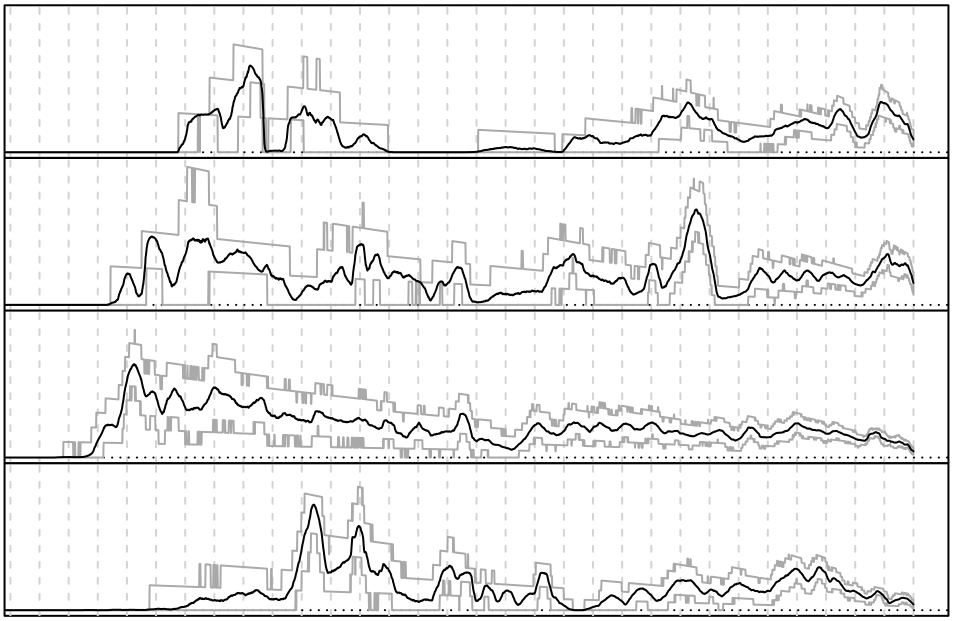

```
HokkaidoCRFPABlist = list(
  KurilCRFPAB,
  HokkaidoPacificCoastCRFPAB,
  HokkaidoSeaOfJapanCoastCRFPAB,
  NorthernHokkaidoInlandCRFPAB,
  CentralHokkaidoInlandCRFPAB,
  TsugaruStraitCRFPAB
  )
names(HokkaidoCRFPABlist) = c(
  "Kuril Archipelago",
  "Hokkaido Pacific Coast",
  "Hokkaido Sea of Japan Coast",
  "Northern Hokkaido Inland",
  "Central Hokkaido Inland",
  "Hokkaido Tsugaru Strait"
  )

tfdStackPlot(tfdList = HokkaidoCRFPABlist, xlim = c(15000, 0), h = h., text = FALSE)
```

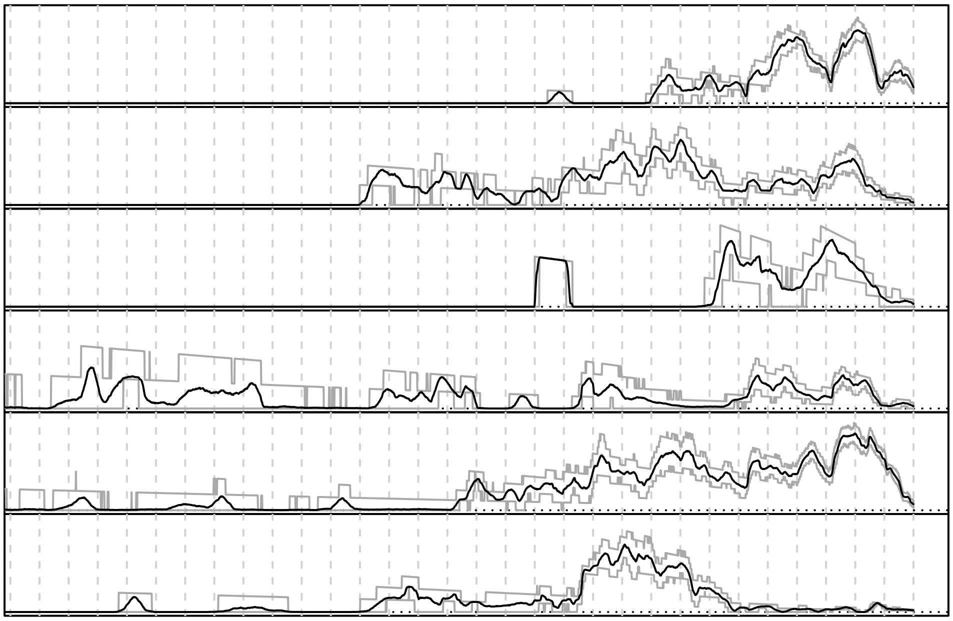

```r
AllSeriesCRFPABlist = list(
  ChukchiArcticCoastCRFPAB,
  BrooksPolarInlandCRFPAB,
  BeringInlandCRFPAB,
  BeringCoastCRFPAB,
  InteriorSouthernInlandCRFPAB,
  AleutiansCRFPAB,
  GulfOfAlaskaCRFPAB,
  KodiakCRFPAB,
  KurilCRFPAB,
  HokkaidoPacificCoastCRFPAB,
  HokkaidoSeaOfJapanCoastCRFPAB,
  NorthernHokkaidoInlandCRFPAB,
  CentralHokkaidoInlandCRFPAB,
  TsugaruStraitCRFPAB
  )
names(AllSeriesCRFPABlist) = c(
  "Arctic Coast",
  "Brooks Arctic Inland",
  "Bering Inland",
  "Bering Coast",
  "Forested Interior",
  "Aleutians",
  "Gulf of Alaska",
  "Kodiak",
  "Kuril Islands",
```

```
  "E. Hokkaido",
  "N/W Hokkaido",
  "N. Int. Hokkaido",
  "Central Int. Hokkaido",
  "Oshima"
  )

tfdStackPlot(tfdList = AllSeriesCRFPABlist, xlim = c(10000, 0), h = h., text=FALSE)
```

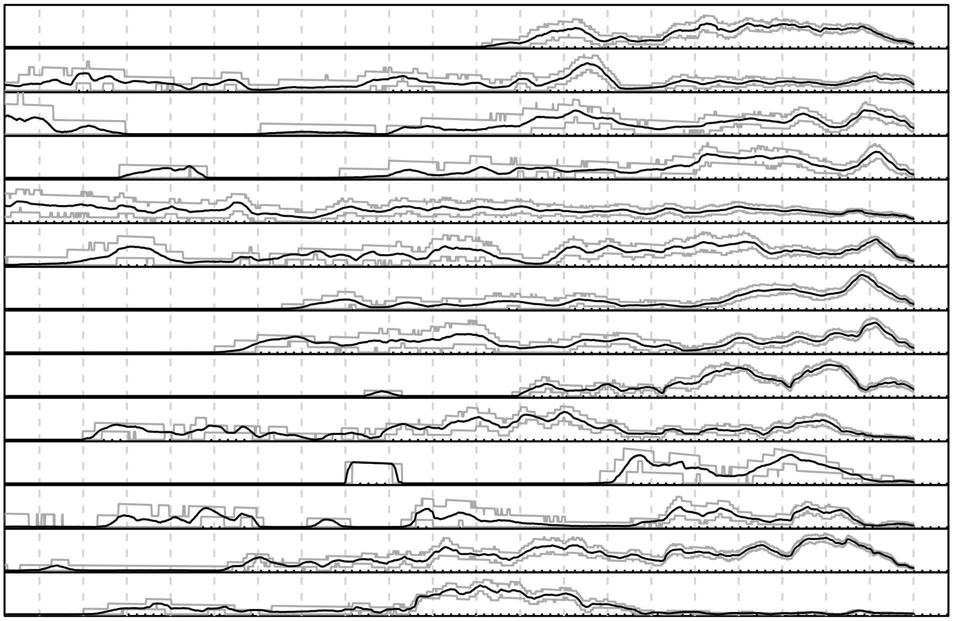

```
HokkKurilsCRFPABlist = list(
  KurilCRFPAB,
  HokkaidoPacificCoastCRFPAB,
  HokkaidoSeaOfJapanCoastCRFPAB,
  NorthernHokkaidoInlandCRFPAB,
  CentralHokkaidoInlandCRFPAB,
  TsugaruStraitCRFPAB
  )
names(HokkKurilsCRFPABlist) = c(
  "Kuril Islands",
  "E. Hokkaido",
  "N/W Hokkaido",
  "N. Int. Hokkaido",
  "Central Int. Hokkaido",
  "Oshima"
  )
```

```
tfdStackPlot(tfdList = HokkKurilsCRFPABlist, xlim = c(10000, 0), h = h., text=FALSE)
```

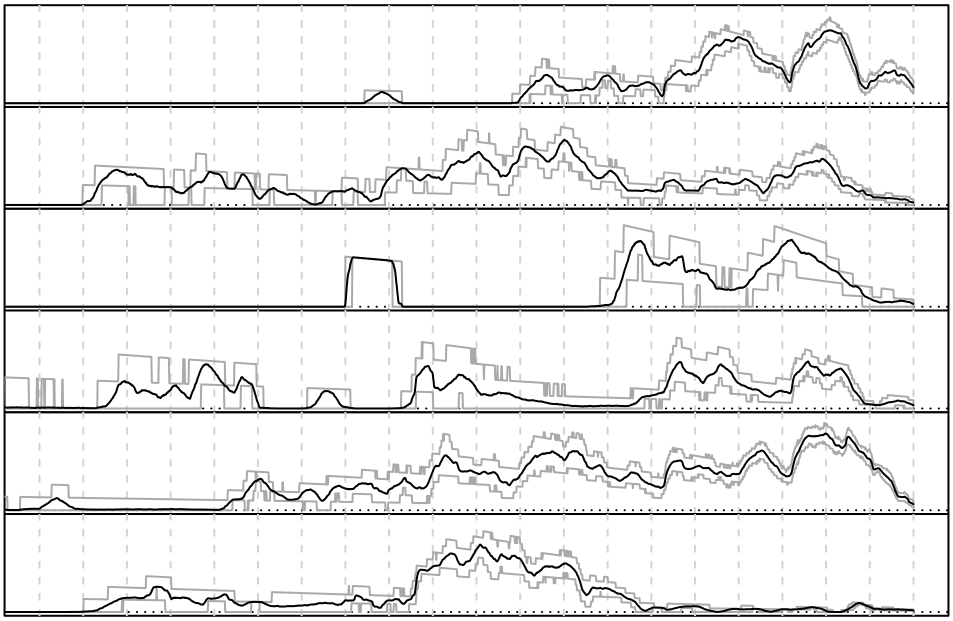

```
AlaskaCRFPABlist = list(
  ChukchiArcticCoastCRFPAB,
  BrooksPolarInlandCRFPAB,
  BeringInlandCRFPAB,
  BeringCoastCRFPAB,
  InteriorSouthernInlandCRFPAB,
  AleutiansCRFPAB,
  GulfOfAlaskaCRFPAB,
  KodiakCRFPAB
  )
names(AlaskaCRFPABlist) = c(
  "Arctic Coast",
  "Brooks Arctic Inland",
  "Bering Inland",
  "Bering Coast",
  "Forested Interior",
  "Aleutians",
  "Gulf of Alaska",
  "Kodiak"
  )

tfdStackPlot(tfdList = AlaskaCRFPABlist, xlim = c(10000, 0), h = h., text=FALSE)
```

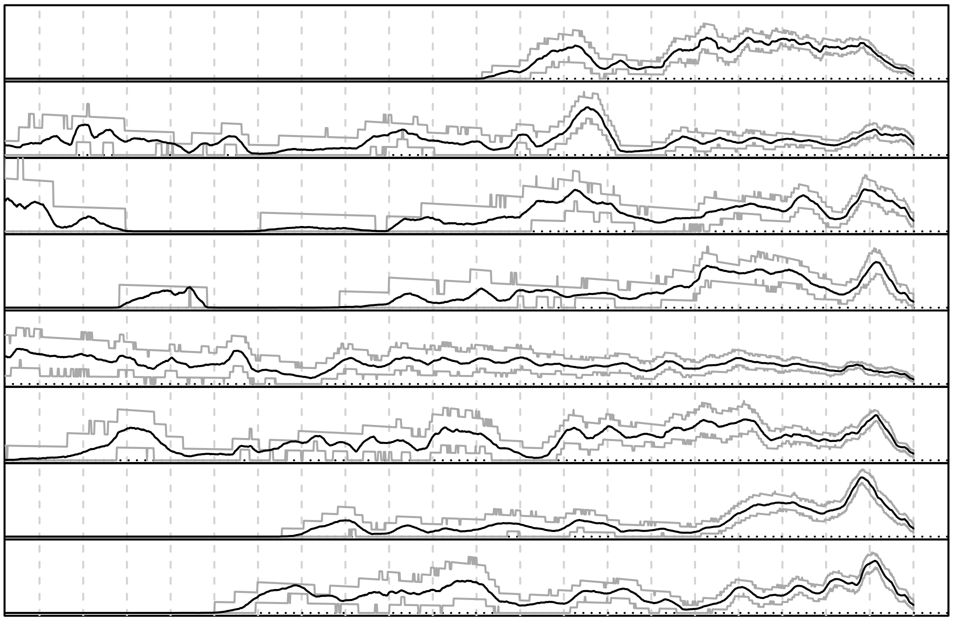

The following chunk writes out matrices as csv files, comprising simulated uncorrected and taphonomically corrected RFPABS, one per region. These are also used for the Moran's I analysis, below.

```
TFDs.for.csv = data.frame(

  # Alaska series
  ChukchiArcticCoast = rowMeans(ChukchiArcticCoastCRFPAB[[1]]),
  BeringCoast = rowMeans(BeringCoastCRFPAB[[1]]),
  GulfOfAlaska = rowMeans(GulfOfAlaskaCRFPAB[[1]]),
  SEAKCoast = rowMeans(SEAKCoastCRFPAB[[1]]),
  BeringInland = rowMeans(BeringInlandCRFPAB[[1]]),
  BrooksPolarInland = rowMeans(BrooksPolarInlandCRFPAB[[1]]),
  InteriorSouthernInland = rowMeans(InteriorSouthernInlandCRFPAB[[1]]),
  Kodiak = rowMeans(KodiakCRFPAB[[1]]),
  Aleutians = rowMeans(AleutiansCRFPAB[[1]]),

  # W. Hokkaido-Kurils series
  CentralHokkaidoInland = rowMeans(CentralHokkaidoInlandCRFPAB[[1]]),
  HokkaidoPacificCoast = rowMeans(HokkaidoPacificCoastCRFPAB[[1]]),
  HokkaidoSeaOfJapanCoast = rowMeans(HokkaidoSeaOfJapanCoastCRFPAB[[1]]),
  NorthernHokkaidoInland = rowMeans(NorthernHokkaidoInlandCRFPAB[[1]]),
  TsugaruStrait = rowMeans(TsugaruStraitCRFPAB[[1]]),
  Kuril = rowMeans(KurilCRFPAB[[1]])
  )
for(j in 1:ncol(TFDs.for.csv)){
  TFDs.for.csv[,j] = TFDs.for.csv[,j]/sum(TFDs.for.csv[,j])
```

```
  }
rownames(TFDs.for.csv) = timeline

correctedTFDs.for.csv = TFDs.for.csv * correctionFactor
for(j in 1:ncol(correctedTFDs.for.csv)){
  correctedTFDs.for.csv[,j] = correctedTFDs.for.csv[,j]/sum(correctedTFDs.for.csv[,j])
  }

#MAGRs.for.csv = apply(
#  X = log(TFDs.for.csv), MARGIN = 2, FUN = diff
#  )/5
#correctedMAGRs.for.csv = apply(
#  X = log(correctedTFDs.for.csv), MARGIN = 2, FUN = diff
#  )/5

write.csv(x = TFDs.for.csv, file = "NPacificTFDs.csv")
write.csv(x = correctedTFDs.for.csv, file = "correctedNPacificTFDs.csv")
#write.csv(x = MAGRs.for.csv, file = "NPacificMAGRs.csv")
#write.csv(x = correctedMAGRs.for.csv, file = "correctedNPacificMAGRs.csv")
```

4. Formal and computational details of the time iterative Moran's I (TIMI) analysis

4.1. Calculating Moran's I time series for MAGR when MAGR is unavailable for some regions at certain points in time

We wish to calculate a times series of Moran's I for a set of time series of mean adjusted growth rates (MAGR, or r), each based on a region-specific TFD. The MAGR is

$$r_{it} = \frac{\Delta \ln tfd_{it}}{\Delta t}$$

Here, t serves as an index for a step in the time series out of T steps, *not* a calendar timestamp. i is an index for the region for which the TFD has been assessed, out of N regions. Algebraically speaking, the MAGR is the average slope of the log-TFD function over an interval $[t, t+l)$, where l is a non-negative integer representing the lag from the beginning to the end of the interval over which the MAGR is evaluated. the change in log-TFD in the numerator is

$$\Delta \ln tfd_{it} = \ln tfd_{i,t+l} - \ln tfd_{it} = \ln \frac{tfd_{i,t+l}}{tfd_{it}}$$

Assuming 5-year intervals between steps in the time series, the denominator's size is

$$\Delta t = 5l$$

The time series of Moran's I_t is calculated as

$$I_t = \frac{\left(\frac{\sum_{i=1}^{N} \sum_{j=1}^{N} q_{ijt} w_{ij} d_{it} d_{jt}}{\sum_{i=1}^{N} \sum_{j=1}^{N} q_{ijt} w_{ij}} \right)}{\left(\frac{\sum_{i=1}^{N} q_{it} d_{it}^2}{\sum_{i=1}^{N} q_{it}} \right)} = \frac{\sum_{i=1}^{N} q_{it}}{\sum_{i=1}^{N} \sum_{j=1}^{N} q_{ijt} w_{ij}} \frac{\sum_{i=1}^{N} \sum_{j=1}^{N} q_{ijt} w_{ij} d_{it} d_{jt}}{\sum_{i=1}^{N} q_{it} d_{it}^2}$$

- q_{it} is an indicator variable equaling 1 if the MAGR for region i can be assessed at time t or 0 if not. This variable is not a standard feature of treatments of Moran's I but is necessary for a time series extension of this statistic, at least in cases where the contribution of different units to Moran's I_t turn on/off over time (e.g. if the MAGR cannot be calculated for some regions at certain points in time).
- q_{ijt} abbreviates the product $q_{it} q_{jt}$, yielding an indicator that equals 1 if and only if the MAGR for both region i and j can be assessed at time t, 0 otherwise.
- w_{ij} is a weight function and specifically an indicator variable, with a value either of 1 or 0. Typically, this is used as an adjacency indicator for dyad ij, but it may also/instead be used to indicate shared membership in some meaningful group (macro-region, ecological similarity), or even as a contrast indicator (contrasting macro-regions). w_{ij} is an element in a square ($N \times N$), symmetrical ($w_{ij} = w_{ji}$) matrix whose main diagonal ($i = j$) is $w_{ii} = 0$.
- d_{it} is the deviation of region i's MAGR from the average at time step t:

$$d_{it} = r_{it} - \bar{r}_t = r_{it} - \frac{\sum_{j=1}^{N} q_{jt} r_{jt}}{\sum_{j=1}^{N} q_{jt}}$$

In most cases, Moran's I_t will range between -1 and 1, but these are not strict boundaries. The statistic may transgress either of these boundaries particularly if the distribution of deviations $\{d_{1t}, \ldots, d_{Nt}\}$ at time

t is markedly skewed. Consequently, these boundaries can be strictly enforced if the weights w_{ijt} are row-standardized for time step t, rescaling all w_{ij} in row i by the reciprocal of the sum of "active-region" weights at time t:

$$w^*_{ijt} = \frac{q_{ijt} w_{ij}}{\sum_{h=1}^{N} q_{iht} w_{ih}}$$

In this case, the row sums of row-standardized weights will either be 1 (if $q_{ijt} = 1$ for any j) or 0 (if $q_{ijt} = 0$ for every j)—

$$\sum_{j=1}^{N} w^*_{ijt} = \begin{cases} 1 & \bigcup_{j=1}^{N} q_{ijt} = 1 \\ 0 & \bigcap_{j=1}^{N} q_{ijt} = 0 \end{cases}$$

—and therefore the sum of the row-standardized weight matrix (which serves as the denominator of the weighted average of off-diagonal products of deviations in the numerator of Moran's I_t) is the effective sample size at that time:

$$\sum_{i=1}^{N} q_{it} = \sum_{i=1}^{N} \sum_{j=1}^{N} w^*_{ij}$$

This further entails a simplification of I_t:

$$I_t = \frac{\left(\frac{\sum_{i=1}^{N} \sum_{j=1}^{N} q_{ijt} w_{ij} d_{it} d_{jt}}{\sum_{i=1}^{N} \sum_{j=1}^{N} q_{ijt} w_{ij}} \right)}{\left(\frac{\sum_{i=1}^{N} q_{it} d_{it}^2}{\sum_{i=1}^{N} q_{it}} \right)} = \frac{\left(\frac{\sum_{i=1}^{N} \sum_{j=1}^{N} q_{ijt} w_{ij} d_{it} d_{jt}}{\sum_{i=1}^{N} q_{it}} \right)}{\left(\frac{\sum_{i=1}^{N} q_{it} d_{it}^2}{\sum_{i=1}^{N} q_{it}} \right)}$$

$$= \frac{\sum_{i=1}^{N} q_{it}}{\sum_{i=1}^{N} q_{it}} \frac{\sum_{i=1}^{N} \sum_{j=1}^{N} q_{ijt} w_{ij} d_{it} d_{jt}}{\sum_{i=1}^{N} q_{it} d_{it}^2} = \frac{\sum_{i=1}^{N} \sum_{j=1}^{N} q_{ijt} w_{ij} d_{it} d_{jt}}{\sum_{i=1}^{N} q_{it} d_{it}^2}$$

The expected value for Moran's I_t is

$$\mathbb{E}[I_t] = -\frac{1}{\left(\sum_{i=1}^{N} q_{it} \right) - 1}$$

Assuming that the effective sample size at time t—

$$N_{eff,t} = \sum_{i=1}^{N} q_{it}$$

—is greater than or equal to 2, the expected value will be negative, approaching an upper asymptote of 0 as $N_{eff,t} \to \infty$.

4.2. Permutation tests

To evaluate whether an observed value of Moran's I_t is significantly different from expectation, we can implement a permutation test, in which

- The regional labels of the time series are switched or reshuffled at random without replacement, so that ever time series is reassigned to one out of N possible labels with equal probability;
- Moran's I_t is calculated for this random permutation and stored;
- A global test statistic quantifying the cumulative deviation of observed as well as each permuted Moran's I_t away from expectation over time is also calculated and stored. In the present study, the global statistic is calculated as the sum of squared differences between observed and expected Moran's I_t,

$$X^2 = \sum_{t=1}^{T} (I_t - \mathbb{E}[I_t])^2$$

- These three steps are repeated a large number of times, S, producing a vector of stored Moran's I_t as well as of X^2.
- The observed value of Moran's I_t is compared to the boundaries of the $100(1-\alpha)$ central interval of stored Moran's I_t for all permutations. This interval serves as a failure-to-reject interval for the null hypothesis: only if the observed value of Moran's I_t falls outside of this interval do we conclude that the observed data are significantly different from what we would expect under the null hypothesis of no autocorrelation.
- Likewise, the observed X^2 is compared to the boundaries of the $100(1-\alpha)$ central interval of X^2 calculated for all permuted time series. In this case, if the observed value falls outside of the failure-to-reject interval, then at least some parts of the observed time series of Moran's I_t are significantly different from the null hypothesis of no autocorrelation.

While permutation tests based on every possible permutation are in theory possible, they are usually impractical because there are $N! = \prod_{i=1}^{N} i$ permutations to evaluate, becoming computationally prohibitive even for modest values of N; the factorials of $N = 2$ through $N = 12$ are 2, 6, 24, 120, 720, 5040, 4.032×10^4, 3.6288×10^5, 3.6288×10^6, 3.99168×10^7, 4.790016×10^8, which poses an explosive computational challenge. Instead, S should be set to a reasonably large value (e.g., 1000, 10000, or similar). This simulation-based approach to permutation tests maintains reasonable computing time and memory while also insuring that the simulated approximation of the sampling distribution of the focal statistic between permutations is reasonably stable.

4.3. Computation

- The data are loaded as fourteen regional TFD time series, assessed at 5-calendar-year intervals. This includes two data sets: taphonomy-corrected and uncorrected TFDs.

```
TFD.uncorrected.dat = TFDs.for.csv
TFD.uncorrected.dat = data.frame(
  calBP = timeline,
  TFD.uncorrected.dat
)
#TFD.uncorrected.dat =  read.csv(
#  file = "NPacificTFDs.csv",
#  stringsAsFactors = FALSE
```

```
#  )

TFD.corrected.dat = correctedTFDs.for.csv
TFD.corrected.dat = data.frame(
  calBP = timeline,
  TFD.corrected.dat
  )
#TFD.corrected.dat = read.csv(
#  file = "correctedNPacificTFDs.csv",
#  stringsAsFactors = FALSE
#  )
```

- Four distinct dyadic weight matrices are constructed. As noted above, these are square, symmetrical $N \times N$ matrices such that $w_{ij} = w_{ji}$ and the main diagonal $w_{ii} = 0$. The first weight matrix—the **shared-region** weight matrix—assigns unit weights (=1) to pairs of regions belonging to the same macro-region (NW Pacific Rim, i.e. Hokkaido-Kurils, versus NE Pacific Rim, i.e. Alaska) and assigns null weights (=0) to pairs of regions belonging to opposite macro-regions. The second weight matrix—the **between-regions** weight matrix—assigns unit weights to pairs of regions belonging to opposite macro-regions and null weights to pairs of regions belonging to the same macro-region. The third weight matrix—the **eco-similarity** weight matrix—assigns unit weights to pairs of regions that are ecologically similar (either both coastal or both inland) and null weights to pairs of regions that are ecologically dissimilar. The fourth weight matrix—the **adjacency** weight matrix—assigns unit weights to pairs of regions that are spatially adjacent and null weights to pairs of regions that are spatially nonadjacent. (Note: there are 19 realized adjacencies out of 14(13)/2=91 possible adjacencies for the full set of 14 regions.)

```
#colnames(TFD.uncorrected.dat)

# AK vs. NW Pacific regions
region.AK = c(
  "ChukchiArcticCoast", "BeringCoast",
  "GulfOfAlaska", "SEAKCoast",
  "Kodiak", "Aleutians",
  "BeringInland", "BrooksPolarInland", "InteriorSouthernInland"
  )
region.NWPac = c(
  "HokkaidoPacificCoast", "HokkaidoSeaOfJapanCoast",
  "TsugaruStrait", "Kuril",
  "CentralHokkaidoInland", "NorthernHokkaidoInland"
  )

# Coastal vs. inland regions, Bering inland is inland
ecosimilarity.Coast1 = c(
  "ChukchiArcticCoast", "BeringCoast",
  "GulfOfAlaska", "SEAKCoast",
  "Kodiak", "Aleutians",
  "HokkaidoPacificCoast", "HokkaidoSeaOfJapanCoast",
  "TsugaruStrait", "Kuril"
  )
ecosimilarity.Inland1 = c(
  "BeringInland", "BrooksPolarInland", "InteriorSouthernInland",
  "CentralHokkaidoInland", "NorthernHokkaidoInland"
  )
```

```r
# Coastal vs. inland regions, Bering inland is coastal
ecosimilarity.Coast2 = c(
  "ChukchiArcticCoast", "BeringCoast", "BeringInland",
  "GulfOfAlaska", "SEAKCoast",
  "Kodiak", "Aleutians",
  "HokkaidoPacificCoast", "HokkaidoSeaOfJapanCoast",
  "TsugaruStrait", "Kuril"
  )
ecosimilarity.Inland2 = c(
  "BrooksPolarInland", "InteriorSouthernInland",
  "CentralHokkaidoInland", "NorthernHokkaidoInland"
  )

# Adjacency list
adjacency.list = rbind(
  #AK regions
  c("SEAKCoast", "GulfOfAlaska"),
  c("ChukchiArcticCoast", "BeringCoast"),
  c("ChukchiArcticCoast", "BrooksPolarInland"), #
  c("BeringCoast", "BeringInland"),
  c("BeringCoast", "Aleutians"),
  c("BeringCoast", "GulfOfAlaska"),
  c("BrooksPolarInland", "InteriorSouthernInland"),
  c("BrooksPolarInland", "BeringInland"),
  c("BeringInland", "InteriorSouthernInland"),
  c("BeringInland", "GulfOfAlaska"),
  c("InteriorSouthernInland", "GulfOfAlaska"),
  c("GulfOfAlaska", "Kodiak"),

  # NW Pacific regions
  c("Kuril", "HokkaidoPacificCoast"),
  c("HokkaidoPacificCoast", "HokkaidoSeaOfJapanCoast"),
  c("HokkaidoPacificCoast", "CentralHokkaidoInland"),
  c("HokkaidoPacificCoast", "TsugaruStrait"),
  c("HokkaidoSeaOfJapanCoast", "NorthernHokkaidoInland"),
  c("HokkaidoSeaOfJapanCoast", "CentralHokkaidoInland"),
  c("HokkaidoSeaOfJapanCoast", "TsugaruStrait"),
  c("NorthernHokkaidoInland", "CentralHokkaidoInland"),
  c("CentralHokkaidoInland", "TsugaruStrait")
  )

regionMat = crossRegionMat = ecosimilarityMat1 = ecosimilarityMat2 = adjacencyMat = matrix(
  data = 0,
  nrow = ncol(TFD.uncorrected.dat)-1,
  ncol = ncol(TFD.uncorrected.dat)-1,
  dimnames = list(
    colnames(TFD.uncorrected.dat)[2:ncol(TFD.uncorrected.dat)],
    colnames(TFD.uncorrected.dat)[2:ncol(TFD.uncorrected.dat)]
    )
  )
for(i in 2:nrow(regionMat)){for(j in 1:i){
  # Within-region pairs
  if(
```

```r
    ((
      length(intersect(x = rownames(regionMat)[i], region.AK))>0 &
      length(intersect(x = rownames(regionMat)[j], region.AK))>0
      ) | (
      length(intersect(x = rownames(regionMat)[i], region.NWPac))>0 &
      length(intersect(x = rownames(regionMat)[j], region.NWPac))>0
      )) &
    rownames(regionMat)[i] != rownames(regionMat)[j]
    ){regionMat[i,j]=regionMat[j,i]=1}

  # Between-region pairs
  if(

    (
    length(intersect(x = rownames(crossRegionMat)[i], region.AK))>0 &
    length(intersect(x = rownames(crossRegionMat)[j], region.NWPac))>0
    ) |

    (
    length(intersect(x = rownames(crossRegionMat)[i], region.NWPac))>0 &
    length(intersect(x = rownames(crossRegionMat)[j], region.AK))>0
    )

    ){crossRegionMat[i,j]=crossRegionMat[j,i]=1}

  # Coastal vs. inland regions, Bering inland is inland
  if(
    ((
      length(intersect(x = rownames(ecosimilarityMat1)[i], ecosimilarity.Coast1))>0 &
      length(intersect(x = rownames(ecosimilarityMat1)[j], ecosimilarity.Coast1))>0
      ) | (
      length(intersect(
        x = rownames(ecosimilarityMat1)[i], ecosimilarity.Inland1
        ))>0 &
      length(intersect(
        x = rownames(ecosimilarityMat1)[j], ecosimilarity.Inland1
        ))>0
      )) &
    rownames(ecosimilarityMat1)[i] != rownames(ecosimilarityMat1)[j]
    ){ecosimilarityMat1[i,j]=ecosimilarityMat1[j,i]=1}

    # Coastal vs. inland regions, Bering inland is coastal
  if(
    ((
      length(intersect(x = rownames(ecosimilarityMat2)[i], ecosimilarity.Coast2))>0 &
      length(intersect(x = rownames(ecosimilarityMat2)[j], ecosimilarity.Coast2))>0
      ) | (
      length(intersect(
        x = rownames(ecosimilarityMat2)[i], ecosimilarity.Inland2
        ))>0 &
      length(intersect(
        x = rownames(ecosimilarityMat2)[j], ecosimilarity.Inland2
        ))>0
```

```
    )) &
    rownames(ecosimilarityMat2)[i] != rownames(ecosimilarityMat2)[j]
    ){ecosimilarityMat2[i,j]=ecosimilarityMat2[j,i]=1}

  # Adjacent regions
  for(dyad in 1:nrow(adjacency.list)){
    if(
      length(intersect(
        rownames(adjacencyMat)[i], adjacency.list[dyad,]
        ))>0 &
      length(intersect(
        rownames(adjacencyMat)[j], adjacency.list[dyad,]
        ))>0
      & rownames(adjacencyMat)[i] != rownames(adjacencyMat)[j]
      ){adjacencyMat[i,j] = adjacencyMat[j,i] = 1}
    }
  }}
#View(regionMat)
#View(crossRegionMat)
#View(ecosimilarityMat)
#sum(adjacencyMat)/2; View(adjacencyMat)
```

- A flexible function is programmed that allows several inputs:
  - A matrix with T rows and N columns, for N TFD time series of length T. This can take either taphonomy-corrected or uncorrected TFD time series. If no explicit input is provided, the default is the uncorrected data set.
  - A dyadic weight matrix. If no explicit input is provided, the default is the shared-region matrix.
  - A temporal granularity (Δt) over which the MAGR time series should be calculated. This should be a multiple of 5, where the value 5 will result in a non-overlapping series of MAGR evaluations and anything greater will result in overlapping evaluations. If no explicit input is provided, the default is 200. This value is favored because our TFDs are constructed through composite redundancy filtering through presence-absence buffering (CRFPAB), where the each buffer has a width of $2h$ and $h = 100$.
  - A list of regions to exclude globally from the analysis. If no explicit input is provided, the default excludes the Tsugaru Strait and the Southeast Alaskan Coast TFD time series.
  - A size for the permutation test. If no explicit input is provided, the default is $S = 1000$.
  - A seed number for the random number generator used in the permutation simulation. If no explicit input is provided, the default is 2021.
  - A level of significance α for permutation tests. If no explicit input is provided, the default is $\alpha = 0.01$.
  - A temporal upper boundary (a multiple of 5 in cal BP) specifying the interval over which the global test statistic X^2 should be calculate. If not explicit input is provided, the default is 5000 cal BP.
- Output of this function includes
  - A cal BP timeline for plotting output of the function, truncated at the recent end to accommodate the fact that growth rates cannot be calculated for end-series time steps, where the lag $t + l > T$.
  - A time series vector of Moran's I_t based on the observed data set.
  - A matrix of time series of Moran's I_t based on permuted data sets.
  - Boundaries of the fail-to-reject region for each observed I_t in the time series, assuming a pre-specified α.
  - X^2 for the observed time series, which allows for a global test of autocorrelation across the time series.

- A vector of X^2, one element per permuted time series, which allows for a global test of autocorrelation across the observed time series.
- The upper boundary of the fail-to-reject region (lower boundary of the rejection region) for a right-tailed test using this global test statistic, assuming a pre-specified α.
- A vector of MAGR time series for all regions included in the analysis.
- For each region, a list of one or more intervals of time (in cal BP) during which that region contributes to the analysis.
- A $T \times N$ matrix for q_{it}, from which $N_{eff,t}$ can be calculated as the row sum $\sum_{i=1}^{N} q_{it}$.

```
MoransIanalysis=function(
  TFDs=TFD.uncorrected.dat,
  weightMatrix=regionMat,
  temporalGranularity=200, # Must be a multiple of 5
  regionsToExclude = c("TsugaruStrait", "SEAKCoast"),
  S = 1000,
  seed = 2022,
  alpha = 0.01,
  globalStatStart = 5000
){
  # Breaking the TFD object into the TFD and the timeline
  calBP = TFDs$calBP; TFDs = TFDs[,-1]

  # Excluding pre-specified sites from analysis
  exclusionIndexes = rep(NA, length(regionsToExclude))
  for(i in 1:length(exclusionIndexes)){
    exclusionIndexes[i]=which(
      rownames(weightMatrix)==regionsToExclude[i]
      )
    }
  exclusionIndexes = -unique(exclusionIndexes)
  weightMatrixExclusive = weightMatrix[
    exclusionIndexes,exclusionIndexes
    ]
  TFDsExclusive = TFDs[,exclusionIndexes]
  N.effective = ncol(TFDsExclusive)

  # Calculating the MAGR time series
  TG.factor = temporalGranularity/5
  MAGR.TS = apply(
    X = log(TFDsExclusive), MARGIN = 2, FUN = diff, lag = TG.factor
    ) / temporalGranularity
  T.effective = nrow(MAGR.TS)
  rownames(MAGR.TS) = calBP[1:T.effective]
  MAGR.TS[is.na(MAGR.TS)] = NA; MAGR.TS[is.infinite(MAGR.TS)] = NA
  MAGRdev.TS = MAGR.TS - rowMeans(MAGR.TS, na.rm = TRUE)

  # Assessing which and how many regions switch on/off
  onOffMat = MAGRdev.TS; onOffMat[,] = NA
  onOffMat[1,] = ifelse(
    test=!is.na(MAGRdev.TS[1,]),
    yes = 1, no = NA
    )
  for(i in 2:(T.effective-1)){
    onOffMat[i,] = ifelse(
```

```
    test =
      is.na(MAGRdev.TS[i-1,]) &
      !is.na(MAGRdev.TS[i,]) &
      !is.na(MAGRdev.TS[i+1,]),
    yes = 1, no = NA
    )
  onOffMat[i,] = ifelse(
    test =
      is.na(MAGRdev.TS[i-1,]) &
      !is.na(MAGRdev.TS[i,]) &
      is.na(MAGRdev.TS[i+1,]),
    yes = 0, no = onOffMat[i,]
    )
  onOffMat[i,] = ifelse(
    test =
      !is.na(MAGRdev.TS[i-1,]) &
      !is.na(MAGRdev.TS[i,]) &
      is.na(MAGRdev.TS[i+1,]),
    yes = -1, no = onOffMat[i,]
    )
  }
onOffMat[T.effective,] = ifelse(
  test = !is.na(MAGRdev.TS[T.effective,]),
  yes = -1, no = NA
  )
onOffList = list()
for(j in 1:N.effective){
  onOffList[[j]] = data.frame(
    on=calBP[as.numeric(which(onOffMat[,j]==1 | onOffMat[,j]==0))],
    off=calBP[as.numeric(which(onOffMat[,j]==0 | onOffMat[,j]==-1))]
    )
  names(onOffList)[j] = colnames(onOffMat)[j]
  }
whichOn = ifelse(!is.na(MAGRdev.TS),1,0)
N_eff.t = rowSums(whichOn)
expectedI_t = -1/(N_eff.t - 1)

# Calculating the original and permuted Moran's I
outerProdArray = array(
  data = NA,
  dim = c(N.effective, N.effective, T.effective)
  )
for(t in 1:T.effective) outerProdArray[,,t]=outer(
  MAGRdev.TS[t,], MAGRdev.TS[t,]
  )

permutedMoransI = matrix(
  data = NA,
  nrow = T.effective, ncol=S+1,
  dimnames = list(
    rownames(MAGRdev.TS),
    paste0("s=", 0:S)
    )
```

```
  )
sumSqDev = rowSums(MAGRdev.TS^2, na.rm = TRUE)

permutationIndexes = matrix(
  NA,
  nrow = S+1, ncol = N.effective
  )
permutationIndexes[1,] = observedIndexes = 1:N.effective
set.seed(seed)
for(s in 2:(S+1)){
  permutationIndexes[s,] = sample(
    x = observedIndexes, size = N.effective, replace = FALSE
    )
  }

for(s in 1:(S+1)){ # This is the big time suck.
  sortedOuterProdArray = outerProdArray[
    permutationIndexes[s,], permutationIndexes[s,],
    ]
  for(t in 1:T.effective){
    dropInds = as.numeric(
      which(is.na(MAGRdev.TS[t,permutationIndexes[s,]]))
      )
    if(length(dropInds)>0){
      weightMatrix.t = weightMatrixExclusive[-dropInds,-dropInds]
      sortedOuterProdMatrix=sortedOuterProdArray[
        -dropInds,-dropInds,t
        ]
      }else{
        weightMatrix.t = weightMatrixExclusive
        sortedOuterProdMatrix=sortedOuterProdArray[,,t]
        }
    if(length(weightMatrix.t)==1){
      weightMatrix.t=matrix(weightMatrixExclusive, nrow=1, ncol=1)
      }
    stdWeightMat = weightMatrix.t/rowSums(weightMatrix.t)
    permutedMoransI[t,s] =
      sum(
        sortedOuterProdMatrix*stdWeightMat
        ) /
      sumSqDev[t]
    }
  }
startInd=which(as.numeric(rownames(permutedMoransI))==globalStatStart)
permutedXsq = colSums(
  x = (
    permutedMoransI[startInd:T.effective,] -
    expectedI_t[startInd:T.effective]
    )^2,
  na.rm = TRUE
  )

# Outputting objects resulting from the analysis
```

```
  return(list(
    calBP = as.numeric(rownames(permutedMoransI)),
    observedMoransI.TS = permutedMoransI[,1],
    permutedMoransI.TS = permutedMoransI[,-1],
    failToRejectBoundaries = t(apply(
      X = permutedMoransI,
      MARGIN = 1,
      FUN = quantile,
      probs = c(0.5-0.5*(1-alpha), 0.5+0.5*(1-alpha)),
      na.rm = TRUE
      )),
    observedXsq = permutedXsq[1],
    permutedXsq = permutedXsq[-1],
    failToRejectBoundariesGlobal = quantile(
      x = permutedXsq[-1],
      probs = (1-alpha)
      ),
    MAGR.TS = MAGR.TS,
    onOffList = onOffList,
    whichOn = whichOn,
    N_eff.t = N_eff.t,
    expectedI_t = expectedI_t
    ))
  }
```

4.4. Results

```
#S = 25
S = 5000
alpha = 0.05
```

The following analyses are based on the taphonomy-corrected TFDs. S is set to 5000 and α is set to 0.05. Each permutation takes approximately 1/10 of a second to complete assuming 12 regions, or quicker for smaller analyses. The first analysis (`Eval01`) uses the shared-region weight matrix. The second (`Eval02`) uses the between-regions weight matrix. The third (`Eval03`) uses the eco-similarity matrix. The fourth (`Eval04`) uses the adjacency weight matrix. A fifth and sixth analysis (`Eval05` and `Eval06`) repeat the first analysis, using the shared-region weight matrix, but the fifth and sixth analyses stratify the analysis by considering coastal and inland regions separately All analyses exculde Tsugaru Strait and the SE AK Coast.

```
# Regional membership analysis
Eval01=MoransIanalysis(
  TFDs = TFD.corrected.dat,
  weightMatrix = regionMat,
  temporalGranularity = 200,
  S = S,
  alpha = alpha
  )

# Regional contrast (cross-regional pairs) analysis
Eval02=MoransIanalysis(
  TFDs = TFD.corrected.dat,
```

```r
  weightMatrix = crossRegionMat,
  temporalGranularity = 200,
  S = S,
  alpha = alpha
  )

# Ecosimilarity analysis, Bering inland is inland
Eval03.1=MoransIanalysis(
  TFDs = TFD.corrected.dat,
  weightMatrix = ecosimilarityMat1,
  temporalGranularity = 200,
  S = S,
  alpha = alpha
  )

# Ecosimilarity analysis, Bering inland is coastal
Eval03.2=MoransIanalysis(
  TFDs = TFD.corrected.dat,
  weightMatrix = ecosimilarityMat1,
  temporalGranularity = 200,
  S = S,
  alpha = alpha
  )

# Adjacency analysis
Eval04=MoransIanalysis(
  TFDs = TFD.corrected.dat,
  weightMatrix = adjacencyMat,
  temporalGranularity = 200,
  S = S,
  alpha = alpha
  )

# Regional membership analysis, coastal stratum, Bering inland is inland
Eval05.1=MoransIanalysis(
  TFDs = TFD.corrected.dat,
  weightMatrix = regionMat,
  temporalGranularity = 200,
  regionsToExclude = c(
    "TsugaruStrait", "SEAKCoast",
    ecosimilarity.Inland1
    ),
  S = S,
  alpha = alpha
  )

# Regional membership analysis, coastal stratum, Bering inland is coastal
Eval05.2=MoransIanalysis(
  TFDs = TFD.corrected.dat,
  weightMatrix = regionMat,
  temporalGranularity = 200,
  regionsToExclude = c(
    "TsugaruStrait", "SEAKCoast",
```

```r
    ecosimilarity.Inland2
    ),
  S = S,
  alpha = alpha
  )

# Regional membership analysis, inland stratum, Bering inland is inland
Eval06.1=MoransIanalysis(
  TFDs = TFD.corrected.dat,
  weightMatrix = regionMat,
  temporalGranularity = 200,
  regionsToExclude = c(
    "TsugaruStrait", "SEAKCoast",
    ecosimilarity.Coast1
    ),
  S = S,
  alpha = alpha
  )

# Regional membership analysis, inland stratum, Bering inland is coastal
Eval06.2=MoransIanalysis(
  TFDs = TFD.corrected.dat,
  weightMatrix = regionMat,
  temporalGranularity = 200,
  regionsToExclude = c(
    "TsugaruStrait", "SEAKCoast",
    ecosimilarity.Coast2
    ),
  S = S,
  alpha = alpha
  )
```

The following figure plots the effective sample size as this changes over time *for the full period covered by the calibration curve.*

```
Eval = Eval01

plot(
  x = Eval$calBP,
    xlim = rev(range(Eval$calBP)),
    xlab = "cal BP",
  y = Eval$N_eff.t,
    ylab = expression(N[eff]),
  type = "s"
  )
abline(v=5000, lty = 2, col = "red")
```

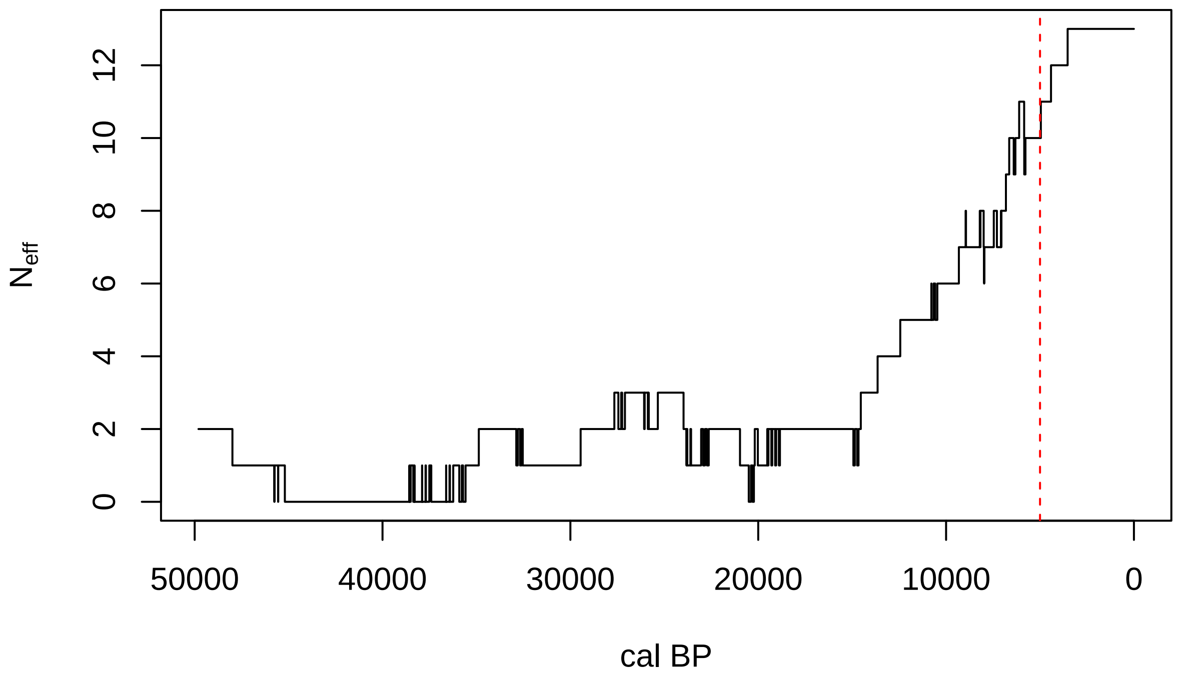

The following figure plots the intervals during which each region has finite MAGR values, color-coded by macroregion, with solid lines for coastal regions and dashed for inland. (Bering inland is treated as inland here.) This plot illustrates that, even if the effective sample size at any given point in time is >1, the ability to calculate Moran's I may still be constrained by the fact that the regions with finite MAGR at that time belong to the same macro-regional or eco-type groups. This constraint will be even more salient for stratified analyses.

```
plot(
  x=NA,
  xlim = c(50000, 0), ylim = c(1, length(Eval)),
  xlab = "cal BP",
  ylab = NA, yaxt="n",
  main = "Finite MAGR intervals"
  )
for(i in 1:length(Eval)){
  segments(
    y0=i,
    x0=Eval$onOffList[[i]][,1], x1 = Eval$onOffList[[i]][,2],
    col = ifelse(
      test=anyDuplicated(c(names(Eval$onOffList)[i], region.AK))>0,
      yes="blue", no = "red"
      ),
    lty = ifelse(
      test=anyDuplicated(c(
        names(Eval$onOffList)[i],
        ecosimilarity.Coast1
        ))>0,
      yes=1, no = 2
      )
    )
  }
abline(v = 5000, lty = 2)
legend(
  "bottomleft", bty="n",
  legend = c(
    "Alaska, coastal",
    "Alaska, inland",
    "Hokkaido-Kurils, coastal",
    "Hokkaido-Kurils, inland"
    ),
  lty = c(1,2,1,2), col = c("blue", "blue", "red", "red")
  )
```

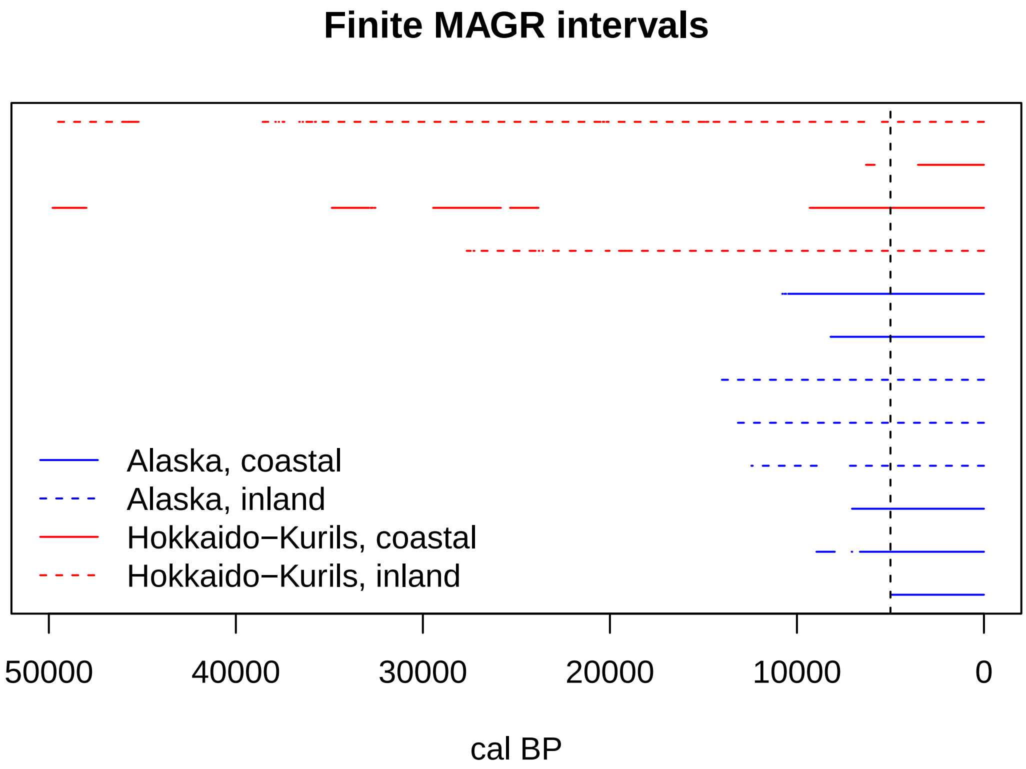
Finite MAGR intervals
Alaska, coastal
Alaska, inland
Hokkaido−Kurils, coastal
Hokkaido−Kurils, inland
50000
40000
30000
20000
10000
0
cal BP

The following figures plot the regional MAGR time series, color coded either for macroregional groups or for ecological type. (The second figure treats Bering Inland as inland.) At any point in time When MAGR time series of like color cluster with each other but separate from time series of contrasting color, we should expect high values of Moran's I.

```
# Color-coded for region
colMacroregion = rep(NA, ncol(Eval$MAGR.TS))
for(i in 1:length(colMacroregion)){
  colMacroregion[i] = ifelse(
      length(intersect(colnames(Eval$MAGR.TS)[i], region.AK))>0,
      "blue",
      "red"
      )
  }
plot(
  x = Eval$calBP,
  #xlim = rev(range(Eval$calBP)),
  xlim = c(5000, 0),
  xlab = "cal BP",

  y = Eval$MAGR.TS[,1],
  ylim = range(Eval$MAGR.TS, na.rm = TRUE),
  ylab =  expression(paste(bar(r))),

  type = "l", lty = 1,
  col = colMacroregion[1]
  )
for(j in 2:ncol(Eval$MAGR.TS)){
  lines(
    x = Eval$calBP,
    y = Eval$MAGR.TS[,j],
    lty = j,
    col = colMacroregion[j]
    )
  }
legend(
  "bottom", bty = "n",
  legend = colnames(Eval$MAGR.TS),
  lty = 1:ncol(Eval$MAGR.TS),
  col = colMacroregion,
  cex = .6, horiz = FALSE, ncol = 3
  )
```

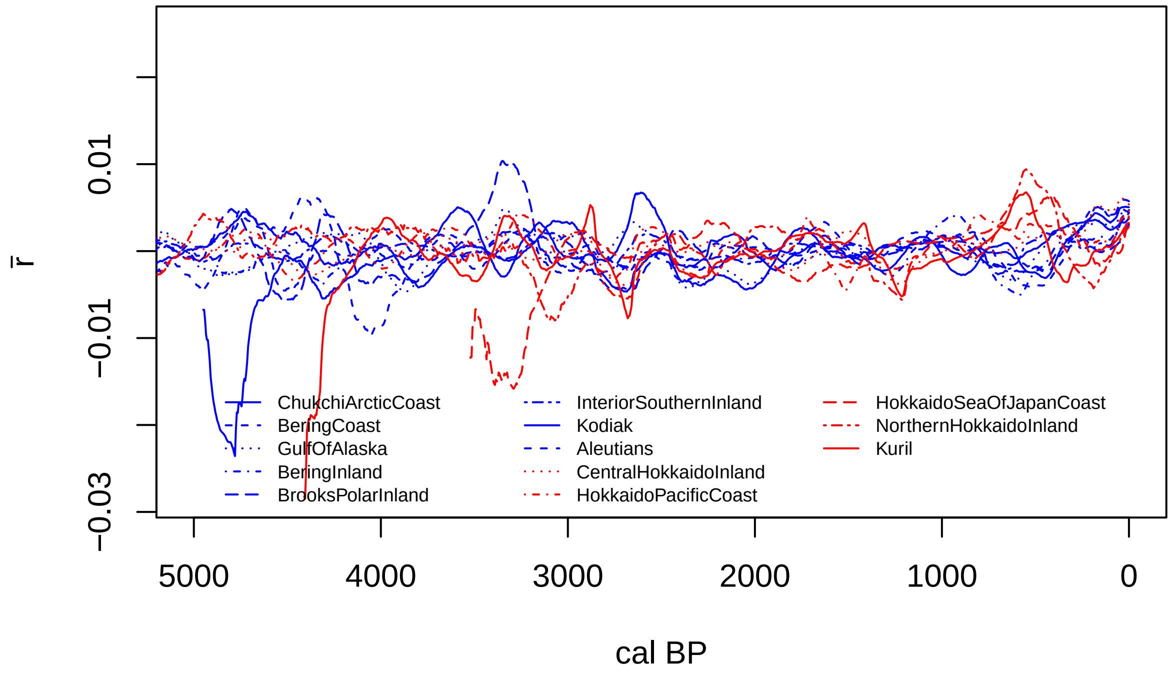


```
# Color-coded for eco-type
colEcosimilarity = rep(NA, ncol(Eval$MAGR.TS))
for(i in 1:length(colEcosimilarity)){
  colEcosimilarity[i] = ifelse(
      length(intersect(colnames(Eval$MAGR.TS)[i],ecosimilarity.Coast1))>0,
      "blue",
      "red"
      )
  }
plot(
  x = Eval$calBP,
  #xlim = rev(range(Eval$calBP)),
  xlim = c(5000, 0),
  xlab = "cal BP",

  y = Eval$MAGR.TS[,1],
  ylim = range(Eval$MAGR.TS, na.rm = TRUE),
  ylab =  expression(paste(bar(r))),

  type = "l", lty = 1,
  col = colEcosimilarity[1]
  )
for(j in 2:ncol(Eval$MAGR.TS)){
  lines(
    x = Eval$calBP,
    y = Eval$MAGR.TS[,j],
```

```
    lty = j,
    col = colEcosimilarity[j]
    )
  }
legend(
  "bottom", bty = "n",
  legend = colnames(Eval$MAGR.TS),
  lty = 1:ncol(Eval$MAGR.TS),
  col = colEcosimilarity,
  cex = .6, horiz = FALSE, ncol = 3
  )
```

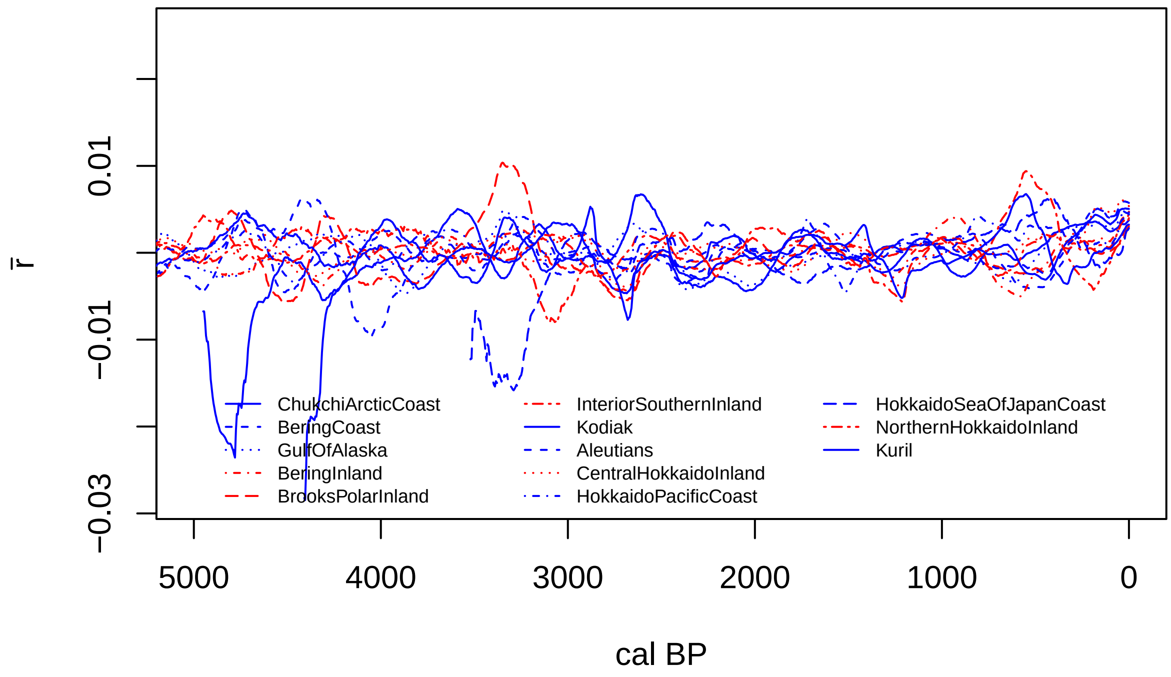

The I_t time series for the within-region analysis are presented below for the last 5000 years:

```
#plottingXlim = rev(range(Eval$calBP))
plottingXlim = c(5000, 0)
#plottingXlim = c(10000, 0)
Eval = Eval01

plot(
  x = Eval$calBP,
  xlim = plottingXlim,
  xlab = "cal BP",

  y = Eval$observedMoransI.TS,
  ylim = c(-1, 1),
  ylab = "I",
  type = "l",

  main = "Shared-region analysis, unstratified"
  )
abline(
  h = c(-1, 1), lty = 3
  )
lines(
  x = Eval$calBP,
  y = -1/(rowSums(Eval$whichOn)-1),
  col = "red", type = "s"
  )
lines(
  x = Eval$calBP,
  y = Eval$failToRejectBoundaries[,1],
  col = "red", lty = 2
  )
lines(
  x = Eval$calBP,
  y = Eval$failToRejectBoundaries[,2],
  col = "red", lty = 2
  )
legend(
  "topleft", bty = "n",
  legend = c(
    expression(I[t]),
    expression(paste("E[",I[t],"]")),
    "Fail-to-reject interval"
    ),
  lty = c(1,1,2), col = c("black", "red", "red")
  )
```

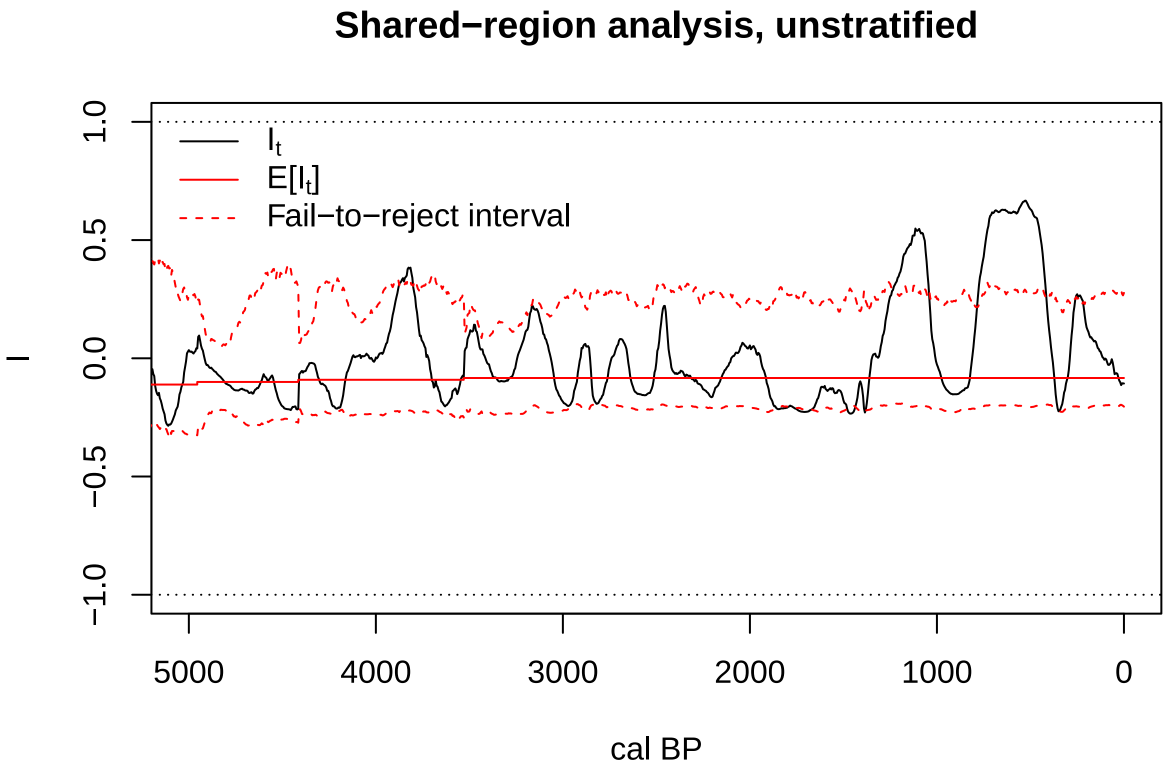
Shared−region analysis, unstratified
1.0
0.5
0.0
−0.5
−1.0
I
I_t
$E[I_t]$
Fail−to−reject interval
5000
4000
3000
2000
1000
0
cal BP

The I_t time series for the between-region analysis **after omitting inland regions** (Bering Inland counts as inland, `Eval05.1`) is presented below for the last 5000 years:

```
Eval = Eval05.1

plot(
  x = Eval$calBP,
  xlim = plottingXlim,
  xlab = "cal BP",

  y = Eval$observedMoransI.TS,
  ylim = c(-1, 1),
  ylab = "I",
  type = "l",

  main = "Shared-region analysis, coastal stratum"
  )
abline(
  h = c(-1, 1), lty = 3
  )
lines(
  x = Eval$calBP,
  y = -1/(rowSums(Eval$whichOn)-1),
  col = "red", type = "s"
  )
lines(
  x = Eval$calBP,
  y = Eval$failToRejectBoundaries[,1],
  col = "red", lty = 2
  )
lines(
  x = Eval$calBP,
  y = Eval$failToRejectBoundaries[,2],
  col = "red", lty = 2
  )
legend(
  "topleft", bty = "n",
  legend = c(
    expression(I[t]),
    expression(paste("E[",I[t],"]")),
    "Fail-to-reject interval"
    ),
  lty = c(1,1,2), col = c("black", "red", "red")
  )
```

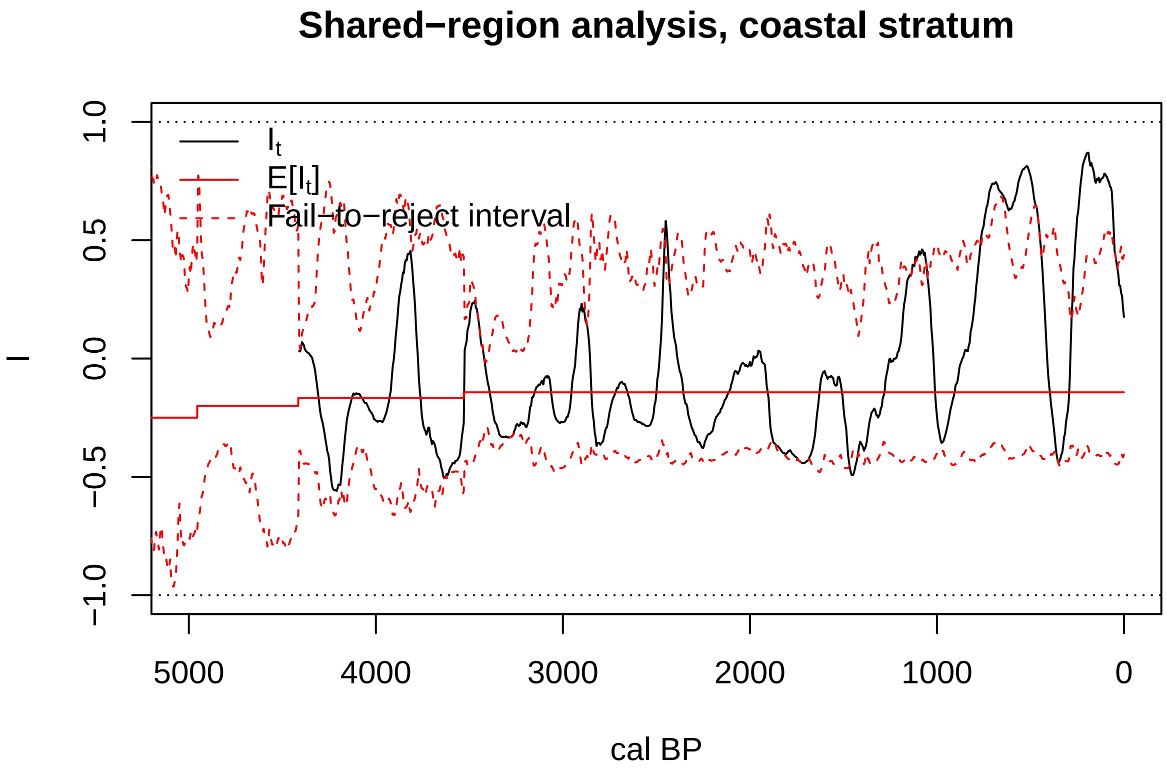

Shared−region analysis, coastal stratum
I
1.0
0.5
0.0
−0.5
−1.0
I_t
$E[I_t]$
Fail−to−reject interval
5000
4000
3000
2000
1000
0
cal BP

The I_t time series for the between-region analysis **after omitting inland regions** (Bering Inland counts as coastal, `Eval05.2`) is presented below for the last 5000 years:

```
Eval = Eval05.2

plot(
  x = Eval$calBP,
  xlim = plottingXlim,
  xlab = "cal BP",

  y = Eval$observedMoransI.TS,
  ylim = c(-1, 1),
  ylab = "I",
  type = "l",

  main = "Shared-region analysis, coastal stratum"
  )
abline(
  h = c(-1, 1), lty = 3
  )
lines(
  x = Eval$calBP,
  y = -1/(rowSums(Eval$whichOn)-1),
  col = "red", type = "s"
  )
lines(
  x = Eval$calBP,
  y = Eval$failToRejectBoundaries[,1],
  col = "red", lty = 2
  )
lines(
  x = Eval$calBP,
  y = Eval$failToRejectBoundaries[,2],
  col = "red", lty = 2
  )
legend(
  "topleft", bty = "n",
  legend = c(
    expression(I[t]),
    expression(paste("E[",I[t],"]")),
    "Fail-to-reject interval"
    ),
  lty = c(1,1,2), col = c("black", "red", "red")
  )
```

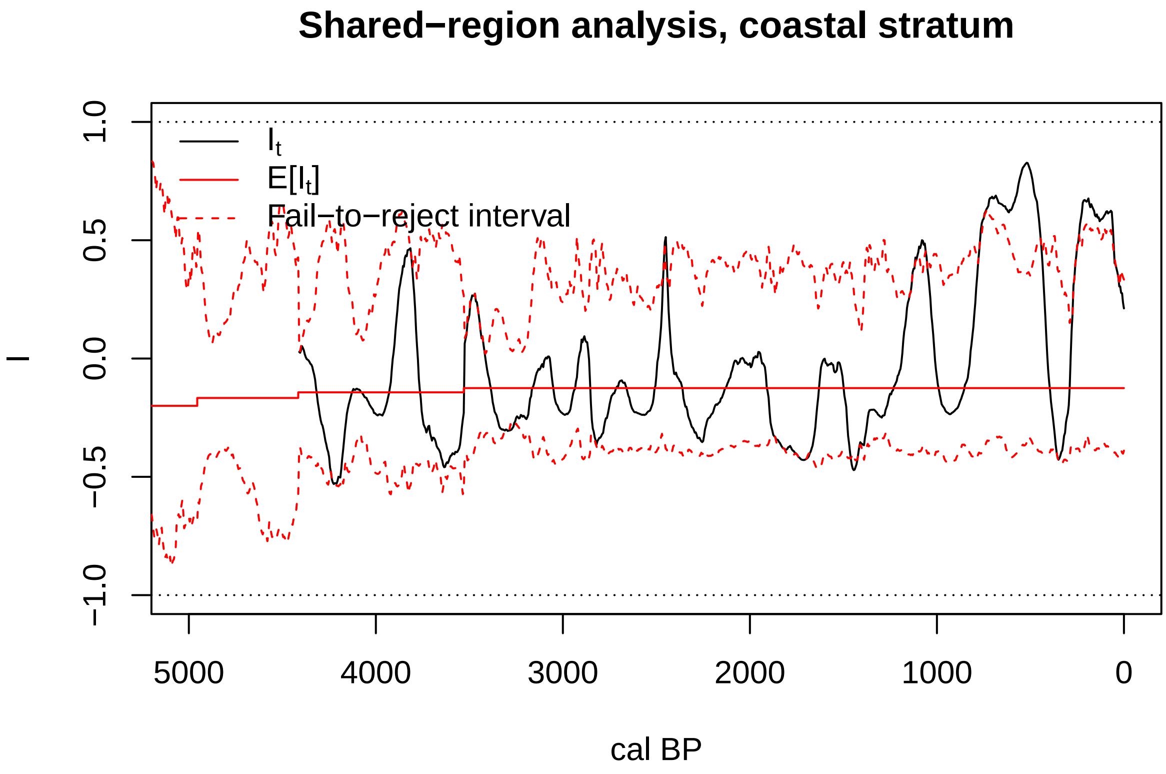

Shared−region analysis, coastal stratum
1.0
0.5
0.0
−0.5
−1.0
I
I_t
$E[I_t]$
Fail−to−reject interval
5000
4000
3000
2000
1000
0
cal BP

The I_t time series for the between-region analysis **after omitting coastal regions** (Being Inland counts as inland, `Eval06.1`) is presented below for the last 5000 years:

```
Eval = Eval06.1

plot(
  x = Eval$calBP,
  xlim = plottingXlim,
  xlab = "cal BP",

  y = Eval$observedMoransI.TS,
  ylim = c(-1, 1),
  ylab = "I",
  type = "l",

  main = "Shared-region analysis, inland stratum"
  )
abline(
  h = c(-1, 1), lty = 3
  )
lines(
  x = Eval$calBP,
  y = -1/(rowSums(Eval$whichOn)-1),
  col = "red", type = "s"
  )
lines(
  x = Eval$calBP,
  y = Eval$failToRejectBoundaries[,1],
  col = "red", lty = 2
  )
lines(
  x = Eval$calBP,
  y = Eval$failToRejectBoundaries[,2],
  col = "red", lty = 2
  )
legend(
  "topleft", bty = "n",
  legend = c(
    expression(I[t]),
    expression(paste("E[",I[t],"]")),
    "Fail-to-reject interval"
    ),
  lty = c(1,1,2), col = c("black", "red", "red")
  )
```

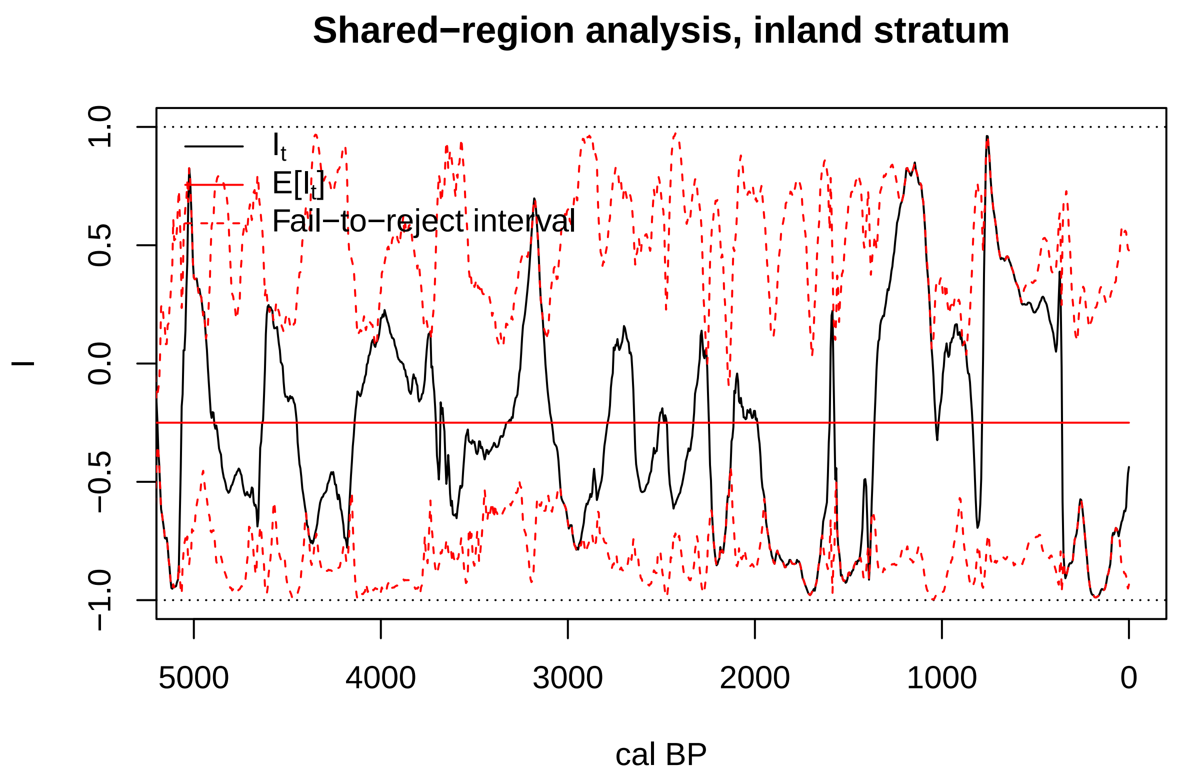
Shared−region analysis, inland stratum
I
1.0
0.5
0.0
−0.5
−1.0
I_t
E[I_t]
Fail−to−reject interval
5000
4000
3000
2000
1000
0
cal BP

The I_t time series for the between-region analysis **after omitting coastal regions** (Being Inland counts as coastal, `Eval06.2`) is presented below for the last 5000 years:

```
Eval = Eval06.2

plot(
  x = Eval$calBP,
  xlim = plottingXlim,
  xlab = "cal BP",

  y = Eval$observedMoransI.TS,
  ylim = c(-1, 1),
  ylab = "I",
  type = "l",

  main = "Shared-region analysis, inland stratum"
  )
abline(
  h = c(-1, 1), lty = 3
  )
lines(
  x = Eval$calBP,
  y = -1/(rowSums(Eval$whichOn)-1),
  col = "red", type = "s"
  )
lines(
  x = Eval$calBP,
  y = Eval$failToRejectBoundaries[,1],
  col = "red", lty = 2
  )
lines(
  x = Eval$calBP,
  y = Eval$failToRejectBoundaries[,2],
  col = "red", lty = 2
  )
legend(
  "topleft", bty = "n",
  legend = c(
    expression(I[t]),
    expression(paste("E[",I[t],"]")),
    "Fail-to-reject interval"
    ),
  lty = c(1,1,2), col = c("black", "red", "red")
  )
```

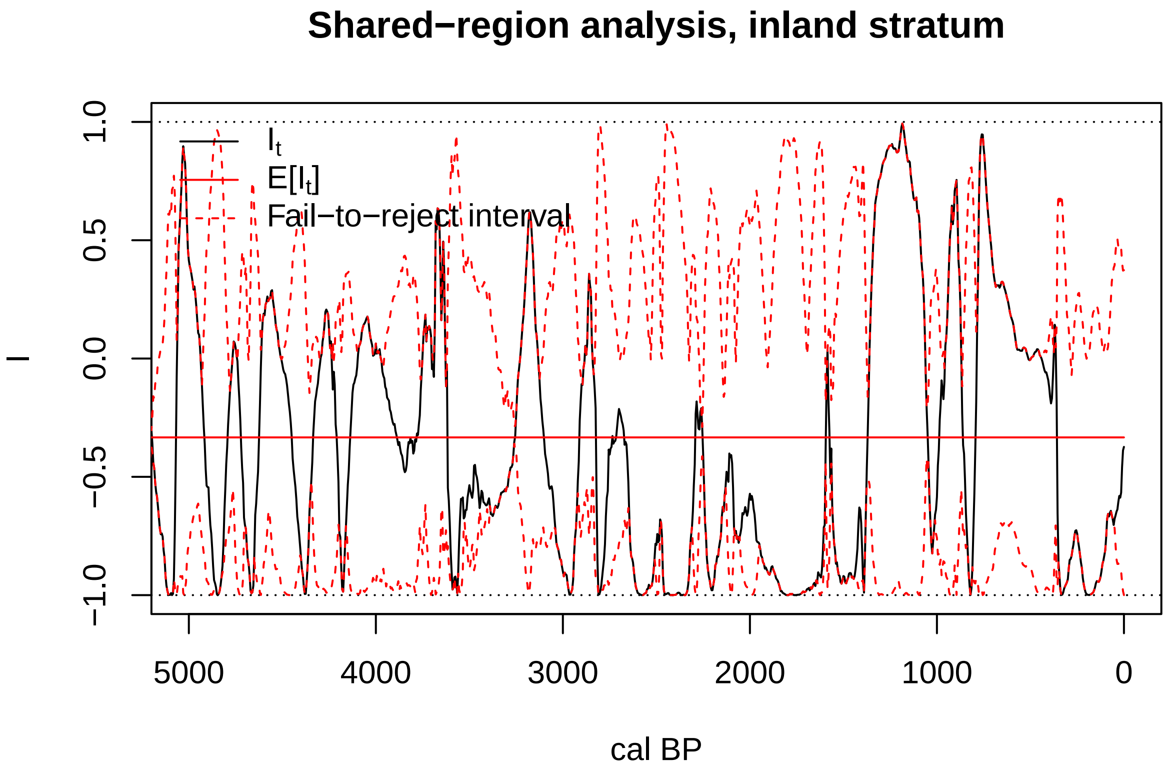

Shared−region analysis, inland stratum
I
1.0
0.5
0.0
−0.5
−1.0
5000
4000
3000
2000
1000
0
cal BP
I_t
$E[I_t]$
Fail−to−reject interval

The I_t time series for the between-region analysis are presented below for the last 5000 years. This regional-contrast analysis is effectively the mirror image of the shared-region analysis (`Eval01`).

```
Eval = Eval02

plot(
  x = Eval$calBP,
  xlim = plottingXlim,
  xlab = "cal BP",

  y = Eval$observedMoransI.TS,
  ylim = c(-1, 1),
  ylab = "I",
  type = "l",

  main = "Between-region analysis"
  )
abline(
  h = c(-1, 1), lty = 3
  )
lines(
  x = Eval$calBP,
  y = -1/(rowSums(Eval$whichOn)-1),
  col = "red", type = "s"
  )
lines(
  x = Eval$calBP,
  y = Eval$failToRejectBoundaries[,1],
  col = "red", lty = 2
  )
lines(
  x = Eval$calBP,
  y = Eval$failToRejectBoundaries[,2],
  col = "red", lty = 2
  )
legend(
  "topleft", bty = "n",
  legend = c(
    expression(I[t]),
    expression(paste("E[",I[t],"]")),
    "Fail-to-reject interval"
    ),
  lty = c(1,1,2), col = c("black", "red", "red")
  )
```

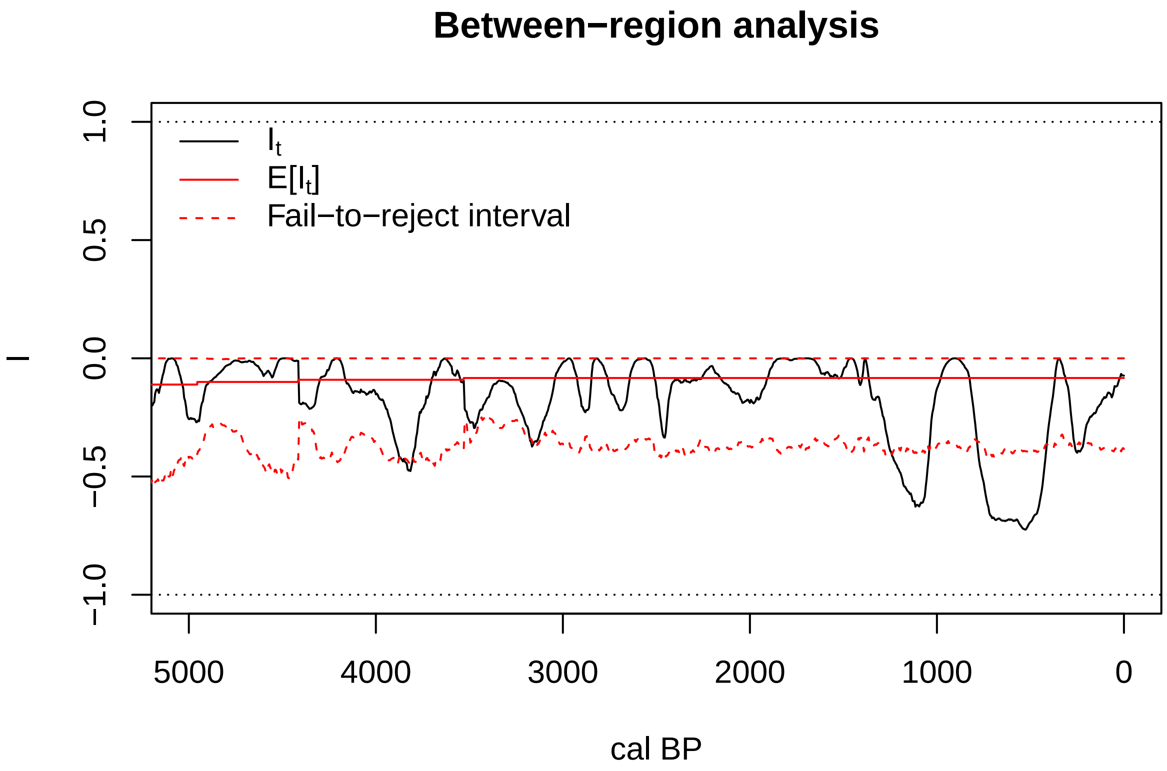
Between−region analysis
I
1.0
0.5
0.0
−0.5
−1.0
I_t
E[I_t]
Fail−to−reject interval
5000
4000
3000
2000
1000
0
cal BP

The I_t time series for the ecological-similarity analysis are presented below for the last 5000 years (Bering inland is treated as inland):

```
Eval = Eval03.1

plot(
  x = Eval$calBP,
  xlim = plottingXlim,
  xlab = "cal BP",

  y = Eval$observedMoransI.TS,
  ylim = c(-1, 1),
  ylab = "I",
  type = "l",

  main = "Eco-similarity analysis"
  )
abline(
  h = c(-1, 1), lty = 3
  )
lines(
  x = Eval$calBP,
  y = -1/(rowSums(Eval$whichOn)-1),
  col = "red", type = "s"
  )
lines(
  x = Eval$calBP,
  y = Eval$failToRejectBoundaries[,1],
  col = "red", lty = 2
  )
lines(
  x = Eval$calBP,
  y = Eval$failToRejectBoundaries[,2],
  col = "red", lty = 2
  )
legend(
  "topleft", bty = "n",
  legend = c(
    expression(I[t]),
    expression(paste("E[",I[t],"]")),
    "Fail-to-reject interval"
    ),
  lty = c(1,1,2), col = c("black", "red", "red")
  )
```

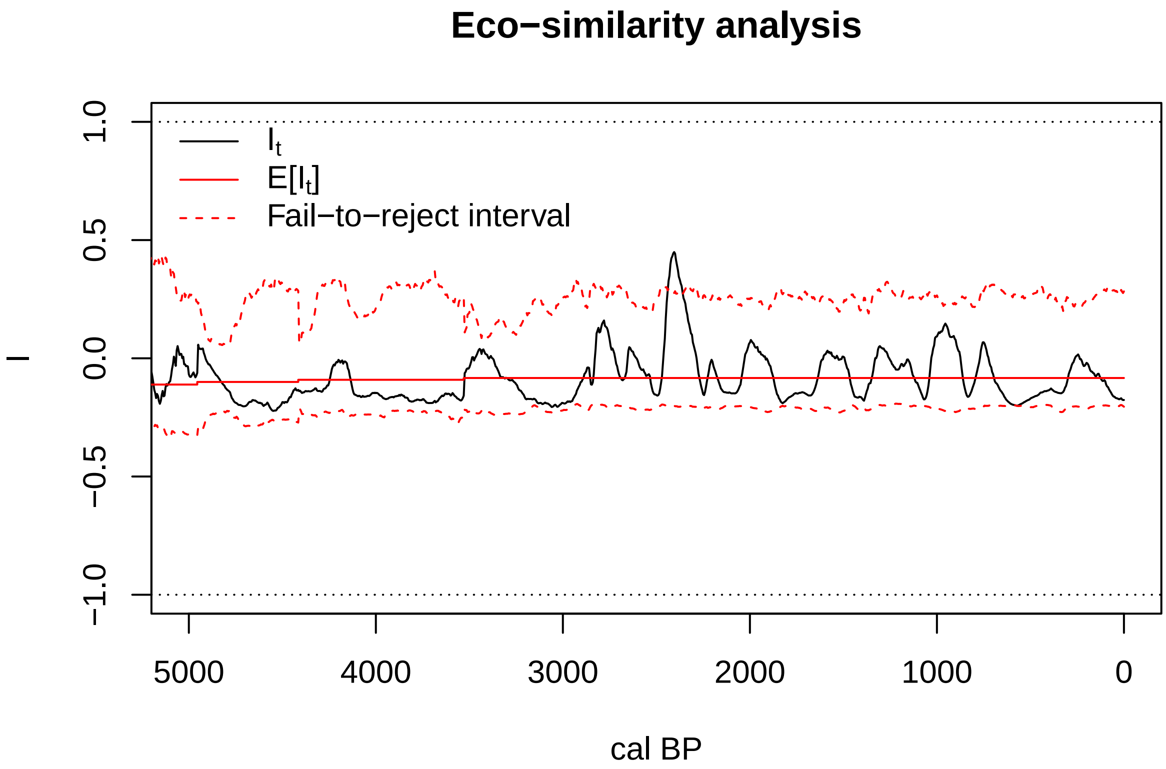
Eco−similarity analysis
I_t
$E[I_t]$
Fail−to−reject interval
1.0
0.5
0.0
−0.5
−1.0
I
5000
4000
3000
2000
1000
0
cal BP

The I_t time series for the ecological-similarity analysis are presented below for the last 5000 years (Bering inland is treated as coastal):

```
Eval = Eval03.2

plot(
  x = Eval$calBP,
  xlim = plottingXlim,
  xlab = "cal BP",

  y = Eval$observedMoransI.TS,
  ylim = c(-1, 1),
  ylab = "I",
  type = "l",

  main = "Eco-similarity analysis"
  )
abline(
  h = c(-1, 1), lty = 3
  )
lines(
  x = Eval$calBP,
  y = -1/(rowSums(Eval$whichOn)-1),
  col = "red", type = "s"
  )
lines(
  x = Eval$calBP,
  y = Eval$failToRejectBoundaries[,1],
  col = "red", lty = 2
  )
lines(
  x = Eval$calBP,
  y = Eval$failToRejectBoundaries[,2],
  col = "red", lty = 2
  )
legend(
  "topleft", bty = "n",
  legend = c(
    expression(I[t]),
    expression(paste("E[",I[t],"]")),
    "Fail-to-reject interval"
    ),
  lty = c(1,1,2), col = c("black", "red", "red")
  )
```

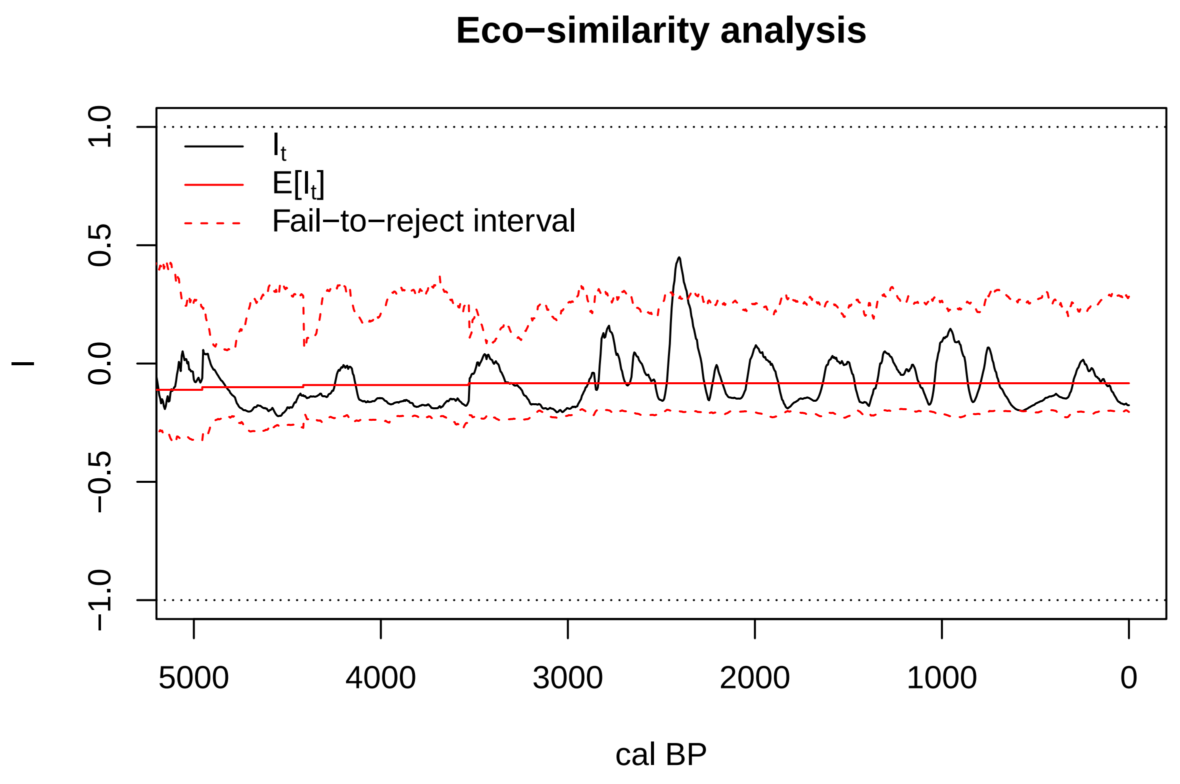

Eco−similarity analysis
1.0
0.5
0.0
−0.5
−1.0
I
I_t
$E[I_t]$
Fail−to−reject interval
5000
4000
3000
2000
1000
0
cal BP

The I_t time series for the adjacency analysis are presented below for the last 5000 years:

```
Eval = Eval04

plot(
  x = Eval$calBP,
  xlim = plottingXlim,
  xlab = "cal BP",

  y = Eval$observedMoransI.TS,
  ylim = c(-1, 1),
  ylab = "I",
  type = "l",

  main = "Adjacency analysis"
  )
abline(
  h = c(-1, 1), lty = 3
  )
lines(
  x = Eval$calBP,
  y = -1/(rowSums(Eval$whichOn)-1),
  col = "red", type = "s"
  )
lines(
  x = Eval$calBP,
  y = Eval$failToRejectBoundaries[,1],
  col = "red", lty = 2
  )
lines(
  x = Eval$calBP,
  y = Eval$failToRejectBoundaries[,2],
  col = "red", lty = 2
  )
legend(
  "topleft", bty = "n",
  legend = c(
    expression(I[t]),
    expression(paste("E[",I[t],"]")),
    "Fail-to-reject interval"
    ),
  lty = c(1,1,2), col = c("black", "red", "red")
  )
```

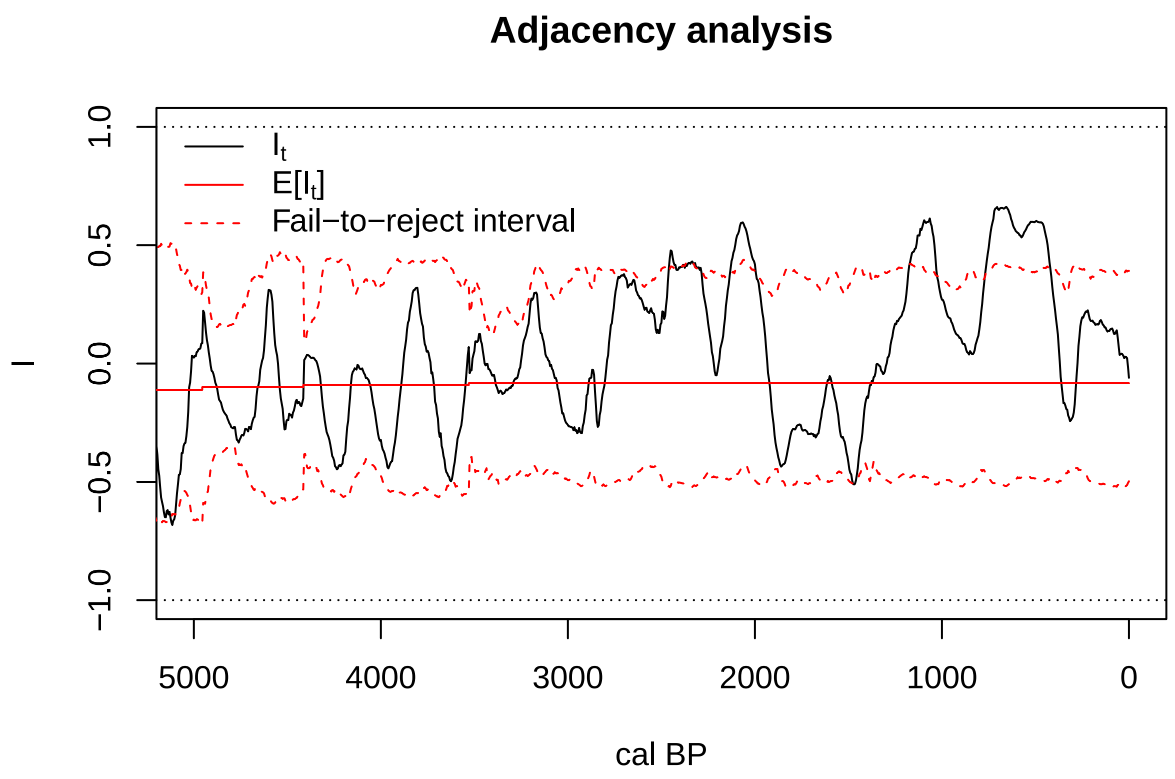
Adjacency analysis
I
cal BP
1.0
0.5
0.0
−0.5
−1.0
5000
4000
3000
2000
1000
0
I_t
E[I_t]
Fail−to−reject interval

The simulated distributions of the global test statistic X^2 are illustrated below for each evaluation. The solid vertical line depicts the observed value, while the dashed vertical line represents the lower boundary of the rejection interval.

```
Eval=Eval01
hist(
  x = Eval$permutedXsq,
  xlim = range(c(
    0, Eval$permutedXsq, Eval$observedXsq,
    Eval$failToRejectBoundariesGlobal
    )),
  xlab = expression("X"^"2"),
  main = "Shared-region analysis, unstratified"
  )
rug(Eval$permutedXsq)
abline(v = Eval$observedXsq, lwd=2)
abline(v = Eval$failToRejectBoundariesGlobal, lty = 2, col = "red")
```

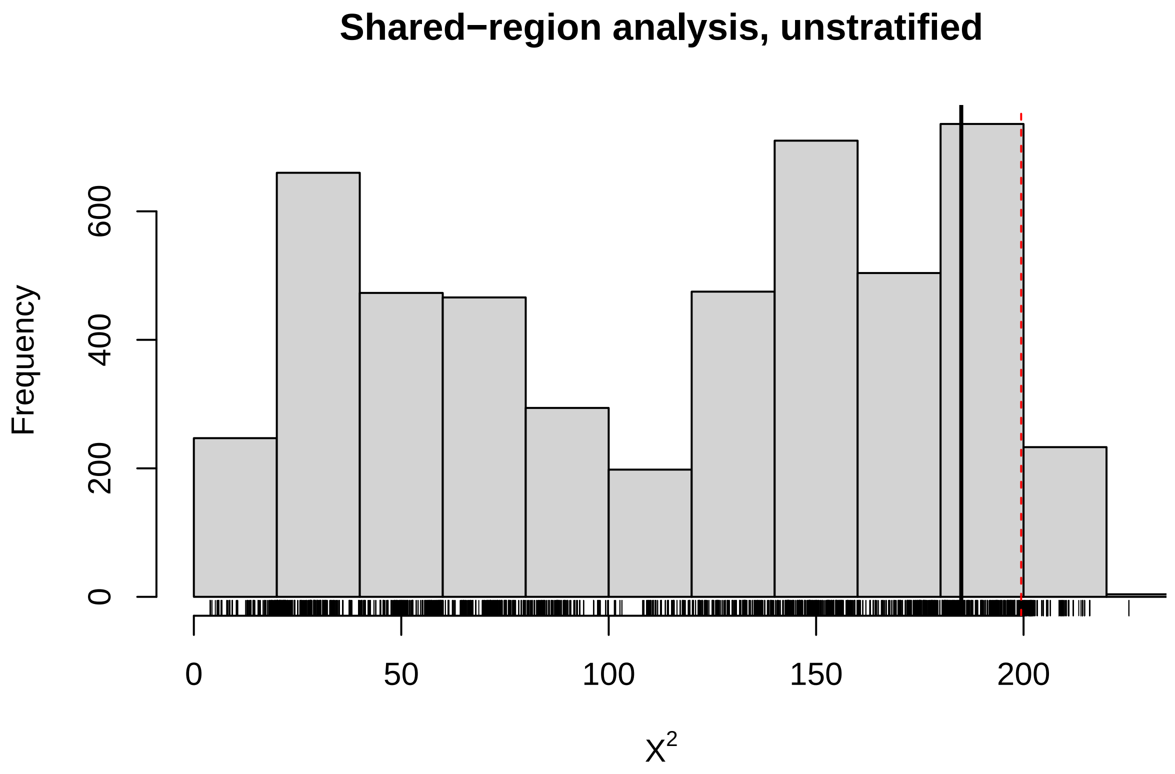


```
Eval=Eval05.1
hist(
  x = Eval$permutedXsq,
  xlim = range(c(
    0, Eval$permutedXsq, Eval$observedXsq,
    Eval$failToRejectBoundariesGlobal
    )),
  xlab = expression("X"^"2"),
```

```r
  main = "Shared-region analysis, coastal stratum"
  )
rug(Eval$permutedXsq)
abline(v = Eval$observedXsq, lwd=2)
abline(v = Eval$failToRejectBoundariesGlobal, lty = 2, col = "red")
```

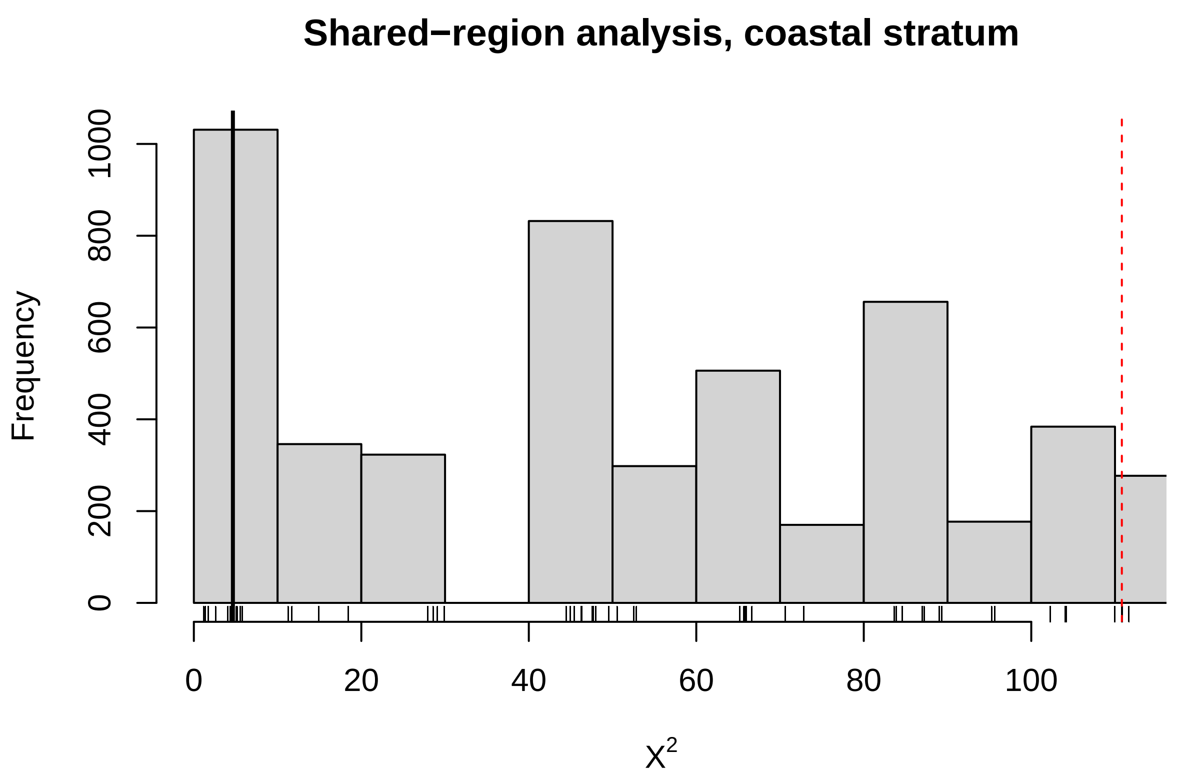


```r
Eval=Eval05.2
hist(
  x = Eval$permutedXsq,
  xlim = range(c(
    0, Eval$permutedXsq, Eval$observedXsq,
    Eval$failToRejectBoundariesGlobal
    )),
  xlab = expression("X"^"2"),
  main = "Shared-region analysis, coastal stratum"
  )
rug(Eval$permutedXsq)
abline(v = Eval$observedXsq, lwd=2)
abline(v = Eval$failToRejectBoundariesGlobal, lty = 2, col = "red")
```

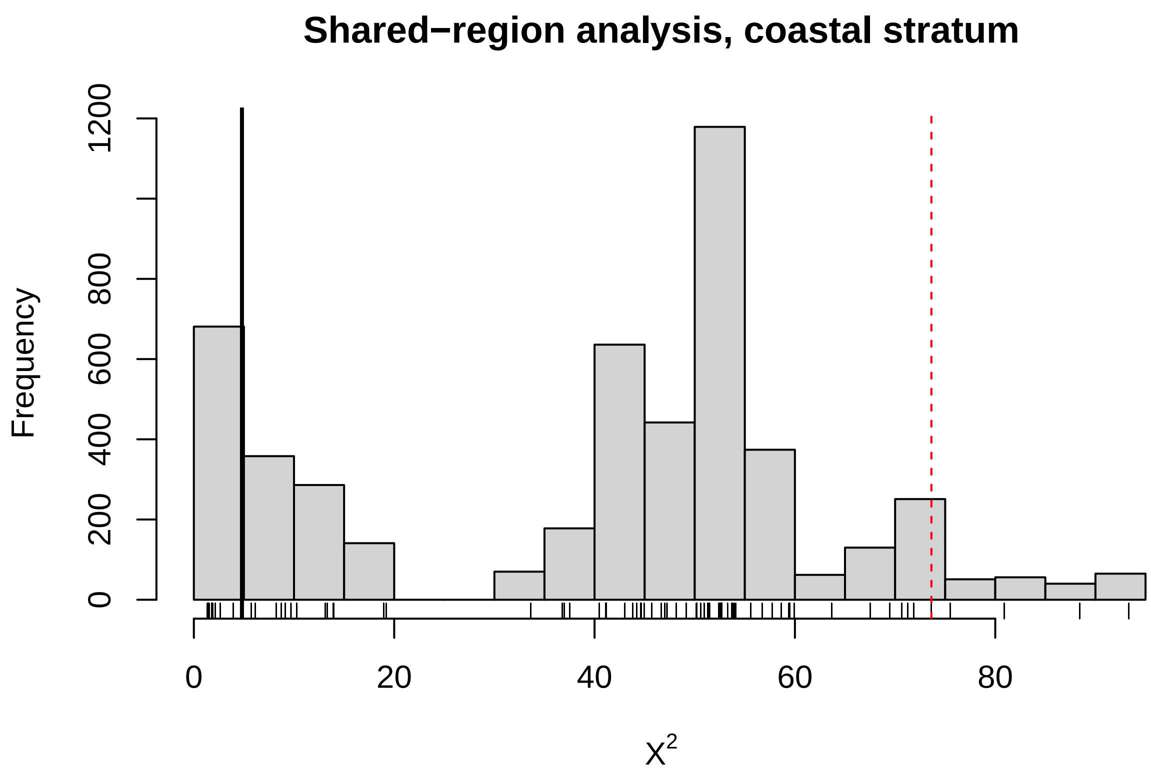


```
Eval=Eval06.1
hist(
  x = Eval$permutedXsq,
  xlim = range(c(
    0, Eval$permutedXsq, Eval$observedXsq,
    Eval$failToRejectBoundariesGlobal
    )),
  xlab = expression("X"^"2"),
  main = "Shared-region analysis, inland stratum"
  )
rug(Eval$permutedXsq)
abline(v = Eval$observedXsq, lwd=2)
abline(v = Eval$failToRejectBoundariesGlobal, lty = 2, col = "red")
```

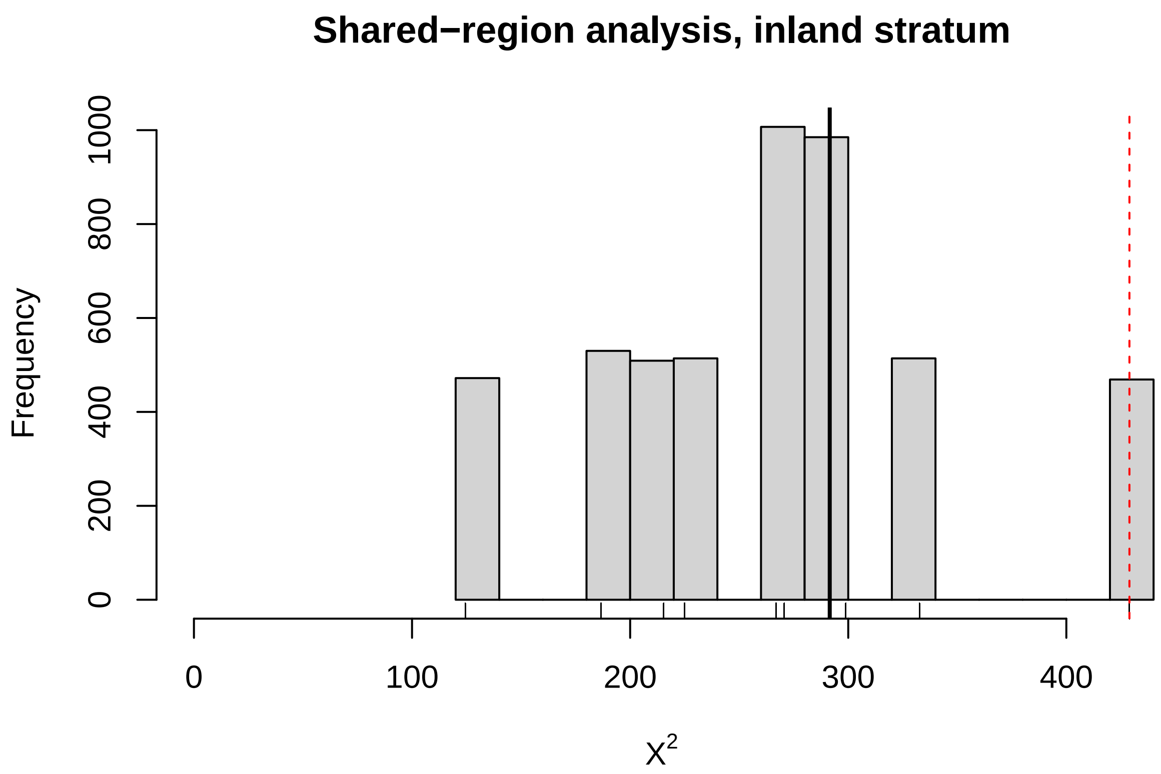


```
Eval=Eval06.2
hist(
  x = Eval$permutedXsq,
  xlim = range(c(
    0, Eval$permutedXsq, Eval$observedXsq,
    Eval$failToRejectBoundariesGlobal
    )),
  xlab = expression("X"^"2"),
  main = "Shared-region analysis, inland stratum"
  )
rug(Eval$permutedXsq)
abline(v = Eval$observedXsq, lwd=2)
abline(v = Eval$failToRejectBoundariesGlobal, lty = 2, col = "red")
```

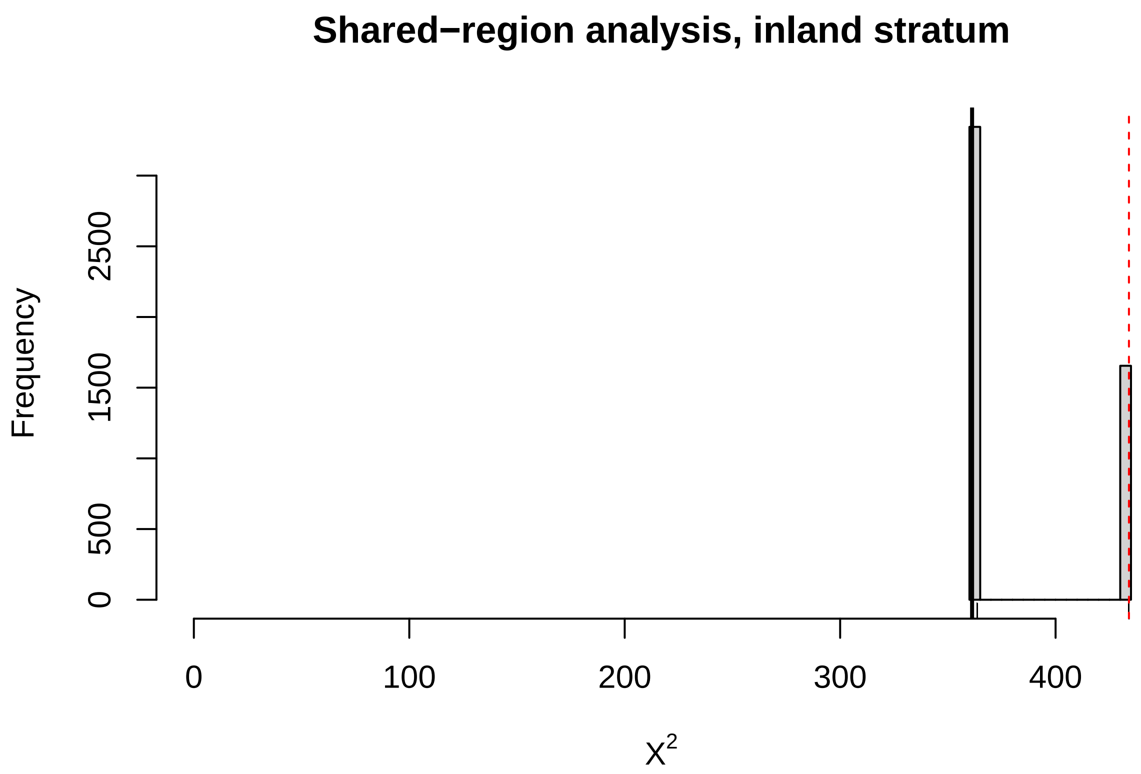


```
Eval=Eval02
hist(
  x = Eval$permutedXsq,
  xlim = range(c(
    0, Eval$permutedXsq, Eval$observedXsq,
    Eval$failToRejectBoundariesGlobal
    )),
  xlab = expression("X"^"2"),
  main = "Between-region analysis"
  )
rug(Eval$permutedXsq)
abline(v = Eval$observedXsq, lwd=2)
abline(v = Eval$failToRejectBoundariesGlobal, lty = 2, col = "red")
```

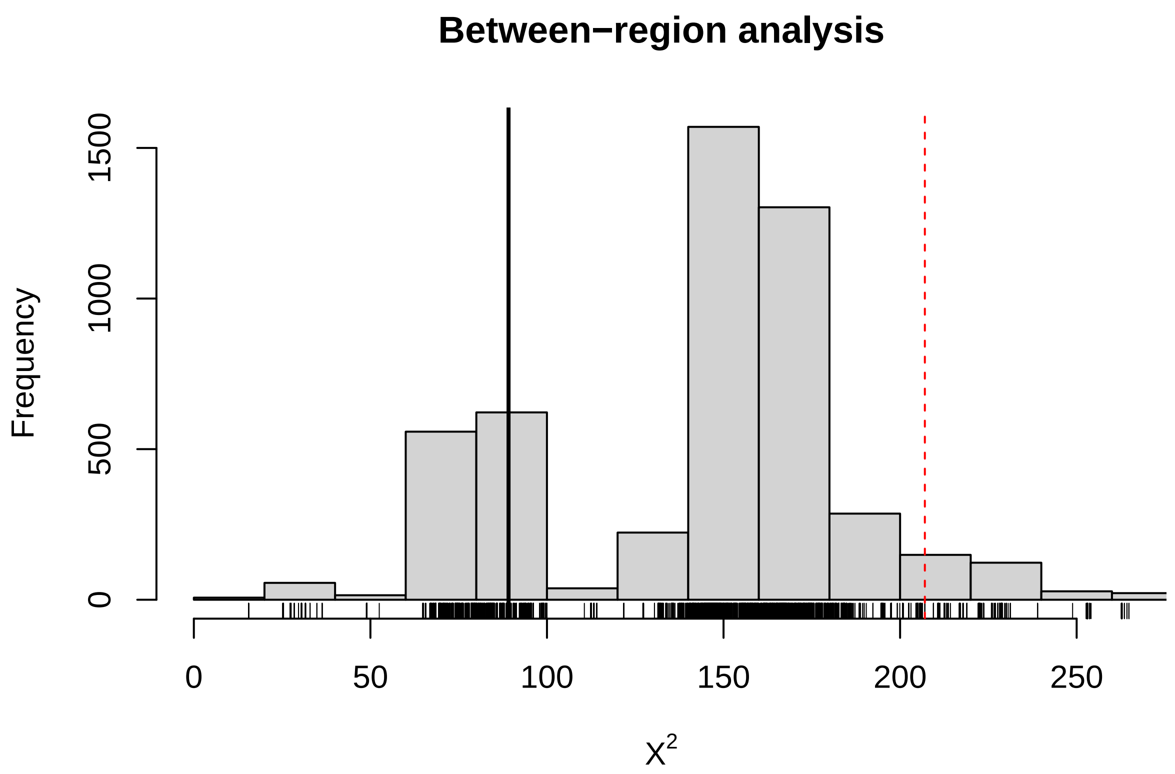


```
Eval=Eval03.1
hist(
  x = Eval$permutedXsq,
  xlim = range(c(
    0, Eval$permutedXsq, Eval$observedXsq,
    Eval$failToRejectBoundariesGlobal
    )),
  xlab = expression("X"^"2"),
  main = "Eco-similarity analysis"
  )
rug(Eval$permutedXsq)
abline(v = Eval$observedXsq, lwd=2)
abline(v = Eval$failToRejectBoundariesGlobal, lty = 2, col = "red")
```

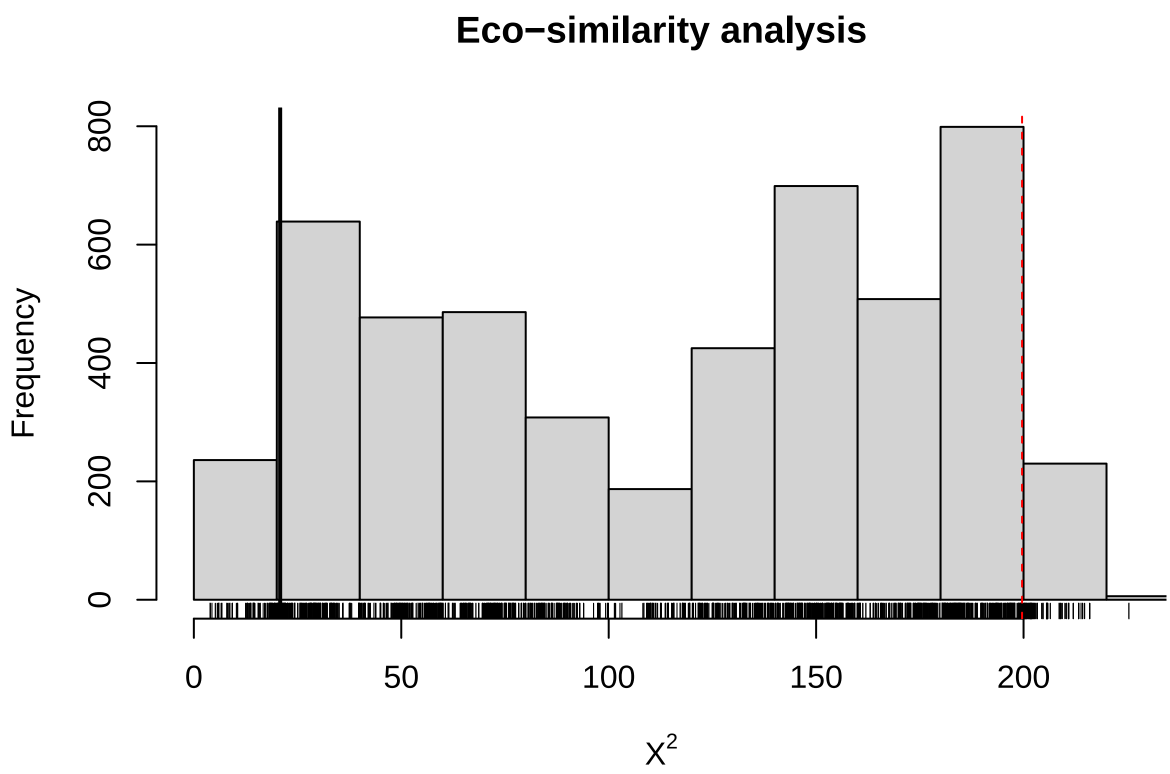


```
Eval=Eval03.2
hist(
  x = Eval$permutedXsq,
  xlim = range(c(
    0, Eval$permutedXsq, Eval$observedXsq,
    Eval$failToRejectBoundariesGlobal
    )),
  xlab = expression("X"^"2"),
  main = "Eco-similarity analysis"
  )
rug(Eval$permutedXsq)
abline(v = Eval$observedXsq, lwd=2)
abline(v = Eval$failToRejectBoundariesGlobal, lty = 2, col = "red")
```

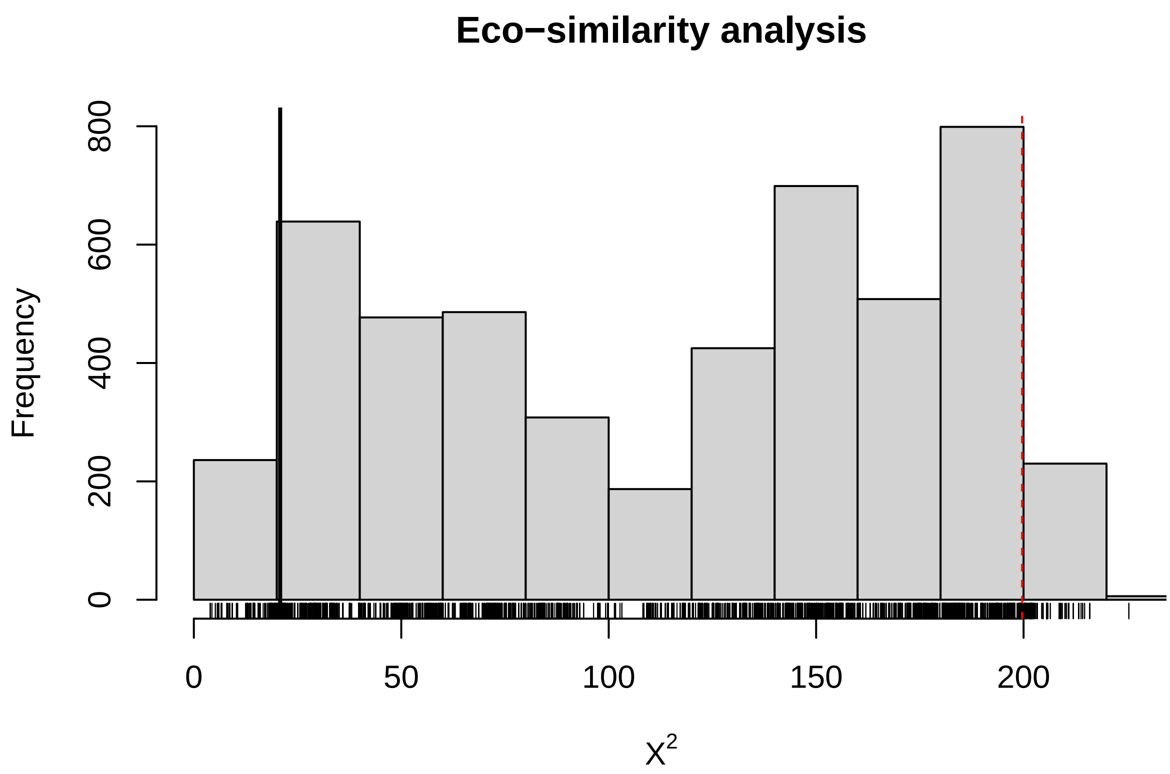


```
Eval=Eval04
hist(
  x = Eval$permutedXsq,
  xlim = range(c(
    0, Eval$permutedXsq, Eval$observedXsq,
    Eval$failToRejectBoundariesGlobal
    )),
  xlab = expression("X"^"2"),
  main = "Adjacency analysis"
  )
rug(Eval$permutedXsq)
abline(v = Eval$observedXsq, lwd=2)
abline(v = Eval$failToRejectBoundariesGlobal, lty = 2, col = "red")
```

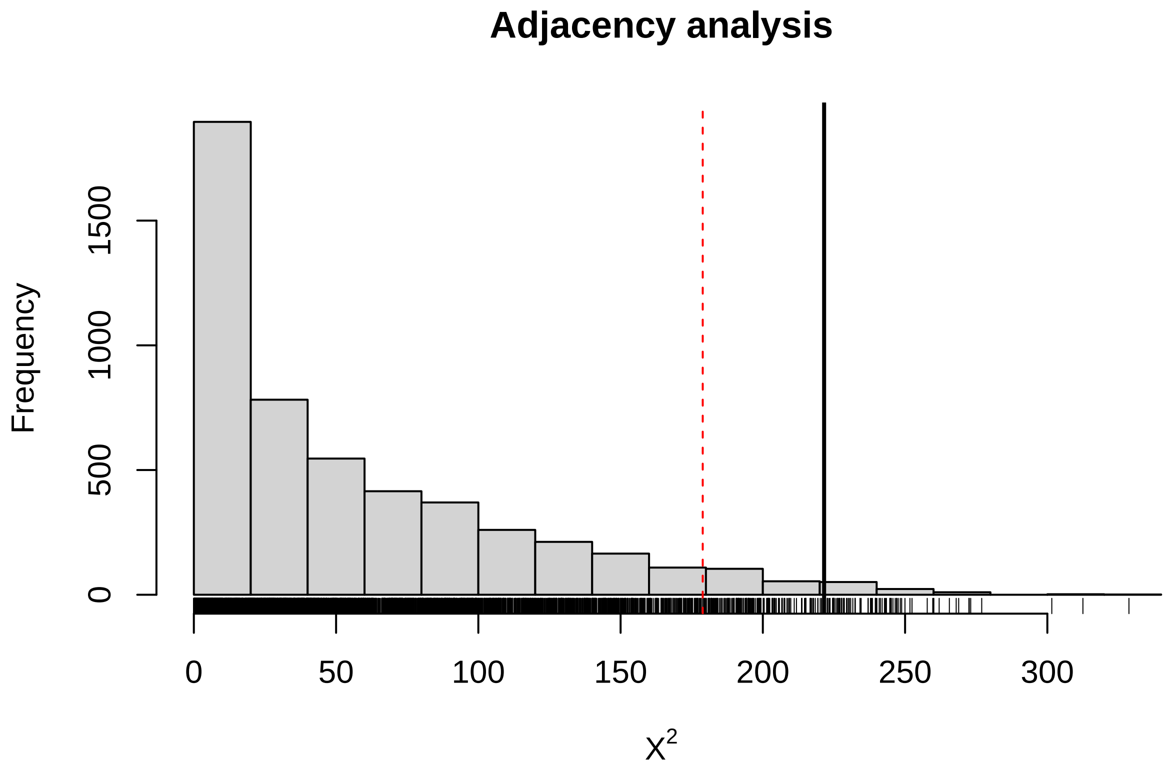
Adjacency analysis
Frequency
0
500
1000
1500
0
50
100
150
200
250
300
X²
